# Optimizing single cell RNA sequencing of stem cells. A streamlined workflow for enhanced sensitivity and reproducibility in hematopoietic studies. The use of human umbilical cord blood-derived hematopoietic stem and progenitor cells

**DOI:** 10.3389/fcell.2025.1590889

**Published:** 2025-05-15

**Authors:** Justyna Jarczak, Patrycja Kieszek, Mariusz Z. Ratajczak, Magdalena Kucia

**Affiliations:** ^1^ Laboratory of Regenerative Medicine, Preclinical Research and Technology Center, Medical University of Warsaw, Warsaw, Poland; ^2^ Stem Cell Institute at Graham Brown Cancer Center, University of Louisville, Louisville, CO, United States

**Keywords:** HSPCs, single-cell RNA seq, clustering, marker detection, UCB, CD34^+^ cells, CD133^+^ cells, CD45^+^ cells

## Abstract

**Background:**

Human hematopoietic stem/progenitor cells (HSPCs) are enriched in umbilical cord blood (UCB) among cell populations that express CD34 and CD133 (PROM1) antigens. These cells can be purified further and sorted by FACS as CD34^+^Lin^−^CD45^+^ and CD133^+^Lin^−^CD45^+^ cells. It has been postulated that the population of CD133^+^ HSPCs is enriched for more primitive stem cells. To address this issue at the molecular level, we performed single-cell RNA-sequencing (scRNA-seq) and analyzed the transcriptome of both cell types. We optimized the available protocols of scRNA-seq of HSPC and described our laboratory experiences with the limited number of cells obtained from human UCB.

**Results:**

Herein, we report the results of scRNA-seq analysis paying special attention to the quality parameters of single cell libraries. We also present the similarities and differences in transcriptome between these cells (CD34^+^Lin-CD45^+^ and CD133^+^Lin-CD45^+^ HSPCs) and their subpopulations identified and visualized as clusters using uniform manifold approximation and projection (UMAP), stressing the need for an integrated analysis of both datasets, which may be merged and treated as “pseudobulk.” We revealed that both populations do not differ significantly in gene expression, as evidenced by the very strong positive linear relationship between these cells (R = 0.99).

**Conclusion:**

To obtain solid results that allow to draw conclusions that would have a biological translation, all parts of the scRNA-seq experiment are crucial and must be carried out with due care: cell sorting, single cell libraries preparation, quality control, and data analysis. The idea of working with sorted material instead of the typical use of a full pellet of blood cells was right and confirmed the possibility of HSPC analysis, even with a limited number of cells.

## Background

Hematopoietic stem/progenitor cells (HSPCs) are specified into cells from all hematopoietic lineages, including erythrocytes, granulocytes, monocytes, lymphocytes, and megakaryocytes (Bhatia et al., 1997; McKinney-Freeman et al., 2012; Morrison et al., 1995; Andrews et al., 1989; Mikkola and Orkin, 2006). Different strategies are currently available to purify these cells; however, isolating them using a single marker with an FACS sorter is impossible (Wognum et al., 2003; Ng et al., 2009; Challen et al., 2009). Therefore, to enrich human bone marrow (BM), peripheral blood (PB), or umbilical cord blood (UCB)-derived HSPCs, a cocktail of antibodies for simultaneous positive and negative marker selection has to be employed (Rossi et al., 2011). HSPCs could be relatively quickly enriched after positive labeling with antibodies against CD34 and/or CD133 and CD45 antigens, along with the depletion of cells expressing lineage differentiation markers (Kato and Radbruch, 1993; Berardi et al., 1995; Notta et al., 2011; Bujko et al., 2019). Panels of cell surface markers including cell clustering, are employed to identify the intermediate stages of the hematopoietic specification. In this concept, HSPCs give rise to progressively lineage-restricted cell types, sometimes depicted as a “hematopoietic tree,” until adult blood cells are reached (Laurenti and Gottgens, 2018; Metcalf, 2007). This historical paradigm has changed in the past 5 years due to multiple studies that have reported the transcriptomes of thousands of individual hematopoietic cells in adult humans and mice, separated by cell surface markers (Calvanese and Mikkola, 2023). It has been suggested that a hematopoietic cell can be “locked” into a specific cell destiny by the stochastic production of lineage-specific transcription factors (TFs) over the noise threshold (Graf and Enver, 2009). It explains why HSPCs have been shown to co-express at low-level genes encoding essential transcription factors (TFs) linked to opposing lineages (Chambers et al., 2007; Hu et al., 1997; Lawrence et al., 2005; Miyamoto et al., 2002). This supports that there are subpopulations of cells in the HSPCs compartment that are permissive to pursue different cell fates before committing to a given lineage - a process known as priming (Ranzoni et al., 2021; Krause, 2002; Milner and Bigas, 1999).

Single-cell RNA-sequencing (scRNA-seq) is a method that analyzes the transcriptome at single-cell and single-base resolution. This method enables unraveling gene expression networks in rare cell types and demonstrates the heterogeneity in gene expression within temporally and spatially separated cell populations (Fast et al., 2021; Oguma et al., 2022; Zheng et al., 2022; Crosse et al., 2020; Farlik et al., 2016). This method (scRNAseq) is becoming an increasingly widely used tool in biological and biomedical research and has advanced the understanding of a range of biological processes and molecular mechanisms occurring in cells. It potentially may have a clinical application and may be valuable in new diagnostic and therapeutic strategies (Su et al., 2022). In the case of hematopoietic stem cells, scRNA-seq allowed to decode both human (Popescu et al., 2019) and mice (Flohr et al., 2021) hematopoiesis. Furthermore, the first hematopoietic stem cells in human embryo were traced by scRNA-seq (Zeng et al., 2019). Somehow, to our surprise, no studies have yet been performed based on scRNA-seq to compare CD34^+^ and CD133^+^ HSPCs from UCB. Therefore, we purified from human UCB a population of CD34^+^Lin^−^CD45^+^ and CD133^+^Lin^−^CD45^+^ HPSCs and subjected these cells to scRNA-seq analysis.

We report the transcriptomic profile of these cells, paying particular attention to the similarities and differences in mRNA expression. Based on this expression, we propose their classification into clusters of cell subpopulations. Importantly, we emphasized the hope that is placed in this type of research and the still existing need to explore the mysteries of the development and functioning of human hematopoietic stem/progenitor cells.

## Materials and methods

### Isolation of human CD34^+^ and CD133^+^ HSPCs

Human umbilical cord blood (hUCB) was obtained from a healthy newborn delivered at the Department of Obstetrics and Gynecology, Medical University of Warsaw (Warsaw Bioethics Committee permission number KB/3/2018) on 14th of September 2023. Participant gave written consent to participate in the study.

hUCB unit was diluted with phosphate-buffered saline (PBS) and carefully layered over Ficoll-Paque (GE Healthcare, Chicago, IL, United States) and centrifuged for 30 min at 400x g at 4°C. The mononuclear cells (MNCs) phase was collected, washed, and used for further analysis. Briefly, cells were stained with the following antibodies: hematopoietic lineage markers (Lin) cocktail of antibodies, each FITC-conjugated: CD235a (clone GA-R2 [HIR2]), anti-CD2 (clone RPA-2.10), anti-CD3 (clone UCHT1), anti-CD14 (clone M5E2), anti-CD16 (clone 3G8), anti-CD19 (clone HIB19), anti-CD24 (clone ML5), anti-CD56 (clone NCAM16.2) and anti-CD66b (clone G10F5) (all BD Biosciences, San Jose, CA, United States); PE-Cy7-conjugated anti-CD45 (clone HI30, BioLegend, San Diego, CA, United States), PE-conjugated anti-CD34 (clone 581, BioLegend, San Diego, CA, United States) and APC-conjugated anti-CD133 (clone CD133, MiltenyiBiotec, Gladbach, Germany). Antibodies were used in the manufacturer’s recommended concentration. Cells were stained in the dark at 4°C for 30 min, then centrifugated and resuspended in RPMI-1640 medium containing 2% fetal bovine serum (FBS, Corning Inc., Corning, NY, United States). Cells were sorted according to the strategy shown in Supplementary Figure S1. Briefly, small events (2–15 μm in size) were included in the “lymphocyte-like” gate (P1) and then further analyzed for the expression of the Lin marker. Lin negative events were gated and subsequently analyzed for the expression of CD45 and CD34 or CD133 antigens (Supplementary Figure S1). Populations of CD34^+^ HSPCs (CD34+Lin−CD45^+^) and CD133+ HSPCs (CD133+Lin−CD45^+^) were sorted on the MoFlo Astrios EQ cell sorter (Beckman Coulter, Brea, CA, United States).

### Single-cell sequencing

After sorting, cells were proceeded directly using Chromium X Controller (10X Genomics, United States) and Chromium Next GEM Chip G Single Cell Kit (10X Genomics, United States). Chromium Next GEM Single Cell 3′ GEM, Library & Gel Bead Kit v3.1, and Single Index Kit T Set A (10X Genomics, United States) were used for library preparation according to manufacturer’s guidelines. Libraries were then pooled and run on Illumina NextSeq 1000/2000 (Illumina, San Diego, CA, United States) in P2 flow cell chemistry (200 cycles) with paired-end sequencing mode (read 1–28 bp, read 2–90 bp, index 1–10 cycles, index 2–10 cycles), assuming 25,000 reads per single cell.

### Bioinformatic analysis

Downstream analysis was performed using Seurat (version 5.0.1), preceded by the 10X Genomics Cell Ranger pipelines (CellRanger version 7.2.0, 10x Genomics, United States, Cell Ranger - Official 10x Genomics Support). Raw sequencing (BCL files) was demultiplexed and converted to fastq files using the bcl2fastq (version v2.20.0.422) within the 10X Genomics Cell Ranger mkfastq pipeline. Then, Cell Ranger count and aggregation pipelines were used for further processing. Cells with less than 200 and more than 2500 transcripts and those with more than 5% of mitochondria-related transcripts were excluded from the analysis. Sequencing reads were mapped to a human genome GRCh38 (version 2020-A) acquired from the 10 × Genomics website (https://www.10xgenomics.com/support/software/cell-ranger/downloads#reference-downloads). Gene expression measurements for each cell were normalized, and the normalized values were log-transformed (“LogNormalize” method) and then reduced to the most highly variable genes. Based on the information described in Brennecke et al. (2013), where authors calculated a subset of features that exhibit high cell-to-cell variation in the dataset, we used the default value of 2000 genes.

Non-linear dimensional reduction to visualize clusters was performed next with the use of uniform manifold approximation and d projection (UMAP) (McInnes et al., 2018) implemented in Seurat (version 5.0.1.) (Satija et al., 2015; Hao et al., 2024). Cell clusters were recognized based on differentially expressed genes, both positive (upregulated genes) and negative (downregulated genes) markers, using an adjusted p-value (padj) (Seurat uses the Wilcoxon rank-sum test by default, padj values are after Benjamini–Hochberg correction). <0.05 and a log2FC > 1. However, for this publication, we focused only on positive ones. These genes were subsequently used for the functional analysis (Gene Ontology terms and KEGG pathways) and characterization of the identified samples and clusters. Functional analysis was performed using the Reactome Pathway Browser (Orlic-Milacic et al., 2024). Analysis was conducted in two ways: independently to thoroughly characterize the samples separately and integrated to visualize the differences between them.

Additionally, we selected hematopoietic lineage differentiation cell markers (CD2, CD3, CD4, CD11b (ITGAM), CD14, CD16 (FCGR3A), CD19, CD34, CD41 (ITGA2B), CD45 (PTPRC), CD66b (CEACAM8), CD68, GYPA, PROM1 (CD133), CD117 (c-KIT) to determine their expression in CD34^+^lin-CD45^+^ and CD133^+^lin-CD45^+^ and confirm the hematopoietic character of the studied cells.

Data analysis was done using R (R Core Team (2021) (R Core Team, 2024), while visualization was performed using the ggplot2 R package [version 3.4.4; (Wickham, 2016)].

### Data availability

The dataset(s) supporting the conclusions of this article are available in the Sequence Read Archive (SRA) (https://www.ncbi.nlm.nih.gov/sra) repository and assigned unique persistent identifiers: PRJNA1128409 (https://www.ncbi.nlm.nih.gov/sra/PRJNA1128409).

## Results and discussion

### Sorting of UCB-purified CD34^+^Lin^−^CD45^+^ and CD133^+^Lin^−^CD45^+^ HSPCs

We used multiparameter cell sorting, which enabled us to purify two subpopulations of HSPCs, CD34^+^Lin-CD45^+^, and CD133^+^Lin-CD45^+^, from hUCB MNCs isolated by Ficoll-Paque gradient centrifugation. Our sorting strategy included depleting Lineage marker-expressing cells, followed by cell isolation based on their simultaneous expression of CD45 and CD34 or CD133 antigen (Abdelbaset-Ismail et al., 2016). Supplementary Figure S1 displays representative dot plots of our sorting strategy.

Briefly, UCB-derived MNCs were immunostained as described in Materials and Methods and then analyzed on a FACS machine. First, objects 2–15 μm in size were included in the “lymphocyte-like cells” gate (P1) (Supplementary Figure S1A). Next, we analyzed the expression of lineage markers and gated negative events (Lin^−^) (Supplementary Figure S1B). Subsequently, we evaluated the expression of CD45 and CD34 (Supplementary Figure S1C) and CD133 (Supplementary Figure S1D) and separately sorted the population of CD34^+^Lin^−^CD45^+^ and CD133^+^Lin^−^CD45^+^ HSPCs.

Additionally, we evaluated the number of CD133^+^Lin^−^CD45^+^ and CD34^+^Lin^−^CD45^+^ HSPCs and analyzed the co-expression of CD34 and CD133 antigens. The results were consistent with our earlier report where we analyzed a large number of hUCB units and calculated that CD34^+^Lin^−^CD45^+^ HSPCs compromised 0.337% (±0.15) of gated events and CD133^+^Lin^−^CD45^+^ HSPC comprised 0.207% (±0.08) of gated cells (Bujko et al., 2023).

Cells isolated from Ficoll-Paque were purified by a cell sorter (MoFlow Astrios) and subjected to droplet-based scRNA-seq (10x Genomics) to investigate their transcriptomic profile and identify cell subpopulations.

### Libraries quality control parameters and sequencing

First, we wanted to evaluate whether having a limited number of sorted cells, how it happens when working with HSPC, we are allowed to prepare libraries meeting the criteria for next-generation sequencing. According to the manufacturer’s instructions, the average fragment length is ∼300–600 bp and the expected insert size is ∼400 bp. Quality control assessed with TapeStation 4150 (Agilent Technologies, United States) showed that all libraries had a fragment length of more than 300 bp and met the standards for sequencing (Supplementary Figures S2A,B). These were libraries prepared from isolated and sorted cell populations of HSPCs: CD133+lin-CD45^+^ (Supplementary Figure S2A), and CD34+lin-CD45^+^ (Supplementary Figure S2A). Therefore, libraries were next employed for quantitative measurements by KAPA Library Quantification Kit (Roche, Switzerland). The library concentrations were as follows: for HSPCs: CD133+lin-CD45^+^ = 173,67 nM and CD34+lin-CD45^+^ = 261,13 nM, with the mean value = 217.40 nM. Of note, sequencing parameters were as follows: Q30 = 93,99%; yield = 50,82 Gb; Aligned = 1,70 and error rate = 0,12. The mean number of reads was around 90 milions per library (Supplementary Figures S2C,D) as follows CD133+lin-CD45^+^ (Supplementary Figure S2C), and CD34+lin-CD45^+^ (Supplementary Figure S2D). Additionally, we captured 5862 CD133+Lin-CD45^+^ and 6811 CD34+Lin-CD45^+^ HSPCs that passed quality control, with a median of 2066 and 1613 genes detected per single cell, respectively (data not shown).

### Single-cell RNA seq analysis of sorted human CD34^+^Lin^−^CD45^+^ and CD133^+^Lin^−^CD45^+^ HSPCs

**FIGURE 1:**
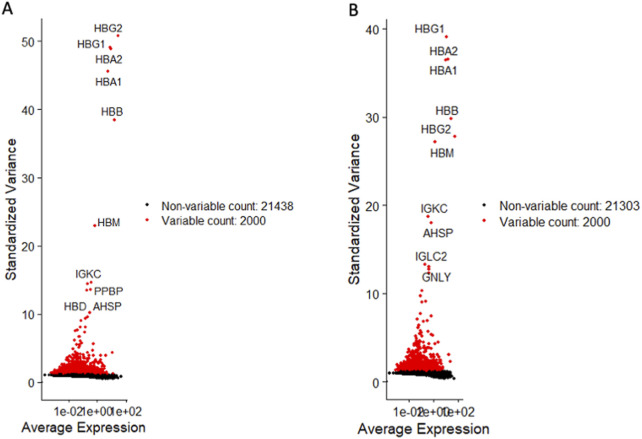
Identification of highly variable genes in both HSPC datasets. Volcano plots display differentially variable genes of CD34+lin-CD45^−^ **(A)** and CD133+lin-CD45^−^ **(B)**. Variable genes are shown in red, while non-variable genes are shown in black.

For CD34^+^Lin^−^CD45^+^, we detected as the most variable genes hemoglobin subunit gamma 2 and gamma 1 (HBG2, HBG1), hemoglobin subunit beta (HBB), hemoglobin subunit mu (HBM), and immunoglobulin kappa constant (IGKC) (Figure 1A). All these genes play a crucial role in hematopoiesis (Bao et al., 2019; Riether et al., 2015). They are involved in various physiological processes, including oxygen transport, hemoglobin assembly, DNA synthesis, mitosis, glycolysis, intracellular cAMP accumulation, and immune recognition. Importantly, we also detected differential expression of the pro-platelet basic protein (PPBP) (Figure 1A), which belongs to the CXC chemokine family and is involved in various cellular processes, including prostaglandin E2 secretion and synthesis of hyaluronic acid and sulfated glycosaminoglycan (Belperio et al., 2000; Broxmeyer and Kim, 1999). PPBP also stimulates the formation and secretion of plasminogen activators by synovial cells (Smith et al., 2015).

Similarly, for CD133^+^Lin^−^CD45^+^ HSPCs, the most differentially expressed genes were HBG1, HBA2, HBA1, HBB, HBM, HBG2, and IGKC (Figure 1B). We also noticed a differential expression of IGLC2, GNLY, and alpha hemoglobin stabilizing protein (AHSP), which encodes a molecular chaperone that binds specifically to free alpha-globin involved in hemoglobin assembly (Weiss et al., 2005). This confirms that our UCB-purified HSPCs express a similar panel of genes involved in oxygen transfer.

### Single-cell cluster analysis for the cell marker expression for sorted UCB CD34^+^Lin^−^CD45^+^ and CD133^+^Lin^−^CD45^+^ HSPCs

**FIGURE 2:**
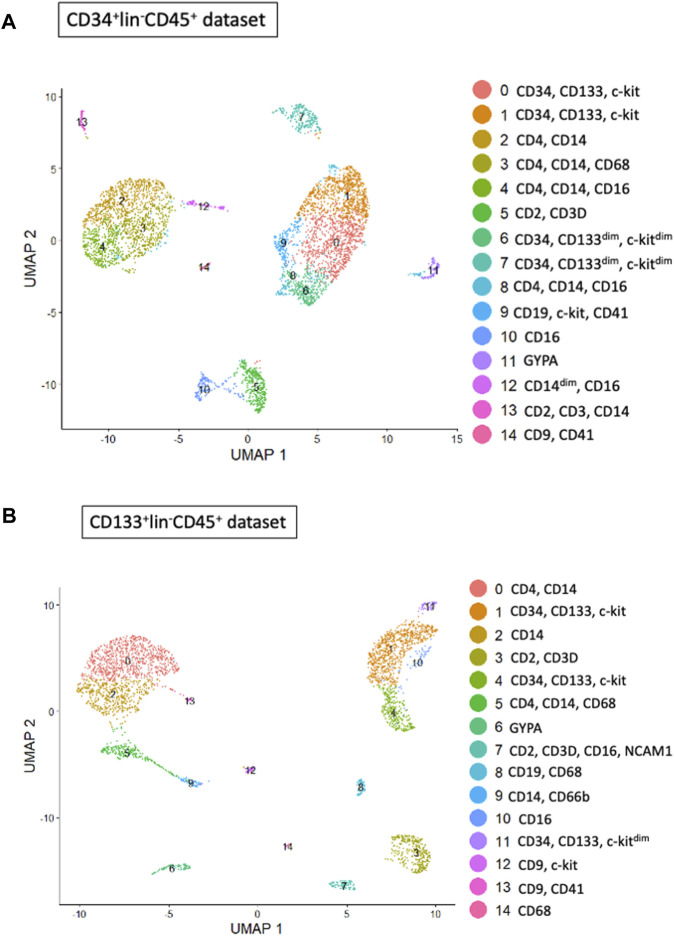
Cell subpopulations (clusters) found in both HSPC datasets. Clusters within CD34+Lin-CD45^+^ **(A)** and CD133+Lin-CD45^+^ **(B)** visualized by UMAP implemented in Seurat (version 5.0.1). The datasets are first analyzed without integration. Human umbilical cord blood isolated HSPCs plot as a total of 14 clusters for CD34^+^ **(A)** and 14 for CD133+ **(B)** HSPCs libraries.

**FIGURE 3:**
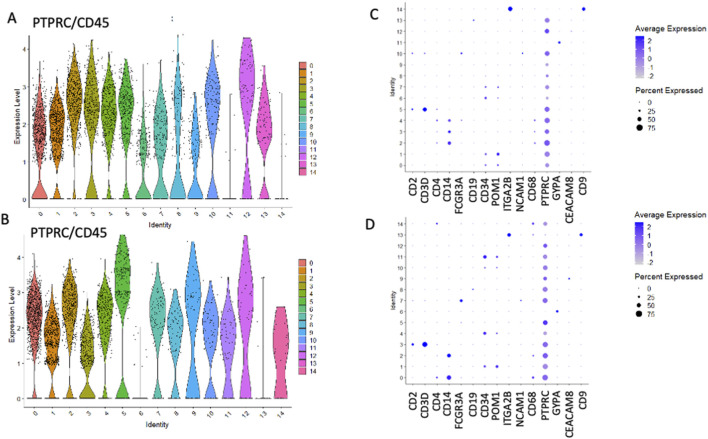
The expression of PTPRC (CD45) and other cell surface marker genes in HSPCs. PTPRC expression was observed in CD34+Lin-CD45^+^ **(A,C)** and CD133+Lin-CD45^+^ **(B,D)**. Volcano plots display the expression of PTPRC in all clusters except clusters 11 and 14 in CD34+Lin-CD45^+^ **(A)** and clusters 6 and 13 in CD133+Lin-CD45^+^ **(B)**. Dot plots displaying the expression of numerous cell markers: CD2, CD3D, CD4, CD14, CD16 (FCGR3A), CD19, CD34, CD133 (PROM1), CD41 (ITGA2B), NCAM1, CD68, CD45 (PTPRC), GYPA, CD66b (CEACAM8) and CD9 in all clusters of CD34+Lin-CD45^+^ **(C)** and CD133+Lin-CD45^+^ **(D)**. Increased PTPRC (CD45) marker expression was confirmed **(C,D)**.

We employed Seurat (version 5.0.1) to perform cluster analysis and differential gene expression within sorted HSPCs populations. Additionally, we used uniform manifold approximation and d projection (UMAP) to investigate cellular heterogeneity, which allowed us to detect a total of 14 clusters for both CD34^+^Lin^−^CD45^+^ (Figure 2A) and CD133^+^Lin^−^CD45^+^ HSPCs libraries, respectively (Figure 2B). As expected, all clusters of CD34^+^Lin^−^CD45^+^ HSPCs except clusters 11 and 14 (Figures 3A,C) and clusters 6 and 13 of CD133+Lin-CD45^+^ HSPCs (Figures 3B,D) express at high-level PTPRC gene (CD45 marker). Within PTPRC (CD45) negative clusters in both sorted HSPCs populations, nearly all cells express glycophorin A (*GYPA* ^*+*^) or tetraspanin (*CD9* ^*+*^) (Figures 3C,D). While GYPA is a marker of erythroid commitment, CD9 has a diverse role in cellular processes, as it has also been shown to trigger platelet activation and aggregation (Huang et al., 2020; Worthington et al., 1990; Miao et al., 2001).

**FIGURE 4:**
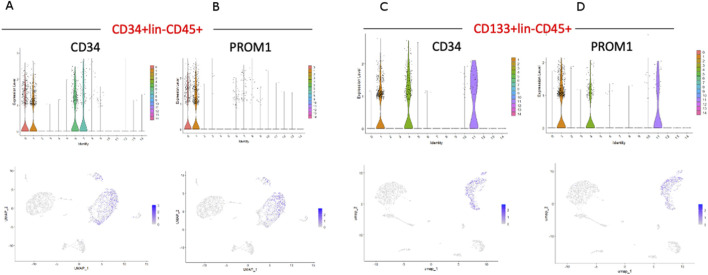
The expression of CD34, and PROM1 (CD133) genes in HSPCs. CD34 and PROM1 expression in CD34+Lin-CD45^+^ **(A,B)** and CD133+Lin-CD45^+^ **(C,D)**. For CD34+Lin-CD45^+^, the expression of CD34 was confirmed in clusters 0, 1, 6, and 7 **(A),** while PROM1 only in clusters 0 and 1 **(B)**. For CD133+Lin-CD45^+^, the expression of CD34 was confirmed in clusters 1, 4, and 11 **(C)**. Simultaneously, the expression of PROM1 was identified in the same clusters **(D)**.

Two stem cell markers, CD34 and PROM1 (CD133), were observed in both populations (Figure 3). While, in CD34^+^Lin^−^CD45^+^ HPSC, the expression of the CD34 gene was observed in clusters no. 0, 1, 6, 7 (Figure 4A), in CD133^+^Lin^−^CD45^+^ HSPCs, the expression of the CD34 gene was observed in clusters no. 1, 4, and 11 (Figure 4C). Interestingly, in the population of CD34^+^Lin^−^CD45^+^ cells, we observed co-expression of mRNA for CD34 and PROM1 in clusters 0 and 1 only (Figures 4A,B). We suggest that these clusters are likely enriched with highly primitive HSPCs co-expressing CD34 and CD133 (PROM1). In contrast, within the CD133^+^Lin^−^CD45^+^ population, clusters 1, 4, and 11 exhibited simultaneous expression of PROM1 and CD34 mRNA (Figures 4C,D). Notably, in CD34^+^Lin^−^CD45^+^ cells, clusters 6 and 7 expressed CD34 mRNA but lacked PROM1 expression. Conversely, in CD133^+^Lin^−^CD45^+^ HSPCs, clusters 1, 4, and 11 showed co-expression of both PROM1 and CD34 transcripts.Importantly, the clusters with confirmed expression of two stem cell markers: CD34 and PROM1 (CD133) will be called later in the text as primitive or quiescent clusters.

**FIGURE 5:**
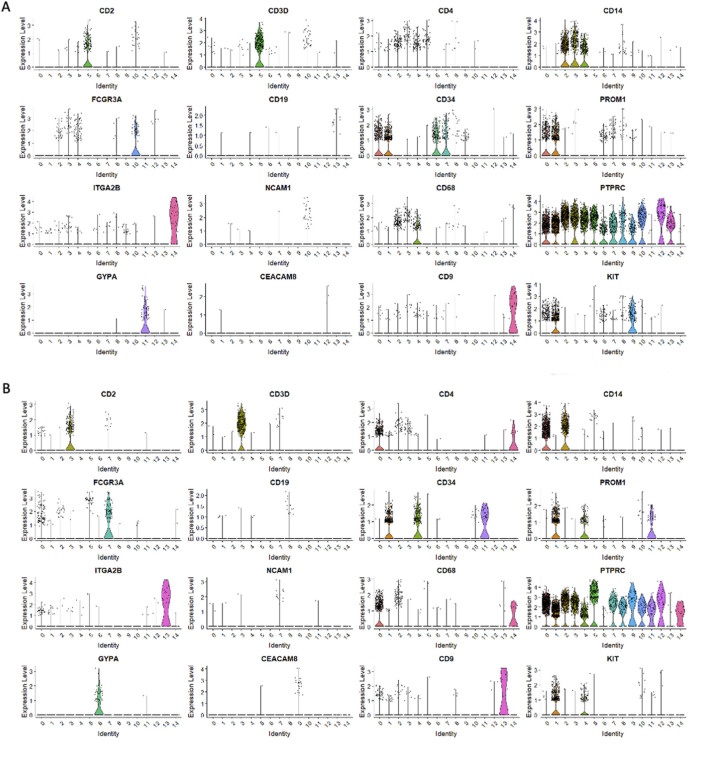
Expression of cell markers in CD34^+^Lin^-^CD45^+^ and CD133^+^Lin^-^CD45^+^ HSPCs. . Violin plots displaying the expression of numerous cell markers: CD2, CD3D, CD4, CD14, CD16 (FCGR3A), CD19, CD34, CD133 (PROM1), CD41 (ITGA2B), NCAM1, CD68, CD45 (PTPRC), GYPA, CD66b (CEACAM8), CD9 and KIT (CD117) in all clusters of CD34^+^Lin^−^CD45^+^. **(A)** and CD133^+^Lin^−^CD45^+^ **(B)**.

Next, we analyzed the remaining cell markers related to hematopoietic lineage specification: (CD2, CD3, CD4, CD11b (ITGAM), CD14, CD16 (FCGR3A), CD19, CD34, CD41 (ITGA2B), CD45 (PTPRC), CD66b (CEACAM8), CD68, GYPA, PROM 1 (CD133), CD117 (c-KIT) (Figures 3C,D; Figures 5A,B). The clusters with confirmed expression of lymphocyte (T and B), monocytes, macrophages, NK cells and granulocytes markers (e. g. CD2, CD3, CD19, CD14, FCGR3A, CD68) were called ‘fate decision’ clusters. In sorted CD34^+^Lin^−^CD45^+^ HSPCs, T lymphocyte markers (CD2, CD3) were detected in clusters no. 5 and 13 and CD4 in clusters 2, 3, 4, and 5. In contrast, for CD133^+^Lin^−^CD45^+^ HSPCs, CD2, and CD3 were detected in cluster no. 3 and 7, and CD4 in clusters 1, 2, 3, and 4. B lymphocyte marker (CD19) was expressed in cluster no. 13 for CD34^+^Lin^−^CD45^+^ HSPC and cluster 8 for CD133^+^Lin^−^CD45^+^ HSPC. The expression of mRNA for monocytes, macrophages, NK cells, and granulocytes (CD14, FCGR3A, and CD68) was present in clusters 2, 3, and 4 (CD14), clusters 3, 4, and 10 (FCGR3A), clusters 2, 3 and 10 (CD68) of CD34^+^Lin^−^CD45^+^ HSPCs. In the case of CD133+Lin-CD45^+^ HSPCs, CD2, and CD3 were expressed in cluster 3 and CD4 in clusters 0, 2, and 3 CD19 was detected in clusters 8, CD14 in clusters 0 and 2, FCGR3A in clusters 0, 5, 7, and CD68 in clusters 0 and 2. The erythrocyte marker glycophorin A (GYPA) was found in cluster 11 for the CD34^+^Lin^−^CD45^+^ dataset and cluster 6 for the CD133^+^Lin^−^CD45^+^ HSPCs dataset. At the same time, mRNA of megakaryopoietic marker - ITGA2B (CD41) detected in clusters 14 of CD34^+^Lin^−^CD45^+^ HSPC and 13 of CD133^+^Lin^−^CD45^+^ HSPC (Figures 5A,B; Figures 3C,D). We also identified in both studied datasets the expression of mRNA for c-KIT (CD117) that, to a large extent, correlated with the expression of mRNA for CD34 and CD133 (Figures 5A,B).

In addition to markers for hematopoietic specification of sorted cells toward erythropoiesis, myelopoiesis, and thrombopoiesis, we also identified changes in the expression of genes involved in innate immunity responses. We observed, for example, the downregulation of S100A8 and S100A9 in numerous clusters of CD34+Lin-CD45^+^ cells (clusters 1, 5, 6, 7, 9, 10, 11, and 13) (Supplementary Tables S1–S9). However, at the same time, the expression of these markers was upregulated in clusters 2 and 3 (Supplementary Tables S10, S11). S100A8 and S100A9 belong to the S100 protein family, also known as myeloid-related proteins (MRPs), and are primarily expressed in myeloid lineage cells, including neutrophils, monocytes, and macrophages (Xia et al., 2017; Takizawa et al., 2012). They belong to the alarmin family of molecules.

We observed a similar situation in the expression of S100A8 and S100A9 in CD133+Lin-CD45^+^ cells. Among the top 50 downregulated genes in many clusters, we found other proteins from the family S100 proteins, such as S100A6, S100A10, S100A11, and S100A12. Decreased expression of S100A12 was observed in clusters 1, 3, 4, 7, 8, 9 and 11 (Supplementary Tables S12–S18). This protein, also known as Calgranulin C or EN-RAGE (Extracellular Newly identified RAGE-binding protein), is primarily involved in inflammatory responses and host defense mechanisms, and is expressed and secreted by neutrophils, monocytes, and macrophages in response to inflammatory stimuli (Cheng et al., 2019).

The summary of progenitor fractions and their lineage commitment in the single-cell clusters of CD34+Lin-CD45^+^ and CD133+Lin-CD45^+^ HSPCs is presented in Table 1.

**TABLE 1:** The summary of progenitor fractions and their lineage commitment in the single-cell clusters of CD34+Lin-CD45^+^ and CD133+Lin-CD45^+^ HSPCs.

Lineage commitment	Cluster name	CD34+lin-CD45^+^clusters	CD133+lin-CD45^+^clusters	Key markers
Primitive HSPCs	Quiescent/primitive	0, 1, 6, 7	1, 4, 11	CD34, PROM1
Early progenitors		0, 1, 6, 9	1, 4	c-KIT
T-cell lineage		2, 3, 4, 5	0, 2, 3	CD2, CD3, CD4
B cell lineage		13	8	CD19
Myeloid lineage	Fate decision	2, 3, 4, 10	0, 2, 7, 14	CD14, FCGR3A, CD68
Erythroid lineage		11	6	GYPA
Megacaryocytic lineage		14	13	ITGA2B

### Transcriptomic profiles of selected clusters of UCB CD34+Lin-CD45^+^ and CD133+Lin-CD45^+^ HSPCs

#### Transcriptomic profiles of quiescence/primitive clusters (expressing PROM1, CD34 genes)

Next, we analyzed the transcriptomic profile of selected clusters in CD34+Lin-CD45^+^ HSPCs. Firstly, we focused on the transcriptomic differences between clusters expressing mRNA CD133 (PROM1) and CD34 *versus* clusters expressing CD34 only. This comparison was possible only for the CD34+Lin-CD45^+^ HSPC population because, in these cells, we can observe these two subpopulations positive for both genes encoding PROM1 and CD34. Therefore, we compared clusters 0 and 1 (expressing mRNA for CD34 and PROM1 and clusters 6 and 7 (expressing mRNA for CD34 only) (Supplementary Table S19).

Among the TOP 50 upregulated genes, the expression of two genes linked with developmental processes was increased almost 3 times in CD34 and PROM1 expressing clusters compared to the clusters with the only expression of CD34. The first one was SOX5, which is responsible for the embryonic development regulation (log2FC = 2.77; padj = 2.32E-05) (Lai et al., 2008), and the second one, MECOM, responsible for embryonic development, cell differentiation, and regulation of transcription (log2FC = 2.71; padj = 9.49E-28) (Supplementary Table S19) (Goyama et al., 2008). Interestingly, there were several genes downregulated at the level from −5 to – 10 log fold change, e.g., PTGDS (log2FC = −10.62; padj = 2,02E-05) regulating inflammation; DNTT (log2FC = −9.42; padj = 5.42E-21), which is crucial in the process of generating a diversity of antigen receptors for effective immune response; IGLC1 (log2FC = −7.78; padj = 0.036) and IGLC2 (log2FC = −7.08; padj = 5.33E-07 triggering complement by antibody-mediated activation (Supplementary Table S19). The list of TOP 50 up and downregulated genes is presented in Supplementary Table S19. As mentioned above, such comparison was not possible for CD133+Lin-CD45+HSPC because, within this population, we can observe clusters expressing both markers: CD34 and PROM.

#### Transcriptomic profiles of quiescent *versus* “fate decision” clusters

Next, we compared datasets of primitive clusters of CD34+Lin-CD45^+^ and CD133+Lin-CD45^+^ HSPCs, that express mRNA for both CD34 and CD133 markers, with the selected clusters showing already lineage specification. As first, we compared CD34+Lin-CD45^+^ HSPCs datasets, clusters no. 0, 1, 6, and 7 (expressing CD34, PROM1 and KIT (CD117)) with clusters no. 2, 3, 4 (expressing CD4, CD14, CD68) (Figure 5A; Supplementary Table S20). We noticed increase in expression of several genes in clusters 0, 1, 6 and 7 of connected with developmental processes (DPPA4, log2FC = 8.03; padj = 5.42E-56), cell differentiation and proliferation (MYCT1, log2FC = 8.99; padj = 7.52E-36; MPDZ, log2FC = 8.85; padj = 5.20E-30; MAGI1, log2FC = 8.27; padj = 5.18E-16 as well as KIF7, log2FC = 8.16; padj = 2.71E-19) (Supplementary Table S20). DPPA4 gene is present in embryonic stem cells and is likely to play a role in maintaining their pluripotency (Abu-Dawud et al., 2022; Li et al., 2021; Somanath et al., 2018). This somehow surprising increase in expression of this gene in a primitive subpopulation of CD34+Lin-CD45^+^ HSPCs requires further studies. Among TOP 50 upregulated genes, we also found: CFH (log2FC = 9.02; padj = 8.89E-34), playing an important role in regulation of the complement cascade as well as MAPK12 (log2FC = 7.96; padj = 7.54E-16), involved in signaling pathways related to stress, inflammation, and apoptosis (Supplementary Table S20). This supports a potential interaction of CD34+Lin-CD45^+^ HSPC with the immune system. Of note the expression of CD34 gene was highly upregulated in this dataset (log2FC = 8.44; padj = 7.54E-16) (Supplementary Table S20). In contrast, among TOP 50 downregulated genes, we found those connected with monocytes, macrophages and lymphocyte specification including CX3CR1 (log2FC = -10.75; padj = 2.16E-81), FCGR3A (log2FC = −10.60; padj = 2.79E-35), CD300E (NKG2D) (log2FC = - 10.04; padj = 6.74E-130), TLR8 (log2FC = −9.75; padj = 5.79E-38), and CLEC4D (log2FC = - 9.50; padj = 1.21E-25) (Supplementary Table S20).

The same comparison has been performed for CD133+Lin-CD45^+^ HSPCs clusters 1, 4 and 11 (expressing CD34, PROM1 and KIT *versus* clusters no. 0, 2 and 14 (expressing CD4, CD14, CD68) (Figure 5B; Supplementary Table S21). Similarly, to the population of CD34+Lin-CD45^+^ HSPCs, we found genes connected with developmental processes including MEST (log2FC = 9.08; padj = 1.07E-56) as well as those connected to cell differentiation: LRP6 (log2FC = 8.80; padj = 2.99E-37) and FZD6 (log2FC = 8.50; padj = 1.85E-34) (Supplementary Table S21). These are both frizzled receptors and key mediators of Wnt signaling pathways. Moreover, in primitive subpopulations of CD133+Lin-CD45^+^ HSPCs we identified genes with increased expression, involved in chromatin modification and regulation of gene expression such as HIST1H2BF (Histone H2B type 1-C/E/F/G/I) (log2FC = 8.85; padj = 6.00E-42); cell signaling - FGD5 (log2FC = 8.73; padj = 1.23E-41) and RNF150 (log2FC = 8.69; padj = 1.32E-32), as well as those involved in intracellular transport: TCTEX1D1 (log2FC = 8.51; padj = 1.57E-33) (Supplementary Table S21).

#### Transcriptomic comparison of both cell populations

If we compare the TOP 50 upregulated genes from CD34+Lin-CD45^+^ HSPC with CD133+Lin-CD45^+^ HSPCs datasets, we will find four common genes (CD34, SCN3A, COL6A2, and SOCS2-AS1) with increased expression in primitive subpopulations *versus* clusters showing a higher level of differentiation (Supplementary Tables S20, S21). Among these genes, SOCS2-AS1 as long non-coding RNA (lncRNA) and antisense transcript of the SOCS2 gene regulates its expression through various mechanisms such as RNA interference or chromatin modification. As reported, the SOCS2 gene acts as a negative regulator of signaling pathways in controlling cell proliferation, differentiation, and functioning of immune cells (Cramer et al., 2022; Rico-Bautista et al., 2006). Analysis of TOP50 downregulated genes revealed mostly genes connected with monocytes, macrophages and lymphocytes functions including CLEC4E (log2FC = −10.44; padj = 9.46E-68), CCL3 (log2FC = −9.37; padj = 1.42E-18) and CD86 (log2FC = −9.18; padj = 3.57E-90), TLR8 (log2FC = −8.94; padj = 1.01E-28) and CLEC4D (log2FC = −8.64; padj = 1.89E-17) (Supplementary Table S21). Moreover, mRNA for genes CSTA, VCAN, LGALS2, PID1, SDC2, SERPINB2, TLR8, CLEC4D, LAMB3, and CYP1B (Supplementary Tables S20, S21) was downregulated in primitive subpopulations of CD34+Lin-CD45^+^ and CD133+Lin-CD45^+^ HSPCs, Interestingly, among these genes VCAN, LGLS2 and SDC2 are involved in cell-cell communication.

### Unsupervised clustering to analyze transcriptional states in CD34+Lin-CD45^+^ and CD133+Lin-CD45^+^ HSPCs

Analysis of differentially expressed genes in CD34+Lin-CD45^+^ dataset revealed upregulation of several genes in CD34+PROM1+c-KIT + cells (clusters 0 and 1) including C1QTNF4 (log2FC = 1.35; padj = 3.48E-159), AVP, SNHG29, AREG, SPINK2, CALN1, NKAIN2 and FAM30A (Supplementary Table S22). These genes are involved in cell metabolism (C1QTNF4, SPINK2), immunity (C1QTNF4, SPINK2), and several cellular processes (SNHG29, AREG, FAM30A). Disruption of their expression is associated with leukemia and the development of lymphoma. Top downregulated genes detected for clusters 0 and 1, including LYZ, VCAN, TYROBP, NAMPT, and SAT1, are related to immune responses and cellular processes.

**FIGURE 6:**
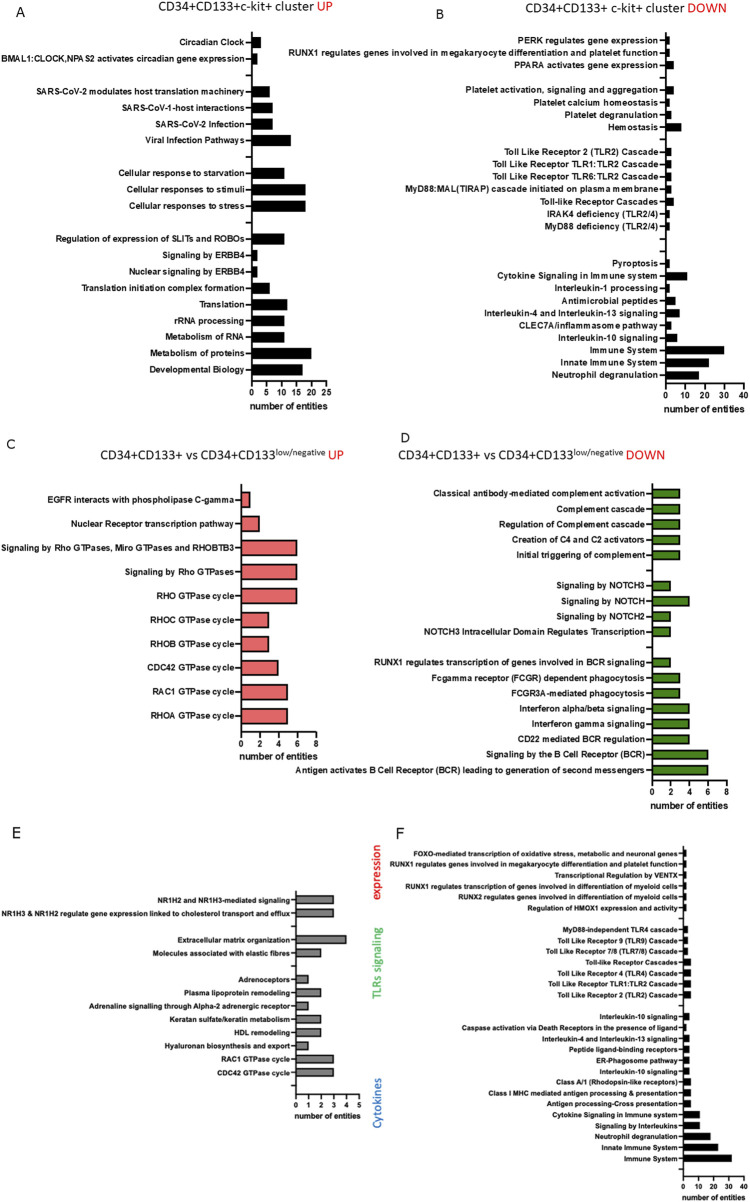
Reactome pathways identified based on the top 50 upregulated **(A)** and top 50 downregulated **(B)** genes were found as differentially expressed between clusters 0 and 1 (expressing CD34, PROM1, and KIT), and clusters 6 and 7 (expressing only CD34) of CD34^+^Lin^−^CD45^+^. Reactome pathways were identified based on the top 50 upregulated **(C)** and top 50 downregulated **(D)** genes found in clusters 1 and 4 (expressing CD34, PROM1, and KIT) of CD133+Lin-CD45^+^. Reactome pathways were identified based on the top 50 upregulated **(E)** and top 50 downregulated **(F)** genes in a primitive subpopulation of PROM1+CD34^+^ cells of the CD133+Lin-CD45^+^ HSPCs dataset.

Next, by employing Reactome and the gene ontology, we identified several pathways highly up (Figure 6A) and downregulated (Figure 6B) in a cluster of CD34+Lin-CD45^+^ that express PROM1 and c-KIT. These top 50 upregulated genes belong to circadian clock regulation, cellular responses to stress, transcription, translation, and metabolism of RNA and proteins. We also confirmed our previously published data that SARS-CoV2 might infect HSPCs because among the top 50 highly expressed genes in scRNA-Seq of CD34+PROM1+c-Kit + dataset revealed those related to SARS-CoV2 infection (Kucia et al., 2021; Ropa et al., 2020). In parallel, in the same dataset, we observed downregulation of genes annotated to inflammation, cytokine signaling, Toll-like receptor signaling, caspase 3 dependent cell death - pyroptosis, platelet activation, and hemostasis (Figure 6B).

Finally, we compared the expression of top upregulated (Figure 6C) and downregulated (Figure 6D) genes between a subpopulation of CD34+PROM1 *versus* CD34+PROM1low/negative among CD34+Lin-CD45^+^ HSPCs. Reactome and GO analysis revealed upregulation of genes involved in transcription and signaling by Rho GTPase, while among the top downregulated genes were the ones associated with complement cascade activation and NOTCH and interferon signaling.

Analysis of differentially expressed genes - up and downregulated in subpopulations of CD133+Lin-CD45^+^ HSPCs dataset (Figures 6E,F) revealed high expression of C1QA, CLEC10A, GPRC5A, SASH1, KRBOX1, RNF150, PRSS51, HSPA12A, PDE10A, MMP16, AC015908.2, and ARHGAP20 involved in ubiquitin-dependent protein catabolic processes, proteolysis, ATP binding activity, signal transduction due to regulating the availability of intracellular cyclic nucleotides, and NLRP-dependent pyroptosis. Reactome and GO analysis of the top 50 upregulated genes for PROM1+CD34^+^ clusters in CD133+Lin-CD45^+^ HSPCs dataset identified mRNA encoding RAC1 and CDC42 GTPase, genes involved in metabolism, hormone receptors, and lipoproteins remodeling (Figure 6E). The top 50 downregulated genes in a primitive subpopulation of PROM1+CD34^+^ cells in the CD133+Lin-CD45^+^ HSPCs dataset were associated with differentiation, TLRs signaling, antigen processing and presentation, neutrophil degranulation, and cytokine-related immune responses (Figure 6F).

### Integrated analysis of both datasets

Finally, we performed an integrated analysis of CD34^+^Lin^−^CD45^+^ and CD133^+^Lin^−^CD45^+^ HSPCs datasets merged and treated as “pseudobulk”. We compared their pseudobulk profiles and observed that CD34^+^ and CD133^+^ HSPCs do not differ significantly in gene expression, as evidenced by the strong positive linear relationship between the cell populations (R = 0.99). We observed several genes expressed at similar levels in both cell populations (Supplementary Figure S3). Therefore, next, we visualized clusters using uniform manifold approximation and d projection (UMAP) implemented in Seurat (version 5.0.1) (Satija et al., 2015; Hao et al., 2024). Initially, the analysis was done with the datasets without integration. Visual inspection of the result confirmed that clusters are defined by the cell population type. The UMAP plot illustrates clustering of cells, with CD34^+^Lin^−^CD45^+^ (red) and CD133^+^Lin^−^CD45^+^ (blue) forming distinct but partially overlapping populations, indicating transcriptional similarities (Supplementary Figure S3). However, the gene expression profiles for both populations overlap, and we do not observe unique subpopulations within CD34^+^Lin^−^CD45^+^ and CD133^+^Lin^−^CD45^+^ HSPCs (Supplementary Figures S3A,C). This has been confirmed after integration because cells from both cell populations cluster together (Supplementary Figures S3A,B). Additionally, when we compared CD34^+^Lin^−^CD45^+^ and CD133^+^Lin^−^CD45^+^ HSPCs side-by-side, we did not see differences in the distribution of subpopulations (Supplementary Figure S3C).

Finally, we analyzed in integrated datasets the expression of cell marker genes to highlight the differences in the expression of primitive hematopoietic stem cell markers: CD34, PROM1, PECAM1 (CD31), c-KIT (CD117); lymphocyte markers: CD2, CD4; monocytes and macrophages markers: CD14; erythrocyte marker: GYPA and megakaryocytes marker: ITGA2B. We also checked the expression of PTPRC, a marker common for leukocytes, present at the surfaces of all immune system cells except for erythrocytes and platelets. We observed that there are two main cluster clouds in both cell populations. While CD34, PROM1, and c-KIT expression characterize the first, the second one expresses CD4 and CD14. It is consistent with the results from non-integrated analysis. The aggregation analysis of both datasets shows high similarity by identifying 23 cell clusters in each one that align together (Supplementary Figures S3A–C). The very high correlation (R = 0.99) between the transcriptomes of CD34^+^ and CD133+ HSPCs in our pseudobulk analysis highlights a strong transcriptional overlap between these two commonly used markers. This finding supports the notion that CD34 and CD133 mark substantially overlapping populations of primitive hematopoietic progenitors. While both subsets exhibit similar expression of genes associated with stemness and early differentiation, our clustering analysis reveals functionally relevant substructures with differential gene expression, particularly in genes related to signaling, lineage priming, and immune regulation. Importantly, the observed correlation validates the use of either CD34 or CD133 as viable purification targets for the enrichment of multipotent HSPCs in research and clinical settings. At the same time, the subtle transcriptomic differences underscore the potential benefit of dual-marker strategies (CD34^+^CD133+) for capturing the most primitive, quiescent subpopulations.

There are several different strategies to purify and identify HSPCs (Bhatia et al., 1997; Kato and Radbruch, 1993; Bujko et al., 2019). Hematopoietic stem cells (HSCs) and multipotent hematopoietic progenitors are routinely isolated using various markers. The most common is FACS or immunomagnetic sorting using HSPCs expressed markers. Additionally, HSPCs could be purified based on minimal accumulation of metabolic fluorochromes such as pyronin Y (mRNA marker), Rh123 (efflux pump substrate), and Hoe33342 (DNA marker). It has been proposed that applying SLAM family markers, CD150, CD48, CD229, and CD244, can distinguish HSCs and more restricted progenitors. In this paper, we employed FACS to sort two populations of HSPCs based on the expression of CD34, CD133, and CD45 antigens and the lack of expression of lineage differentiation markers. The first one, the CD34 antigen, is a member of a family of single-pass transmembrane sialomucin proteins expressed on human UCB and bone marrow lymphohematopoietic stem cells, progenitor cells, endothelial cells, mast cells, and a subpopulation of dendritic cells (Kato and Radbruch, 1993). The other one, CD133 antigen (PROM1 - prominin-1), is a member of pentaspan transmembrane glycoproteins, which specifically localize to cellular protrusions expressed on HSPCs, endothelial progenitors, embryonic stem cells, some types of neural cells and is associated as a marker with several tumors (Yin et al., 1997; Weigmann et al., 1997; Katoh and Katoh, 2007; Rappa et al., 2008). As reported, human HSPCs co-express CD34 and CD133 antigens, and 30%–60% of CD34^+^ cells co-express CD133 antigens.

We performed single-cell RNA-sequencing (RNA-seq) analysis of the transcriptome between UCB-derived CD34^+^Lin-CD45^+^ and CD133^+^Lin-CD45^+^ cells enriched for HSPCs and report similarities and differences in transcriptome between these cells at single cell level and, described mRNA clusters detected by RNA-seq. We detected some differences in transcriptome between subpopulations of these cells identified by scRNA-seq visualized as clusters using uniform manifold approximation and d projection (UMAP). Nevertheless, integrated analysis of CD34^+^Lin^−^CD45^+^ and CD133^+^Lin^−^CD45^+^ datasets, which were merged and treated as “pseudobulk” revealed that both populations do not differ significantly in gene expression, as evidenced by the very strong positive linear relationship between these cells. Our results show that the expression profiles for both populations overlap, and we do not observe unique subpopulations within CD34^+^Lin^−^CD45^+^ and CD133^+^Lin^−^CD45^+^ HSPCs.

To achieve reliable results that enable biologically meaningful conclusions, every stage of the scRNA-seq experiment must be meticulously executed, including cell sorting, single-cell library preparation, quality control, and data analysis. The decision to work with sorted material, rather than the conventional approach of using a full pellet of blood cells, proved to be valid and demonstrated that HSPC analysis is feasible even with a limited cell number.

## Data Availability

The datasets presented in this study can be found in online repositories. The names of the repository/repositories and accession number(s) can be found below: https://www.ncbi.nlm.nih.gov/sra/PRJNA1128409.

## Supplementary material

SUPPLEMENTARY FIGURE S1 Cell sorting strategy for the isolation of HSPCs from hUCB. Immunostained cells were first visualized by dot plot showing forward scatter (FSC) vs. side scatter (SSC) signals, where small events ranging from 2 to 15 µm were gated (P1) **(A)** and further analyzed for the expression of Lineage markers. Lineage negative events were gated (Lin-) **(B)** and analyzed for the expression of CD45 and CD34 **(C)** or CD133 **(D)** antigens. The populations of CD34^+^Lin^−^CD45^+^ HSPCs **(C)** and CD133^+^Lin^−^CD45^+^ HSPCs **(D)** were separately sorted on the MoFlo Astrios EQ cell sorter. MNCs isolation and staining were described in the Materials and Methods section. Representative dot plots saved during the sample acquisition are shown.

SUPPLEMENTARY FIGURE S2 Quality control parameters of final libraries. Left panel: Electropherograms of final libraries: CD133+lin-CD45^+^ **(A)** and CD34+lin-CD45^+^ **(B)** prepared with the use of Chromium Next GEM Single Cell 3′ GEM, Library & Gel Bead Kit v3.1, and Single Index Kit T Set A (10X Genomics, United States), assessed with the D5000 ScreenTape assay. DNA shows a peak size of around 400 bp, which matches the expected size range between 400 and 600 bp. Right panel: FastQC reports showing per base sequences quality in all samples: CD133+lin-CD45^+^ **(C)** and CD34+lin-CD45^+^ **(D)**.

SUPPLEMENTARY FIGURE S3 Integration analysis of HSPC libraries. Integrating two scRNA Seq datasets (CD34^+^Lin^−^CD45^−^ and CD133^+^Lin^−^CD45^−^) visualized the lack of differences between two subtypes of HSPCs **(A)** and identified 23 common cell clusters within both datasets **(B,C)**. Slight differences result from the different number of cells obtained for both libraries **(C)**.
