# Supplementary material for: Optimizing single cell RNA sequencing of stem cells. A streamlined workflow for enhanced sensitivity and reproducibility in hematopoietic studies. The use of human umbilical cord blood-derived hematopoietic stem and progenitor cells
Source: Front Cell Dev Biol. 2025 May 15;13:1590889. doi: 10.3389/fcell.2025.1590889 (PMC12119605; doi:10.3389/fcell.2025.1590889)
Supplement: Supplementary file 1 [file Table1.docx]

**Supplementary tables:**

**Table S1.** Top 50 up and down-regulated genes in cluster 1 in CD34+Lin-CD45+ cells.

| **gene** | **avg_log2FC** | **pct.1** | **pct.2** | **p_val_adj** | **expression** |
| --- | --- | --- | --- | --- | --- |
| NKAIN2 | 2.27398046459357 | 0.991 | 0.319 | 0 | UP-regulation |
| NRIP1 | 1.86245602397462 | 0.988 | 0.455 | 2.0318939479687E-284 | UP-regulation |
| INPP4B | 1.78558916684331 | 0.933 | 0.282 | 1.43408606848523E-278 | UP-regulation |
| CALN1 | 1.74019100800356 | 0.863 | 0.198 | 1.89285442685098E-289 | UP-regulation |
| LRBA | 1.70690616477306 | 0.96 | 0.453 | 7.41203838576898E-227 | UP-regulation |
| CASC15 | 1.6385399983526 | 0.909 | 0.276 | 4.63292714064093E-258 | UP-regulation |
| RNF220 | 1.54932063715474 | 0.846 | 0.246 | 5.32512464040277E-233 | UP-regulation |
| CD109 | 1.54627876468274 | 0.77 | 0.181 | 3.29838596824546E-240 | UP-regulation |
| MSRB3 | 1.54374146468056 | 0.826 | 0.188 | 4.60963737590471E-276 | UP-regulation |
| DAPK1 | 1.51665480966827 | 0.849 | 0.252 | 2.86833150835522E-230 | UP-regulation |
| CHRM3 | 1.51190602494602 | 0.778 | 0.182 | 5.9857507780448E-242 | UP-regulation |
| ERG | 1.50977870130442 | 0.817 | 0.193 | 3.59828966044697E-253 | UP-regulation |
| ELMO1 | 1.49013667905622 | 0.977 | 0.621 | 2.27246580224489E-186 | UP-regulation |
| AL589693.1 | 1.48163219899133 | 0.689 | 0.245 | 5.88703293983254E-130 | UP-regulation |
| CRHBP | 1.47254889451161 | 0.784 | 0.196 | 6.70590010378007E-228 | UP-regulation |
| SPTBN1 | 1.46659524109296 | 0.88 | 0.288 | 5.8149729605164E-219 | UP-regulation |
| SSBP2 | 1.4384478153094 | 0.954 | 0.443 | 1.03401777033937E-193 | UP-regulation |
| MSI2 | 1.42400258067031 | 0.977 | 0.407 | 1.2398969001062E-224 | UP-regulation |
| ST8SIA6 | 1.38739848962413 | 0.734 | 0.173 | 2.28573328065826E-218 | UP-regulation |
| TCF4 | 1.35109548364481 | 0.799 | 0.228 | 9.42642002754672E-203 | UP-regulation |
| IGF2BP2 | 1.34476209767233 | 0.833 | 0.249 | 8.59882957299041E-215 | UP-regulation |
| SGIP1 | 1.32419015275399 | 0.562 | 0.088 | 5.7534488326018E-220 | UP-regulation |
| HMGA2 | 1.32333714488749 | 0.644 | 0.122 | 3.30951961844877E-221 | UP-regulation |
| GNAI1 | 1.31536368370826 | 0.689 | 0.163 | 6.92446613469782E-199 | UP-regulation |
| RERE | 1.31400136551874 | 0.952 | 0.438 | 1.10705313312573E-199 | UP-regulation |
| CABLES1 | 1.30991002434773 | 0.578 | 0.114 | 7.83441871964638E-189 | UP-regulation |
| DACH1 | 1.29236217562025 | 0.707 | 0.163 | 8.02323184470922E-207 | UP-regulation |
| AJ009632.2 | 1.27968189991172 | 0.58 | 0.11 | 5.81274956273641E-195 | UP-regulation |
| SLC39A8 | 1.27903740703031 | 0.647 | 0.149 | 8.38184838718131E-188 | UP-regulation |
| RBPMS | 1.27557194950857 | 0.672 | 0.152 | 4.04986111578838E-197 | UP-regulation |
| PTPRD | 1.2733348874441 | 0.598 | 0.113 | 2.56047038008522E-202 | UP-regulation |
| COL24A1 | 1.26608485016853 | 0.657 | 0.138 | 5.18284129992049E-203 | UP-regulation |
| MECOM | 1.25052667909973 | 0.438 | 0.06 | 2.13998472453128E-183 | UP-regulation |
| MEIS1 | 1.24647671247946 | 0.584 | 0.108 | 1.37922294795915E-199 | UP-regulation |
| PPP1R16B | 1.24376360510897 | 0.809 | 0.245 | 2.37800024927267E-182 | UP-regulation |
| ANKRD28 | 1.24007489559024 | 0.898 | 0.382 | 3.83325218220959E-165 | UP-regulation |
| MIR99AHG | 1.23481544437042 | 0.526 | 0.084 | 2.54109166606038E-200 | UP-regulation |
| ANGPT1 | 1.20608802905474 | 0.6 | 0.143 | 2.30669840511227E-164 | UP-regulation |
| ITGA9 | 1.19714615901723 | 0.605 | 0.133 | 1.15019714076219E-178 | UP-regulation |
| PBX1 | 1.19591074238905 | 0.518 | 0.09 | 8.21464163510086E-181 | UP-regulation |
| TOX | 1.19469401715833 | 0.759 | 0.209 | 1.07645662510933E-179 | UP-regulation |
| ZNF521 | 1.19116990247073 | 0.708 | 0.189 | 6.19073121725957E-174 | UP-regulation |
| TAFA2 | 1.15587813026274 | 0.482 | 0.117 | 9.51172299346934E-123 | UP-regulation |
| CRIM1 | 1.15454527369836 | 0.541 | 0.105 | 3.55845388712106E-172 | UP-regulation |
| IMMP2L | 1.14881833373764 | 0.758 | 0.255 | 2.87311204024969E-144 | UP-regulation |
| MLLT3 | 1.12471972695579 | 0.75 | 0.227 | 3.30914985490713E-164 | UP-regulation |
| PDZD2 | 1.12093755357775 | 0.538 | 0.117 | 2.14225254020877E-154 | UP-regulation |
| INPP5D | 1.11641295592361 | 0.851 | 0.332 | 7.76627255934306E-152 | UP-regulation |
| KIAA1211 | 1.10358041359726 | 0.505 | 0.102 | 1.01500574667637E-151 | UP-regulation |
| PTPRM | 1.10341156496257 | 0.443 | 0.061 | 4.23619344190134E-182 | UP-regulation |
| S100A9 | -5.36817163040099 | 0.153 | 0.481 | 5.54931610304088E-76 | DOWN-regulation |
| S100A8 | -4.25838399131937 | 0.055 | 0.413 | 1.37578581026464E-77 | DOWN-regulation |
| LYZ | -3.4534184032355 | 0.06 | 0.402 | 2.4571019414409E-73 | DOWN-regulation |
| VCAN | -3.2118771519503 | 0.011 | 0.345 | 2.20248115830042E-70 | DOWN-regulation |
| TYROBP | -3.03350934592773 | 0.048 | 0.424 | 8.30378794202186E-84 | DOWN-regulation |
| NAMPT | -3.02523311820402 | 0.349 | 0.52 | 1.19867587440825E-46 | DOWN-regulation |
| SAT1 | -2.91539184886494 | 0.315 | 0.527 | 1.07081953024382E-55 | DOWN-regulation |
| CXCL8 | -2.8237117359001 | 0.09 | 0.343 | 7.30291364862845E-46 | DOWN-regulation |
| S100A6 | -2.79633226783208 | 0.278 | 0.522 | 6.30854909477263E-63 | DOWN-regulation |
| G0S2 | -2.76719598304831 | 0.017 | 0.307 | 4.66461397244538E-57 | DOWN-regulation |
| FCN1 | -2.66047463179212 | 0.015 | 0.355 | 6.30628994531005E-72 | DOWN-regulation |
| CST3 | -2.61265191714893 | 0.222 | 0.451 | 5.9460057873034E-49 | DOWN-regulation |
| CTSS | -2.51939544116597 | 0.309 | 0.497 | 4.77314011368712E-46 | DOWN-regulation |
| PLAUR | -2.47637818844771 | 0.087 | 0.36 | 2.43866551425836E-52 | DOWN-regulation |
| ANXA1 | -2.44747478413897 | 0.08 | 0.433 | 6.96353912973009E-77 | DOWN-regulation |
| CRIP1 | -2.39727454278992 | 0.052 | 0.395 | 2.15317343929319E-72 | DOWN-regulation |
| MNDA | -2.30692858490642 | 0.012 | 0.326 | 2.67808574140897E-64 | DOWN-regulation |
| LGALS1 | -2.2754029607035 | 0.055 | 0.367 | 9.22536373137965E-63 | DOWN-regulation |
| S100A12 | -2.2707643532437 | 0.012 | 0.26 | 2.28939247277949E-46 | DOWN-regulation |
| S100A10 | -2.179692975194 | 0.116 | 0.425 | 1.61882978058297E-63 | DOWN-regulation |
| S100A11 | -2.17720730867915 | 0.171 | 0.42 | 1.18039752343739E-48 | DOWN-regulation |
| LGALS2 | -2.12337631303992 | 0.007 | 0.271 | 2.86931102384996E-51 | DOWN-regulation |
| IFI30 | -2.11305016837149 | 0.015 | 0.304 | 2.22966497578329E-57 | DOWN-regulation |
| S100A4 | -2.08616135476317 | 0.655 | 0.694 | 5.38919169347623E-54 | DOWN-regulation |
| FCER1G | -1.99376779136858 | 0.062 | 0.355 | 1.70139134420414E-57 | DOWN-regulation |
| TMSB4X | -1.9833842400989 | 0.952 | 0.932 | 2.11007139757747E-150 | DOWN-regulation |
| COTL1 | -1.95536792383586 | 0.066 | 0.374 | 2.95637835759605E-62 | DOWN-regulation |
| THBS1 | -1.93464994614307 | 0.008 | 0.232 | 7.51960764899321E-41 | DOWN-regulation |
| SLC8A1 | -1.87505740514305 | 0.114 | 0.321 | 1.33981241826486E-33 | DOWN-regulation |
| IL1B | -1.81996951523435 | 0.088 | 0.281 | 9.55614536291853E-30 | DOWN-regulation |
| FGD4 | -1.81203959454016 | 0.174 | 0.341 | 8.35119022464458E-26 | DOWN-regulation |
| SLC11A1 | -1.80349603886053 | 0.023 | 0.27 | 1.0760638620615E-45 | DOWN-regulation |
| ARHGAP26 | -1.80086391415665 | 0.444 | 0.496 | 1.12521962745236E-20 | DOWN-regulation |
| RAB31 | -1.77539982342396 | 0.047 | 0.288 | 7.56967651437576E-43 | DOWN-regulation |
| RBM47 | -1.67494055335602 | 0.091 | 0.289 | 9.57682312815183E-31 | DOWN-regulation |
| FTL | -1.66715904981905 | 0.979 | 0.958 | 3.78550574180538E-87 | DOWN-regulation |
| ACSL1 | -1.65428638454171 | 0.202 | 0.346 | 2.09695328435596E-21 | DOWN-regulation |
| SRGN | -1.632171615152 | 0.581 | 0.638 | 9.48680182384984E-36 | DOWN-regulation |
| KYNU | -1.62566091630566 | 0.004 | 0.232 | 3.517276104016E-42 | DOWN-regulation |
| FYB1 | -1.6247947224312 | 0.055 | 0.3 | 8.86267578841448E-44 | DOWN-regulation |
| ATP13A3 | -1.62010839920092 | 0.141 | 0.322 | 1.45821137807438E-27 | DOWN-regulation |
| TNFAIP3 | -1.58837697506689 | 0.141 | 0.361 | 3.12058272983532E-36 | DOWN-regulation |
| FTH1 | -1.58658460512716 | 0.988 | 0.961 | 1.55040234830438E-71 | DOWN-regulation |
| CLEC7A | -1.57782047111665 | 0.013 | 0.251 | 9.38004570838614E-44 | DOWN-regulation |
| TMSB10 | -1.57086049395778 | 0.877 | 0.872 | 6.3911534663882E-119 | DOWN-regulation |
| ACTB | -1.56417135796824 | 0.902 | 0.91 | 2.38587408202904E-143 | DOWN-regulation |
| C5AR1 | -1.5632729840373 | 0.007 | 0.231 | 4.783706072954E-41 | DOWN-regulation |
| HLA-B | -1.55942129876835 | 0.68 | 0.753 | 8.64574241768523E-82 | DOWN-regulation |
| CFD | -1.54928974038218 | 0.02 | 0.251 | 1.1206991565297E-41 | DOWN-regulation |
| NFKBIA | -1.54004007369887 | 0.446 | 0.557 | 2.69788625147067E-31 | DOWN-regulation |

**Table S2.** Top 50 up and down-regulated genes in cluster 5 in CD34+Lin-CD45+ cells.

| **gene** | **avg_log2FC** | **pct.1** | **pct.2** | **p_val_adj** | **expression** |
| --- | --- | --- | --- | --- | --- |
| IL7R | 3.49643388798835 | 0.845 | 0.025 | 0 | UP-regulation |
| CAMK4 | 3.4513717887145 | 0.808 | 0.012 | 0 | UP-regulation |
| BCL11B | 3.28462589801549 | 0.797 | 0.018 | 0 | UP-regulation |
| LEF1 | 2.6799158990817 | 0.648 | 0.016 | 0 | UP-regulation |
| LTB | 2.62337034732133 | 0.907 | 0.2 | 4.94733807596699E-237 | UP-regulation |
| CD3D | 2.5294809060563 | 0.673 | 0.014 | 0 | UP-regulation |
| ETS1 | 2.47353501193881 | 0.738 | 0.077 | 2.27006483862858E-303 | UP-regulation |
| IL32 | 2.43145233038616 | 0.608 | 0.017 | 0 | UP-regulation |
| BACH2 | 2.41012648841562 | 0.859 | 0.222 | 4.01178887765393E-200 | UP-regulation |
| PLCL1 | 2.22260031184095 | 0.515 | 0.045 | 1.69917899260334E-223 | UP-regulation |
| CD3G | 2.21203055658588 | 0.597 | 0.016 | 0 | UP-regulation |
| CD96 | 2.12218318753545 | 0.51 | 0.029 | 1.50532133632574E-271 | UP-regulation |
| TCF7 | 2.10989237219153 | 0.606 | 0.075 | 5.38033207048748E-218 | UP-regulation |
| TRBC2 | 2.10234867180549 | 0.594 | 0.142 | 4.11155818640991E-123 | UP-regulation |
| CD3E | 2.08326390142629 | 0.524 | 0.015 | 0 | UP-regulation |
| THEMIS | 2.04920820930674 | 0.437 | 0.006 | 0 | UP-regulation |
| NELL2 | 2.0478983501716 | 0.315 | 0.004 | 1.97167058039463E-266 | UP-regulation |
| PCED1B-AS1 | 2.01431483434297 | 0.527 | 0.033 | 3.69199234457866E-270 | UP-regulation |
| KLF2 | 1.97394279305147 | 0.749 | 0.197 | 6.03939973454291E-147 | UP-regulation |
| TRAC | 1.96397355233659 | 0.451 | 0.01 | 0 | UP-regulation |
| TRBC1 | 1.963732174508 | 0.397 | 0.024 | 1.71849529730368E-203 | UP-regulation |
| SKAP1 | 1.94582776234497 | 0.741 | 0.214 | 2.28485224305808E-132 | UP-regulation |
| ARL4C | 1.90256653854555 | 0.654 | 0.136 | 5.3935260317873E-147 | UP-regulation |
| PRKCA | 1.89625803555034 | 0.552 | 0.085 | 1.11546535264645E-157 | UP-regulation |
| LCK | 1.82005339004448 | 0.47 | 0.025 | 4.63289689366815E-262 | UP-regulation |
| SERINC5 | 1.81299909116207 | 0.524 | 0.12 | 2.82315452761817E-104 | UP-regulation |
| CD247 | 1.80538458437164 | 0.561 | 0.037 | 9.63169569501394E-269 | UP-regulation |
| PDE3B | 1.80444502579041 | 0.715 | 0.313 | 5.58859538436599E-82 | UP-regulation |
| SARAF | 1.77125326834646 | 0.885 | 0.515 | 1.55388730066299E-106 | UP-regulation |
| MAML2 | 1.76616517082796 | 0.685 | 0.269 | 4.83713163402291E-80 | UP-regulation |
| NOSIP | 1.73377142243998 | 0.561 | 0.143 | 6.53762489327087E-104 | UP-regulation |
| BCL2 | 1.72697681108059 | 0.473 | 0.147 | 1.58284589884472E-63 | UP-regulation |
| SPOCK2 | 1.64899749262726 | 0.42 | 0.054 | 3.63753804340588E-135 | UP-regulation |
| LEPROTL1 | 1.63455941493494 | 0.594 | 0.183 | 3.89672080598581E-92 | UP-regulation |
| NDFIP1 | 1.6200434873391 | 0.549 | 0.182 | 6.51381470471739E-75 | UP-regulation |
| ZC3HAV1 | 1.59916390665324 | 0.693 | 0.293 | 1.17249404993994E-79 | UP-regulation |
| RASA3 | 1.59506614690289 | 0.569 | 0.158 | 4.79294816955689E-92 | UP-regulation |
| LBH | 1.59120100019491 | 0.406 | 0.032 | 7.52333787053403E-180 | UP-regulation |
| CNOT6L | 1.56649298793406 | 0.775 | 0.371 | 8.55006315938783E-80 | UP-regulation |
| OXNAD1 | 1.5539486443251 | 0.431 | 0.071 | 1.40367540565205E-112 | UP-regulation |
| ANK3 | 1.54631310131316 | 0.341 | 0.079 | 2.15536945818533E-60 | UP-regulation |
| ITK | 1.51835437210533 | 0.358 | 0.01 | 2.16069327402461E-245 | UP-regulation |
| TC2N | 1.5132894962256 | 0.352 | 0.039 | 4.36649260923911E-122 | UP-regulation |
| AAK1 | 1.50694237145715 | 0.577 | 0.186 | 4.11048114831165E-79 | UP-regulation |
| EPB41 | 1.5030946936401 | 0.659 | 0.293 | 1.44880347105316E-66 | UP-regulation |
| TLE5 | 1.50214588655153 | 0.699 | 0.284 | 1.60904750263473E-80 | UP-regulation |
| APBA2 | 1.49863502289394 | 0.327 | 0.012 | 9.20682216498944E-206 | UP-regulation |
| TNFAIP3 | 1.49685477958166 | 0.758 | 0.292 | 3.09048420994273E-87 | UP-regulation |
| RCAN3 | 1.49011812825343 | 0.377 | 0.043 | 1.7913521395424E-130 | UP-regulation |
| TXNIP | 1.4881203956819 | 0.721 | 0.294 | 2.60875622819244E-79 | UP-regulation |
| S100A9 | -5.16653913588363 | 0.107 | 0.455 | 2.51300878808232E-36 | DOWN-regulation |
| S100A8 | -4.25772359376253 | 0.031 | 0.383 | 9.82382697743962E-36 | DOWN-regulation |
| LYZ | -3.38894088865132 | 0.011 | 0.376 | 1.83430199534536E-37 | DOWN-regulation |
| HLA-DRA | -3.152897899363 | 0.028 | 0.711 | 1.481019104964E-105 | DOWN-regulation |
| TYROBP | -3.08827568748774 | 0.006 | 0.394 | 1.65457374056588E-41 | DOWN-regulation |
| VCAN | -3.05069005387537 | 0.014 | 0.316 | 1.9363360665452E-28 | DOWN-regulation |
| AREG | -3.0420614917934 | 0.065 | 0.594 | 5.94034318784687E-70 | DOWN-regulation |
| CST3 | -3.0410269060381 | 0.025 | 0.446 | 1.41489465402422E-46 | DOWN-regulation |
| CXCL8 | -3.00845034953541 | 0.011 | 0.327 | 5.03510785218688E-30 | DOWN-regulation |
| NAMPT | -2.97693273426038 | 0.203 | 0.517 | 3.12772205875593E-32 | DOWN-regulation |
| NKAIN2 | -2.90973888445469 | 0.023 | 0.455 | 9.55018556559465E-49 | DOWN-regulation |
| ZEB2 | -2.75186331693648 | 0.011 | 0.561 | 5.34769520648406E-71 | DOWN-regulation |
| CTSS | -2.71741699563794 | 0.121 | 0.495 | 1.41281219677129E-39 | DOWN-regulation |
| SAT1 | -2.71706617466199 | 0.192 | 0.518 | 3.20383188068144E-32 | DOWN-regulation |
| G0S2 | -2.65242774244078 | 0.011 | 0.282 | 3.91034072703735E-24 | DOWN-regulation |
| HLA-DRB1 | -2.63201389782398 | 0.017 | 0.598 | 1.35119938655702E-78 | DOWN-regulation |
| FCN1 | -2.55598776124369 | 0.008 | 0.326 | 1.18607845572497E-30 | DOWN-regulation |
| LYN | -2.52859812926085 | 0.017 | 0.614 | 2.55543544732576E-81 | DOWN-regulation |
| CD74 | -2.51849524881455 | 0.155 | 0.726 | 1.57059756465386E-86 | DOWN-regulation |
| PLAUR | -2.46627529918408 | 0.034 | 0.341 | 1.70127512598948E-28 | DOWN-regulation |
| PLXDC2 | -2.42803290360239 | 0.008 | 0.447 | 8.64742457265582E-50 | DOWN-regulation |
| GAB2 | -2.41773235460281 | 0.054 | 0.583 | 9.14862338320472E-68 | DOWN-regulation |
| LRMDA | -2.38501632676827 | 0.028 | 0.571 | 4.05949932100745E-70 | DOWN-regulation |
| PLCB1 | -2.37028803349405 | 0.076 | 0.618 | 1.40405961916484E-69 | DOWN-regulation |
| S100A4 | -2.31193047671882 | 0.254 | 0.723 | 1.64360697913533E-59 | DOWN-regulation |
| MNDA | -2.25570310413766 | 0 | 0.3 | 2.14128854634692E-28 | DOWN-regulation |
| FGD4 | -2.2466921162339 | 0.017 | 0.339 | 1.04676829398154E-30 | DOWN-regulation |
| S100A11 | -2.23531698652238 | 0.065 | 0.406 | 1.44786330895303E-32 | DOWN-regulation |
| NEAT1 | -2.22413812468863 | 0.29 | 0.68 | 1.17967536594493E-48 | DOWN-regulation |
| GSTP1 | -2.21894607613513 | 0.155 | 0.735 | 2.97807464827194E-88 | DOWN-regulation |
| NRIP1 | -2.18557448340575 | 0.13 | 0.57 | 7.9222386975586E-52 | DOWN-regulation |
| S100A12 | -2.15680546009281 | 0.011 | 0.238 | 1.25547452852899E-18 | DOWN-regulation |
| SLC8A1 | -2.10277441256863 | 0.011 | 0.311 | 1.27902745660511E-27 | DOWN-regulation |
| FTL | -2.0839875670158 | 0.873 | 0.968 | 6.37109246751389E-88 | DOWN-regulation |
| IFI30 | -2.05167952700797 | 0.003 | 0.28 | 2.70371373662274E-25 | DOWN-regulation |
| LGALS2 | -2.04739135570537 | 0 | 0.249 | 8.02159167118164E-22 | DOWN-regulation |
| IL1B | -2.02430722697882 | 0.003 | 0.271 | 3.38347359013954E-24 | DOWN-regulation |
| MEF2C | -2.01408511480583 | 0.011 | 0.505 | 1.36419879529028E-59 | DOWN-regulation |
| S100A6 | -1.9920470625717 | 0.338 | 0.496 | 6.60169766045912E-13 | DOWN-regulation |
| RNF130 | -1.97062372579428 | 0.054 | 0.535 | 3.22501666025549E-55 | DOWN-regulation |
| LGALS1 | -1.96145418616585 | 0.059 | 0.339 | 1.07354116852567E-23 | DOWN-regulation |
| ARHGAP26 | -1.95448860512493 | 0.228 | 0.509 | 3.69206673396301E-25 | DOWN-regulation |
| ACSL1 | -1.94680626970814 | 0.051 | 0.346 | 5.73062945553784E-26 | DOWN-regulation |
| TSPO | -1.93752526623284 | 0.118 | 0.557 | 2.90226347737407E-48 | DOWN-regulation |
| FTH1 | -1.91134752933037 | 0.927 | 0.968 | 5.48016419509282E-68 | DOWN-regulation |
| ETV6 | -1.90556587772183 | 0.217 | 0.667 | 1.30083014118358E-59 | DOWN-regulation |
| SAMSN1 | -1.8902234595646 | 0.135 | 0.439 | 4.51259996196546E-25 | DOWN-regulation |
| HDAC9 | -1.8883115615682 | 0.008 | 0.385 | 5.05338010494554E-39 | DOWN-regulation |
| HLA-DPB1 | -1.87271036967134 | 0.017 | 0.43 | 1.21910375681884E-44 | DOWN-regulation |
| RBM47 | -1.87008488535686 | 0.008 | 0.278 | 7.23587860184686E-24 | DOWN-regulation |

**Table S3.** Top 50 up and down-regulated genes in cluster 6 in CD34+Lin-CD45+ cells.

| **gene** | **avg_log2FC** | **pct.1** | **pct.2** | **p_val_adj** | **expression** |
| --- | --- | --- | --- | --- | --- |
| TUBA1B | 1.71395778922941 | 0.782 | 0.356 | 1.90894941205642E-76 | UP-regulation |
| TUBB | 1.67789958659487 | 0.788 | 0.279 | 3.67604546823468E-107 | UP-regulation |
| STMN1 | 1.61338866026699 | 0.848 | 0.348 | 8.63105592016581E-101 | UP-regulation |
| HSPA8 | 1.60284369913509 | 0.876 | 0.423 | 1.00719006950533E-93 | UP-regulation |
| PRSS57 | 1.51354645354084 | 0.791 | 0.277 | 3.66280779618034E-102 | UP-regulation |
| GAPDH | 1.48080839182674 | 0.991 | 0.827 | 2.9961553650791E-121 | UP-regulation |
| SNHG29 | 1.46158869341948 | 0.991 | 0.715 | 2.12876631607748E-129 | UP-regulation |
| LDHB | 1.41054849855175 | 0.909 | 0.475 | 3.23837972304713E-94 | UP-regulation |
| HMGA1 | 1.40412431661911 | 0.679 | 0.23 | 8.07892780149938E-85 | UP-regulation |
| HSPB1 | 1.35448253056402 | 0.718 | 0.245 | 3.80380003165247E-87 | UP-regulation |
| EEF1A1 | 1.34194982419034 | 1 | 0.947 | 1.79482954203468E-161 | UP-regulation |
| C1QTNF4 | 1.29797523506911 | 0.724 | 0.262 | 1.00527639173737E-74 | UP-regulation |
| RPS5 | 1.29370029216042 | 0.997 | 0.844 | 6.07604186685001E-135 | UP-regulation |
| PPIA | 1.26571952126151 | 0.918 | 0.63 | 5.84374878589386E-81 | UP-regulation |
| MDK | 1.25125059453599 | 0.485 | 0.141 | 6.38756577661958E-61 | UP-regulation |
| FXYD5 | 1.25040152436024 | 0.897 | 0.514 | 5.62730521787488E-73 | UP-regulation |
| RPL10A | 1.2330613670622 | 0.994 | 0.838 | 6.69268432666843E-126 | UP-regulation |
| MIF | 1.2290107923484 | 0.936 | 0.582 | 1.5266382284765E-74 | UP-regulation |
| ENO1 | 1.20330132047524 | 0.921 | 0.569 | 9.39766018540649E-75 | UP-regulation |
| ATP5F1B | 1.19972265051661 | 0.718 | 0.314 | 3.15353246300189E-62 | UP-regulation |
| NME4 | 1.19120273269621 | 0.545 | 0.164 | 2.23115776013005E-68 | UP-regulation |
| NME2 | 1.18644460097427 | 0.958 | 0.667 | 3.90340955643724E-85 | UP-regulation |
| RPS4X | 1.1755494281158 | 1 | 0.938 | 4.90315198810456E-141 | UP-regulation |
| PRDX1 | 1.1730663327944 | 0.882 | 0.468 | 8.42435432553397E-66 | UP-regulation |
| RPS3 | 1.17219361877146 | 0.997 | 0.932 | 1.39255217399317E-141 | UP-regulation |
| NHP2 | 1.17162382747138 | 0.639 | 0.225 | 6.10672687079538E-67 | UP-regulation |
| EEF1G | 1.16913000176232 | 0.976 | 0.776 | 2.20419014327342E-102 | UP-regulation |
| RACK1 | 1.16180999803994 | 0.991 | 0.896 | 2.37734197144932E-125 | UP-regulation |
| RPSA | 1.15304807560043 | 0.988 | 0.817 | 9.19371019724012E-105 | UP-regulation |
| CLEC11A | 1.14992129925457 | 0.524 | 0.169 | 1.02136406812184E-58 | UP-regulation |
| EGFL7 | 1.1484293851665 | 0.676 | 0.235 | 6.04165090792017E-71 | UP-regulation |
| IMPDH2 | 1.14613705872226 | 0.618 | 0.202 | 6.08071202879444E-71 | UP-regulation |
| ARHGDIB | 1.1443059167219 | 0.955 | 0.697 | 2.95279860645262E-80 | UP-regulation |
| SPINK2 | 1.12936552806907 | 0.806 | 0.313 | 5.93114580334769E-72 | UP-regulation |
| HINT1 | 1.11729956894955 | 0.973 | 0.671 | 4.01878720912322E-81 | UP-regulation |
| RPL3 | 1.115661459627 | 0.997 | 0.897 | 9.46542610990256E-122 | UP-regulation |
| RPS6 | 1.11129787696755 | 0.997 | 0.889 | 1.44571398700201E-114 | UP-regulation |
| RPL29 | 1.09711991266144 | 1 | 0.931 | 8.15055118363561E-132 | UP-regulation |
| RPL8 | 1.08183889434451 | 0.994 | 0.917 | 2.87390390637698E-129 | UP-regulation |
| ACTG1 | 1.07766799026729 | 0.976 | 0.812 | 4.92018088491105E-75 | UP-regulation |
| RPS2 | 1.07424861838834 | 1 | 0.938 | 2.7282060841951E-131 | UP-regulation |
| RPL4 | 1.07205592656318 | 0.948 | 0.68 | 1.3138618053735E-71 | UP-regulation |
| RPL7A | 1.06991913255247 | 0.997 | 0.915 | 1.19742133427301E-132 | UP-regulation |
| EEF2 | 1.06806217876545 | 0.945 | 0.708 | 1.02484321394571E-70 | UP-regulation |
| RPS3A | 1.05545156847673 | 1 | 0.94 | 2.21825047563183E-134 | UP-regulation |
| RPL32 | 1.04739835289711 | 0.997 | 0.941 | 7.85789753702395E-130 | UP-regulation |
| RPL12 | 1.04545593271093 | 1 | 0.917 | 3.31250490751799E-118 | UP-regulation |
| PHB2 | 1.0449586661183 | 0.645 | 0.27 | 2.99319100156714E-49 | UP-regulation |
| NME1 | 1.04141770822968 | 0.503 | 0.175 | 7.23479251630388E-49 | UP-regulation |
| NPM1 | 1.03610444445997 | 0.973 | 0.672 | 3.79681760170807E-72 | UP-regulation |
| S100A9 | -5.26217939128396 | 0.118 | 0.453 | 1.02931799324125E-32 | DOWN-regulation |
| S100A8 | -4.0596785972116 | 0.055 | 0.379 | 2.29626632067214E-29 | DOWN-regulation |
| NAMPT | -3.44937242045535 | 0.139 | 0.52 | 1.1871476013677E-40 | DOWN-regulation |
| LYZ | -3.19258393412829 | 0.067 | 0.37 | 1.17519918118073E-26 | DOWN-regulation |
| VCAN | -2.98152352453106 | 0.018 | 0.314 | 2.30236068720512E-25 | DOWN-regulation |
| CXCL8 | -2.91579819326046 | 0.039 | 0.323 | 1.68487546935709E-23 | DOWN-regulation |
| SAT1 | -2.84535341170612 | 0.255 | 0.512 | 2.56145704517786E-25 | DOWN-regulation |
| TYROBP | -2.75903103144331 | 0.076 | 0.387 | 3.92635437862685E-28 | DOWN-regulation |
| ARHGAP26 | -2.73733648656134 | 0.085 | 0.518 | 6.54934229488537E-47 | DOWN-regulation |
| G0S2 | -2.72258532610621 | 0.003 | 0.281 | 1.79456088756969E-23 | DOWN-regulation |
| NEAT1 | -2.66663977174345 | 0.276 | 0.679 | 6.4815761791129E-57 | DOWN-regulation |
| PLAUR | -2.52723504299015 | 0.027 | 0.339 | 2.07035633710865E-27 | DOWN-regulation |
| ZSWIM6 | -2.45138930623286 | 0.103 | 0.567 | 9.27476319732073E-55 | DOWN-regulation |
| FCN1 | -2.44376357149346 | 0.012 | 0.324 | 3.25780309227432E-27 | DOWN-regulation |
| CTSS | -2.40940035396256 | 0.245 | 0.484 | 1.20532960244037E-21 | DOWN-regulation |
| S100A6 | -2.40226821089699 | 0.245 | 0.502 | 1.91287780528108E-24 | DOWN-regulation |
| SIK3 | -2.33452040793586 | 0.3 | 0.786 | 6.50242567897758E-83 | DOWN-regulation |
| CST3 | -2.27543979783575 | 0.218 | 0.43 | 1.05652872223284E-16 | DOWN-regulation |
| ATP2B1 | -2.26668040550646 | 0.209 | 0.538 | 2.10393646621971E-34 | DOWN-regulation |
| SIPA1L1 | -2.25015414483372 | 0.097 | 0.48 | 1.37741244241087E-38 | DOWN-regulation |
| FGD4 | -2.25005317709131 | 0.03 | 0.336 | 1.66571949125756E-26 | DOWN-regulation |
| SAMSN1 | -2.20916012007277 | 0.145 | 0.437 | 1.76798416274591E-25 | DOWN-regulation |
| S100A12 | -2.19289573375886 | 0.006 | 0.237 | 6.87548632499922E-18 | DOWN-regulation |
| SLC8A1 | -2.19145827784679 | 0.018 | 0.309 | 8.746599550412E-25 | DOWN-regulation |
| MNDA | -2.15532753488222 | 0.006 | 0.297 | 7.49545945797289E-25 | DOWN-regulation |
| DPYD | -2.14918872538516 | 0.285 | 0.689 | 1.11114576684596E-55 | DOWN-regulation |
| PLXDC2 | -2.11292082724377 | 0.088 | 0.439 | 2.36806451888725E-33 | DOWN-regulation |
| ZFAND3 | -2.10328516749944 | 0.23 | 0.712 | 2.58089266915343E-68 | DOWN-regulation |
| MALAT1 | -2.09794356482238 | 0.979 | 0.987 | 6.27367914636314E-136 | DOWN-regulation |
| LGALS2 | -2.03048314187697 | 0.003 | 0.247 | 1.67995307756449E-19 | DOWN-regulation |
| JARID2 | -2.02007585649778 | 0.221 | 0.565 | 8.08947922929046E-37 | DOWN-regulation |
| IFI30 | -2.00296095335697 | 0.015 | 0.277 | 2.47932984858719E-21 | DOWN-regulation |
| AOAH | -1.98575552637335 | 0.027 | 0.351 | 3.76989161731248E-29 | DOWN-regulation |
| RBM47 | -1.97366072079461 | 0 | 0.277 | 1.84859670062783E-23 | DOWN-regulation |
| QKI | -1.96977150490117 | 0.182 | 0.541 | 8.35721902833561E-37 | DOWN-regulation |
| CRIP1 | -1.96544316834403 | 0.1 | 0.36 | 4.71189437658755E-20 | DOWN-regulation |
| PTPRE | -1.95651379412612 | 0.076 | 0.421 | 2.75661403424149E-32 | DOWN-regulation |
| S100A11 | -1.94973150422566 | 0.197 | 0.395 | 9.18684493118045E-15 | DOWN-regulation |
| PICALM | -1.93778952130498 | 0.094 | 0.47 | 9.22639736030646E-37 | DOWN-regulation |
| SMCHD1 | -1.90766935981701 | 0.215 | 0.599 | 1.24933903186826E-42 | DOWN-regulation |
| RABGEF1 | -1.88216394212566 | 0.061 | 0.422 | 1.72360924475007E-33 | DOWN-regulation |
| ACSL1 | -1.86052727082 | 0.088 | 0.341 | 4.91087239336871E-19 | DOWN-regulation |
| RAB31 | -1.84793761296126 | 0.006 | 0.269 | 1.38435792908692E-21 | DOWN-regulation |
| ARIH1 | -1.84333959313761 | 0.215 | 0.65 | 5.2437715352969E-55 | DOWN-regulation |
| TET2 | -1.83114675083067 | 0.109 | 0.425 | 1.57453454078356E-27 | DOWN-regulation |
| NFKB1 | -1.82601382185895 | 0.191 | 0.504 | 8.92632494705949E-29 | DOWN-regulation |
| THBS1 | -1.81328080721308 | 0.012 | 0.211 | 4.15878847254087E-14 | DOWN-regulation |
| ATP13A3 | -1.80077435670909 | 0.052 | 0.312 | 3.78035341933875E-20 | DOWN-regulation |
| GAB2 | -1.79680553906569 | 0.197 | 0.57 | 4.94467514817765E-39 | DOWN-regulation |
| FYN | -1.78980093811317 | 0.121 | 0.469 | 1.95887359979122E-32 | DOWN-regulation |

**Table S4.** Top 50 up and down-regulated genes in cluster 7 in CD34+Lin-CD45+ cells.

| **gene** | **avg_log2FC** | **pct.1** | **pct.2** | **p_val_adj** | **expression** |
| --- | --- | --- | --- | --- | --- |
| NEGR1 | 2.705056608397 | 0.408 | 0.029 | 3.32057023881105E-153 | UP-expression |
| EBF1 | 2.51077932961043 | 0.353 | 0.02 | 3.1074817013722E-147 | UP-expression |
| LINC01374 | 2.44199463685964 | 0.486 | 0.005 | 0 | UP-expression |
| AL589693.1 | 2.36955668511718 | 0.784 | 0.292 | 1.30541462270782E-73 | UP-expression |
| LSAMP | 2.35019651998119 | 0.275 | 0.004 | 3.23582573980545E-202 | UP-expression |
| JCHAIN | 2.30380051494805 | 0.436 | 0.003 | 0 | UP-expression |
| LTB | 2.27060835498437 | 0.807 | 0.225 | 1.61199640384049E-97 | UP-expression |
| MME | 2.22301357298109 | 0.44 | 0.011 | 4.35450083274042E-281 | UP-expression |
| AFF3 | 2.22248204397033 | 0.872 | 0.354 | 1.36860797971843E-83 | UP-expression |
| CNTNAP2 | 2.02573276191789 | 0.165 | 0.02 | 9.70275768847659E-35 | UP-expression |
| SCN3A | 2.00979120532817 | 0.44 | 0.01 | 1.74748096518538E-297 | UP-expression |
| AUTS2 | 1.94144599266922 | 0.803 | 0.38 | 3.46578696722936E-54 | UP-expression |
| ACY3 | 1.91907141429379 | 0.362 | 0.013 | 8.1362761204468E-194 | UP-expression |
| SLC8A1-AS1 | 1.86531210558585 | 0.353 | 0.056 | 2.86767930304332E-63 | UP-expression |
| AFF1 | 1.83360523247256 | 0.72 | 0.28 | 3.18576608095357E-58 | UP-expression |
| CLNK | 1.81358195009048 | 0.376 | 0.012 | 1.18997334357378E-215 | UP-expression |
| HIVEP3 | 1.8098700210791 | 0.509 | 0.137 | 4.33406666504422E-55 | UP-expression |
| COBLL1 | 1.80208591986131 | 0.408 | 0.044 | 5.20807419951696E-108 | UP-expression |
| BCL11A | 1.79448450303924 | 0.725 | 0.241 | 6.20022055422865E-69 | UP-expression |
| SFMBT2 | 1.72412283404902 | 0.546 | 0.167 | 8.48098747000612E-49 | UP-expression |
| VPREB1 | 1.66991562411101 | 0.33 | 0.009 | 1.25495889297014E-203 | UP-expression |
| TCF4 | 1.66479341418037 | 0.72 | 0.297 | 2.40890467170188E-45 | UP-expression |
| IGLL1 | 1.63020723136525 | 0.307 | 0.091 | 2.3205956556837E-23 | UP-expression |
| CD99 | 1.617678576022 | 0.839 | 0.431 | 2.16548992795505E-57 | UP-expression |
| COL24A1 | 1.61478246732764 | 0.578 | 0.202 | 1.26814612202727E-43 | UP-expression |
| RERE | 1.60564835539123 | 0.853 | 0.501 | 3.71726727624836E-44 | UP-expression |
| DDIT4 | 1.57761237116852 | 0.647 | 0.242 | 2.26452495364452E-46 | UP-expression |
| LNCAROD | 1.56260807919313 | 0.413 | 0.098 | 1.07640786488726E-47 | UP-expression |
| IFNG-AS1 | 1.52980835174245 | 0.229 | 0.015 | 1.62613722648766E-84 | UP-expression |
| MZB1 | 1.52295101037278 | 0.606 | 0.154 | 4.378277343116E-70 | UP-expression |
| AC092691.1 | 1.51485595156747 | 0.211 | 0.002 | 1.56776474696525E-176 | UP-expression |
| MSI2 | 1.50642685067015 | 0.771 | 0.482 | 1.11633604327573E-31 | UP-expression |
| ARHGEF7 | 1.480421983216 | 0.468 | 0.143 | 6.09477896505682E-40 | UP-expression |
| FCHSD2 | 1.47032334303917 | 0.656 | 0.382 | 6.14232964762558E-21 | UP-expression |
| PPP1R16B | 1.4676146654131 | 0.706 | 0.315 | 2.22167874414535E-41 | UP-expression |
| XYLT1 | 1.45347565924741 | 0.596 | 0.283 | 9.73573090736381E-29 | UP-expression |
| RNASEH2B | 1.41361201126035 | 0.495 | 0.155 | 4.54362688757832E-42 | UP-expression |
| SETBP1 | 1.39714206803665 | 0.353 | 0.12 | 8.43462589236889E-23 | UP-expression |
| ANKRD11 | 1.39238481876168 | 0.89 | 0.592 | 3.06246892441344E-47 | UP-expression |
| CYFIP2 | 1.378178236773 | 0.601 | 0.166 | 1.62588680150722E-60 | UP-expression |
| STK32B | 1.36501635139423 | 0.298 | 0.062 | 3.28629468046643E-38 | UP-expression |
| ARHGAP25 | 1.31678472197651 | 0.541 | 0.185 | 1.56683547204629E-39 | UP-expression |
| CD79A | 1.314077270676 | 0.294 | 0.024 | 3.55967489090102E-92 | UP-expression |
| STRBP | 1.29080190392496 | 0.583 | 0.219 | 2.52703989629193E-37 | UP-expression |
| ADA | 1.28224323232384 | 0.427 | 0.096 | 2.22693186856708E-52 | UP-expression |
| CBX4 | 1.27432720634492 | 0.385 | 0.079 | 9.24459798594419E-50 | UP-expression |
| CYGB | 1.27109230909557 | 0.216 | 0.001 | 3.88558446400624E-201 | UP-expression |
| MAN1A1 | 1.25839065105657 | 0.601 | 0.335 | 3.79633710428138E-19 | UP-expression |
| IRF8 | 1.25468875122959 | 0.326 | 0.047 | 2.9455901277277E-60 | UP-expression |
| MEF2A | 1.25250407633376 | 0.638 | 0.283 | 1.32090988188568E-32 | UP-expression |
| S100A9 | -4.77524601342278 | 0.115 | 0.445 | 1.19231740442747E-19 | DOWN-regulation |
| S100A8 | -3.76484841780517 | 0.069 | 0.371 | 1.83363759039754E-16 | DOWN-regulation |
| LYZ | -3.17471983971688 | 0.023 | 0.364 | 6.25352592494597E-20 | DOWN-regulation |
| VCAN | -2.95970017323397 | 0.023 | 0.306 | 5.58655668447603E-15 | DOWN-regulation |
| NAMPT | -2.93906762684276 | 0.174 | 0.509 | 3.73568463410591E-21 | DOWN-regulation |
| S100A6 | -2.61020798864513 | 0.147 | 0.5 | 3.4527657202479E-22 | DOWN-regulation |
| SAT1 | -2.54608614132323 | 0.252 | 0.506 | 1.65145209214209E-14 | DOWN-regulation |
| G0S2 | -2.37766383860029 | 0.014 | 0.274 | 1.03229886237185E-12 | DOWN-regulation |
| CXCL8 | -2.34763201508622 | 0.046 | 0.316 | 1.22767060274897E-12 | DOWN-regulation |
| FCN1 | -2.30676397931521 | 0.028 | 0.315 | 5.13105038959952E-15 | DOWN-regulation |
| SRGN | -2.22281122120844 | 0.261 | 0.647 | 2.706966026897E-29 | DOWN-regulation |
| S100A10 | -2.16602999882498 | 0.064 | 0.392 | 1.27543196430791E-18 | DOWN-regulation |
| CST3 | -2.15421144390751 | 0.165 | 0.427 | 1.21354123682372E-12 | DOWN-regulation |
| S100A12 | -2.15016184320557 | 0.009 | 0.231 | 3.25617893641247E-10 | DOWN-regulation |
| FGD4 | -2.11477799666505 | 0.046 | 0.328 | 1.84314037202306E-14 | DOWN-regulation |
| SAMSN1 | -2.11151220382359 | 0.133 | 0.43 | 8.31398586054976E-16 | DOWN-regulation |
| PLAUR | -2.05889858292125 | 0.083 | 0.329 | 4.73570869243688E-11 | DOWN-regulation |
| MNDA | -2.01691135959295 | 0.018 | 0.29 | 1.27887041769902E-13 | DOWN-regulation |
| LGALS2 | -1.9917100359926 | 0.005 | 0.241 | 2.10170604663789E-11 | DOWN-regulation |
| S100A11 | -1.98983518419831 | 0.128 | 0.393 | 8.95599457725141E-13 | DOWN-regulation |
| IFI30 | -1.98017495915298 | 0.009 | 0.271 | 2.80011464972264E-13 | DOWN-regulation |
| ANXA1 | -1.94175454786274 | 0.133 | 0.39 | 2.54945117735105E-12 | DOWN-regulation |
| S100A4 | -1.90161664090709 | 0.454 | 0.699 | 2.17594791873352E-18 | DOWN-regulation |
| CTSS | -1.84366418521765 | 0.303 | 0.476 | 1.92160157124626E-07 | DOWN-regulation |
| FTH1 | -1.75350464850846 | 0.954 | 0.966 | 9.49669153838832E-35 | DOWN-regulation |
| TYROBP | -1.71029529367833 | 0.216 | 0.373 | 0.0000214693216502105 | DOWN-regulation |
| SLC11A1 | -1.68887726422515 | 0.014 | 0.242 | 1.54389341275427E-10 | DOWN-regulation |
| NFKBIA | -1.64583762121622 | 0.289 | 0.551 | 1.25665407427216E-14 | DOWN-regulation |
| AOAH | -1.61831324432304 | 0.092 | 0.341 | 2.93604801962611E-11 | DOWN-regulation |
| SIPA1L1 | -1.60046136694498 | 0.165 | 0.467 | 1.31782033224208E-15 | DOWN-regulation |
| IL1B | -1.58792162698336 | 0.023 | 0.262 | 1.34143802015046E-10 | DOWN-regulation |
| THBS1 | -1.58753054377231 | 0.018 | 0.206 | 3.46799618972917E-07 | DOWN-regulation |
| NEAT1 | -1.56636829840453 | 0.486 | 0.659 | 2.63375776333851E-10 | DOWN-regulation |
| COTL1 | -1.53319558697381 | 0.069 | 0.338 | 1.17774930593642E-12 | DOWN-regulation |
| ARHGAP26 | -1.53201934083827 | 0.362 | 0.494 | 0.0000746977313722169 | DOWN-regulation |
| RAB31 | -1.52624917773144 | 0.064 | 0.26 | 2.77456136957261E-07 | DOWN-regulation |
| FTL | -1.51998908896015 | 0.945 | 0.962 | 1.03902138383167E-21 | DOWN-regulation |
| CSF3R | -1.47681528901806 | 0.06 | 0.341 | 3.00541796599962E-13 | DOWN-regulation |
| ATP13A3 | -1.47192947095801 | 0.115 | 0.302 | 2.11592013524594E-06 | DOWN-regulation |
| TSPO | -1.47065588908147 | 0.257 | 0.537 | 1.60784619223739E-14 | DOWN-regulation |
| CLEC7A | -1.46531787160929 | 0.014 | 0.224 | 3.17804168659121E-09 | DOWN-regulation |
| PLXDC2 | -1.45395289562276 | 0.183 | 0.426 | 4.11301340282565E-10 | DOWN-regulation |
| BLVRB | -1.44581233265666 | 0.032 | 0.229 | 7.40613824199681E-08 | DOWN-regulation |
| LGALS1 | -1.43962991614297 | 0.101 | 0.329 | 1.73963999065589E-08 | DOWN-regulation |
| CD83 | -1.43583413033036 | 0.073 | 0.308 | 2.19106715931451E-09 | DOWN-regulation |
| C5AR1 | -1.39244413866478 | 0.014 | 0.205 | 9.38808947275173E-08 | DOWN-regulation |
| CFD | -1.38586213955829 | 0.018 | 0.225 | 1.14662891403495E-08 | DOWN-regulation |
| ATP2B1 | -1.38131638510716 | 0.394 | 0.521 | 0.000296375533254757 | DOWN-regulation |
| GPCPD1 | -1.37012225667066 | 0.183 | 0.369 | 1.11793715191925E-06 | DOWN-regulation |
| SKAP1 | -1.35590087283377 | 0.032 | 0.263 | 3.19836949113183E-10 | DOWN-regulation |

**Table S5.** Top 50 up and down-regulated genes in cluster 8 in CD34+Lin-CD45+ cells.

| **gene** | **avg_log2FC** | **pct.1** | **pct.2** | **p_val_adj** | **expression** |
| --- | --- | --- | --- | --- | --- |
| HSP90AB1 | 0.609492029610716 | 0.77 | 0.748 | 0.00686585844978915 | UP-regulation |
| MYO1F | -0.932815774028393 | 0.118 | 0.292 | 0.00185703057018347 | DOWN-regulation |
| ASAP1 | -0.884943686765574 | 0.187 | 0.357 | 0.0105640092468411 | DOWN-regulation |
| SNX29 | -0.797539943522572 | 0.112 | 0.296 | 0.000516979753182193 | DOWN-regulation |
| ANKRD44 | -0.757025224238272 | 0.193 | 0.448 | 1.25300376437265E-06 | DOWN-regulation |
| BTG1 | -0.724636979123245 | 0.305 | 0.599 | 9.56640312366449E-08 | DOWN-regulation |
| WIPF1 | -0.69581412588404 | 0.086 | 0.24 | 0.0179652268346889 | DOWN-regulation |
| TXNIP | -0.692023910683425 | 0.144 | 0.333 | 0.00147397330044255 | DOWN-regulation |
| PHACTR2 | -0.67191735483578 | 0.086 | 0.243 | 0.00930202055313849 | DOWN-regulation |
| AP1S2 | -0.654767218458122 | 0.107 | 0.29 | 0.00228578362395701 | DOWN-regulation |
| STMP1 | -0.634808723448781 | 0.096 | 0.277 | 0.00113394644520981 | DOWN-regulation |
| JUNB | -0.632561927655177 | 0.385 | 0.637 | 0.000220738008150279 | DOWN-regulation |
| CYTH1 | -0.627572556837903 | 0.16 | 0.411 | 7.07605656049565E-06 | DOWN-regulation |
| UBL5 | -0.612561013930007 | 0.166 | 0.383 | 0.000171604117212229 | DOWN-regulation |
| PTPRE | -0.609614673816889 | 0.23 | 0.404 | 0.0322299349478285 | DOWN-regulation |
| IFITM2 | -0.597305039270651 | 0.278 | 0.483 | 0.0470470627601191 | DOWN-regulation |
| TGFBR2 | -0.593676891675424 | 0.112 | 0.273 | 0.0239998883862214 | DOWN-regulation |
| TRPS1 | -0.59027664338868 | 0.182 | 0.359 | 0.0151399647240606 | DOWN-regulation |
| ZFAND5 | -0.571934721342138 | 0.059 | 0.211 | 0.013965457153389 | DOWN-regulation |
| DDIT4 | -0.567293313052344 | 0.086 | 0.268 | 0.00189142721312982 | DOWN-regulation |
| KLF6 | -0.553787502004859 | 0.241 | 0.453 | 0.00251870110695313 | DOWN-regulation |
| HLA-A | -0.548399816190347 | 0.332 | 0.663 | 1.24922150534026E-07 | DOWN-regulation |
| ITM2B | -0.546566278241829 | 0.289 | 0.584 | 1.18738935259346E-06 | DOWN-regulation |
| MYL12B | -0.545244156851612 | 0.16 | 0.422 | 3.63348096767261E-06 | DOWN-regulation |
| ARPC4 | -0.54039919225328 | 0.086 | 0.237 | 0.0419607684953003 | DOWN-regulation |
| PRELID1 | -0.528586201883425 | 0.219 | 0.431 | 0.00361458999659276 | DOWN-regulation |
| AGO2 | -0.527287239534723 | 0.075 | 0.226 | 0.0261430602740299 | DOWN-regulation |
| TXNRD1 | -0.519165058246735 | 0.123 | 0.295 | 0.0148867407206008 | DOWN-regulation |
| TOP1 | -0.517608786184803 | 0.187 | 0.439 | 0.0000208048115608466 | DOWN-regulation |
| RNF125 | -0.49022495781779 | 0.15 | 0.328 | 0.0119011349045597 | DOWN-regulation |
| ATP1B3 | -0.486597007962483 | 0.353 | 0.592 | 0.0210929054621861 | DOWN-regulation |
| RAB7A | -0.480932824731779 | 0.235 | 0.494 | 0.0000975780528686335 | DOWN-regulation |
| LRRFIP1 | -0.472986412760298 | 0.326 | 0.617 | 0.0000292193345571424 | DOWN-regulation |
| ARPC5 | -0.468957636609393 | 0.235 | 0.424 | 0.0280669199301955 | DOWN-regulation |
| MAX | -0.466549251353641 | 0.07 | 0.252 | 0.000943006790995373 | DOWN-regulation |
| SH3KBP1 | -0.451785003042969 | 0.193 | 0.402 | 0.00378438548413388 | DOWN-regulation |
| NSA2 | -0.44617574555861 | 0.176 | 0.402 | 0.000227510575093665 | DOWN-regulation |
| LPP | -0.446171888768993 | 0.203 | 0.426 | 0.00105412917875609 | DOWN-regulation |
| HCST | -0.446157589198206 | 0.118 | 0.286 | 0.0542138458716012 | DOWN-regulation |
| CALM1 | -0.437970509502521 | 0.283 | 0.55 | 0.000256589386658269 | DOWN-regulation |
| RSRC2 | -0.42989440367356 | 0.139 | 0.329 | 0.00422644859978244 | DOWN-regulation |
| PRRC2C | -0.4241325295812 | 0.262 | 0.545 | 1.62400092202745E-06 | DOWN-regulation |
| SRSF10 | -0.422680725113362 | 0.134 | 0.333 | 0.000990925979661715 | DOWN-regulation |
| MBP | -0.421398953171676 | 0.198 | 0.389 | 0.043895778832 | DOWN-regulation |
| TSTD1 | -0.416874283021156 | 0.096 | 0.253 | 0.0417532385302942 | DOWN-regulation |
| GRB2 | -0.414238456401855 | 0.203 | 0.417 | 0.0119398374196771 | DOWN-regulation |
| BOD1L1 | -0.413561504234297 | 0.112 | 0.296 | 0.00951400889600116 | DOWN-regulation |
| CEP350 | -0.411975195773529 | 0.107 | 0.268 | 0.025496267739152 | DOWN-regulation |
| COP1 | -0.409707900228186 | 0.267 | 0.522 | 0.00204808870225084 | DOWN-regulation |
| SRSF5 | -0.394460592836356 | 0.326 | 0.617 | 0.000021220613775277 | DOWN-regulation |
| MBNL1 | -0.387908454097329 | 0.422 | 0.74 | 0.000104196895951633 | DOWN-regulation |

**Table S6.** Top 50 up and down-regulated genes in cluster 9 in CD34+Lin-CD45+ cells.

| **gene** | **avg_log2FC** | **pct.1** | **pct.2** | **p_val_adj** | **expression** |
| --- | --- | --- | --- | --- | --- |
| SLC24A3 | 1.91964285761915 | 0.431 | 0.033 | 2.04078673341525E-128 | UP-regulation |
| XACT | 1.88663622069905 | 0.575 | 0.083 | 3.74197599483959E-101 | UP-regulation |
| SOX4 | 1.59324751196053 | 0.823 | 0.366 | 1.72244390975652E-48 | UP-regulation |
| CNRIP1 | 1.59061075938553 | 0.448 | 0.011 | 2.13481239003127E-268 | UP-regulation |
| GATA2 | 1.54072108265161 | 0.635 | 0.122 | 2.65851150281974E-85 | UP-regulation |
| HPGDS | 1.44032019177217 | 0.464 | 0.057 | 2.75926095441877E-94 | UP-regulation |
| CPA3 | 1.38541463915355 | 0.414 | 0.019 | 1.37772456016693E-180 | UP-regulation |
| GIHCG | 1.35353954055629 | 0.751 | 0.213 | 1.84093223059228E-65 | UP-regulation |
| RNF130 | 1.32732023868137 | 0.884 | 0.484 | 6.49023086202522E-42 | UP-regulation |
| KIT | 1.32628067047236 | 0.547 | 0.129 | 5.04994659129556E-54 | UP-regulation |
| STXBP5 | 1.23044670514348 | 0.669 | 0.232 | 3.73042236550305E-39 | UP-regulation |
| NPM1 | 1.19150670128403 | 0.983 | 0.681 | 1.59171800330064E-49 | UP-regulation |
| HMGA1 | 1.16002395507048 | 0.702 | 0.243 | 1.374863016872E-41 | UP-regulation |
| HMGB1 | 1.15409046392705 | 0.972 | 0.683 | 3.45991782113732E-35 | UP-regulation |
| MOB1B | 1.15394853085003 | 0.564 | 0.143 | 7.36969785907143E-48 | UP-regulation |
| PHTF1 | 1.13898638713072 | 0.519 | 0.198 | 2.15911088747121E-22 | UP-regulation |
| HDC | 1.09746642752329 | 0.171 | 0.008 | 3.01559258570251E-68 | UP-regulation |
| ZNF385D | 1.09428088151621 | 0.227 | 0.022 | 7.32253915351952E-52 | UP-regulation |
| KIAA1211 | 1.08297375902024 | 0.564 | 0.149 | 1.89136726569207E-44 | UP-regulation |
| ABCC4 | 1.07652382979971 | 0.425 | 0.07 | 4.43704345576418E-61 | UP-regulation |
| HSPD1 | 1.06812099801476 | 0.84 | 0.424 | 6.77878709687978E-34 | UP-regulation |
| SERPINB1 | 1.06012458739294 | 0.812 | 0.551 | 2.846192616312E-20 | UP-regulation |
| STMN1 | 1.04696538561593 | 0.79 | 0.367 | 1.50925150198511E-30 | UP-regulation |
| MYC | 1.0250997816692 | 0.32 | 0.056 | 3.71843517291075E-42 | UP-regulation |
| PKIG | 1.01726083125552 | 0.425 | 0.087 | 3.84746847546591E-45 | UP-regulation |
| MYB | 1.0123052135044 | 0.552 | 0.175 | 1.58756453178827E-33 | UP-regulation |
| C1QBP | 1.00412288064264 | 0.624 | 0.269 | 2.43855208583973E-24 | UP-regulation |
| HSP90AB1 | 0.990888704085671 | 0.983 | 0.74 | 2.46943457295515E-38 | UP-regulation |
| RAN | 0.983406257523069 | 0.729 | 0.384 | 2.27947718206281E-21 | UP-regulation |
| NAA38 | 0.983021779161375 | 0.63 | 0.219 | 3.70217049684359E-34 | UP-regulation |
| HNRNPA1 | 0.982811442533765 | 0.95 | 0.678 | 2.7919692091603E-32 | UP-regulation |
| MIF | 0.980664133263432 | 0.917 | 0.594 | 1.29006141387297E-27 | UP-regulation |
| ST8SIA6 | 0.96546517754636 | 0.619 | 0.246 | 2.81084132969008E-23 | UP-regulation |
| NME1 | 0.959847876121477 | 0.508 | 0.185 | 5.7399591549387E-24 | UP-regulation |
| KLF1 | 0.95124608167551 | 0.276 | 0.015 | 2.16413745995764E-105 | UP-regulation |
| CTNNBL1 | 0.941483126629821 | 0.591 | 0.191 | 3.99957350902387E-33 | UP-regulation |
| LMO4 | 0.93826988078411 | 0.265 | 0.119 | 0.000133919456647038 | UP-regulation |
| AC069410.1 | 0.938063231176113 | 0.177 | 0.002 | 1.25374855582363E-138 | UP-regulation |
| FABP5 | 0.931685928753689 | 0.541 | 0.199 | 3.82044205412045E-25 | UP-regulation |
| RYR3 | 0.919884965537008 | 0.287 | 0.024 | 1.53488954188755E-78 | UP-regulation |
| AC002454.1 | 0.911744649145988 | 0.348 | 0.078 | 9.45175511282194E-32 | UP-regulation |
| CDK6 | 0.908725659439046 | 0.812 | 0.387 | 2.71307873977353E-26 | UP-regulation |
| PRSS57 | 0.901740113990052 | 0.724 | 0.297 | 3.88317171471587E-28 | UP-regulation |
| LDHB | 0.897742368881584 | 0.884 | 0.49 | 9.52672346894831E-28 | UP-regulation |
| NCL | 0.892901910888964 | 0.939 | 0.683 | 5.58040614915592E-24 | UP-regulation |
| EMID1 | 0.889927699174165 | 0.459 | 0.128 | 5.14154725706635E-32 | UP-regulation |
| KCNQ5 | 0.889915820414082 | 0.586 | 0.235 | 3.54972858484152E-21 | UP-regulation |
| SNHG29 | 0.872653739938749 | 0.989 | 0.724 | 1.54366138521806E-30 | UP-regulation |
| PTMA | 0.871626686484469 | 1 | 0.918 | 6.70580767001402E-42 | UP-regulation |
| SLC39A8 | 0.865376370399293 | 0.558 | 0.213 | 2.16154514098747E-22 | UP-regulation |
| S100A9 | -4.95330211213305 | 0.144 | 0.441 | 3.90586130890523E-14 | DOWN-regulation |
| S100A8 | -3.97151449934021 | 0.061 | 0.369 | 7.99911072538998E-14 | DOWN-regulation |
| VCAN | -3.08622867575583 | 0 | 0.305 | 1.06897944255905E-13 | DOWN-regulation |
| NAMPT | -2.93327657331024 | 0.271 | 0.503 | 1.27944164624506E-11 | DOWN-regulation |
| LYZ | -2.91256892816152 | 0.077 | 0.36 | 7.36654797359096E-12 | DOWN-regulation |
| TYROBP | -2.73415876276886 | 0.066 | 0.377 | 2.82111429179327E-14 | DOWN-regulation |
| CXCL8 | -2.66648338714093 | 0.055 | 0.313 | 1.1292339046651E-09 | DOWN-regulation |
| SAT1 | -2.63002604969659 | 0.26 | 0.503 | 8.00046782108897E-12 | DOWN-regulation |
| CTSS | -2.58844874589164 | 0.215 | 0.478 | 1.05532391596451E-12 | DOWN-regulation |
| G0S2 | -2.40537168882119 | 0.022 | 0.271 | 2.64439984605177E-09 | DOWN-regulation |
| FCN1 | -2.40440296900192 | 0.028 | 0.313 | 4.24779756860664E-12 | DOWN-regulation |
| ARHGAP26 | -2.35295037015611 | 0.21 | 0.499 | 2.97083815465725E-15 | DOWN-regulation |
| PLAUR | -2.28245592345698 | 0.061 | 0.328 | 1.95462765222876E-10 | DOWN-regulation |
| SLC8A1 | -2.1243410748804 | 0.011 | 0.3 | 2.86233526550005E-12 | DOWN-regulation |
| S100A12 | -2.07801806738585 | 0.028 | 0.229 | 1.83568380904705E-06 | DOWN-regulation |
| MNDA | -2.06818476704879 | 0.022 | 0.287 | 1.30283985014921E-10 | DOWN-regulation |
| S100A11 | -2.0664966076687 | 0.077 | 0.393 | 1.91200892954204E-13 | DOWN-regulation |
| FGD4 | -2.05883416259028 | 0.055 | 0.325 | 1.12564267004628E-10 | DOWN-regulation |
| DPYD | -2.04231846302147 | 0.337 | 0.674 | 1.22147149939086E-26 | DOWN-regulation |
| CRIP1 | -1.98254109921568 | 0.088 | 0.352 | 4.57853289096955E-10 | DOWN-regulation |
| LGALS2 | -1.94091874285528 | 0.017 | 0.239 | 6.32689220380489E-08 | DOWN-regulation |
| CST3 | -1.94078228981889 | 0.326 | 0.419 | 0.0435956570785016 | DOWN-regulation |
| AL589693.1 | -1.9389801479506 | 0.105 | 0.322 | 1.27660976020224E-06 | DOWN-regulation |
| IFI30 | -1.93673549086934 | 0.022 | 0.268 | 1.89829968860129E-09 | DOWN-regulation |
| FOS | -1.92106959297605 | 0.414 | 0.724 | 1.30038695574201E-24 | DOWN-regulation |
| NFKBIA | -1.88874660203896 | 0.249 | 0.551 | 8.59650245214687E-16 | DOWN-regulation |
| RBM47 | -1.82204351303325 | 0.011 | 0.268 | 5.27539581700564E-10 | DOWN-regulation |
| RAB31 | -1.82086819580342 | 0.006 | 0.26 | 3.34714437545396E-10 | DOWN-regulation |
| CD74 | -1.77817742986524 | 0.431 | 0.694 | 2.53637109172313E-22 | DOWN-regulation |
| SAMSN1 | -1.75034174179329 | 0.243 | 0.424 | 0.0000133337935340592 | DOWN-regulation |
| ACSL1 | -1.75000505122553 | 0.072 | 0.334 | 1.12394276801065E-09 | DOWN-regulation |
| NEAT1 | -1.74954722642443 | 0.564 | 0.654 | 5.03094613476796E-08 | DOWN-regulation |
| FCER1G | -1.7035849247923 | 0.099 | 0.317 | 2.48304197255881E-07 | DOWN-regulation |
| CD52 | -1.69028432120694 | 0.221 | 0.598 | 1.88501102297217E-22 | DOWN-regulation |
| LTB | -1.68453680980053 | 0.044 | 0.26 | 0.000001224407973348 | DOWN-regulation |
| AOAH | -1.67667595652479 | 0.099 | 0.338 | 1.12288770848531E-08 | DOWN-regulation |
| THBS1 | -1.64950676304975 | 0.039 | 0.203 | 0.00044864554331956 | DOWN-regulation |
| ATP2B1 | -1.64203350168312 | 0.354 | 0.522 | 1.31616760943231E-06 | DOWN-regulation |
| HLA-B | -1.63522533380503 | 0.486 | 0.752 | 3.54525033331508E-24 | DOWN-regulation |
| SLC11A1 | -1.63244653201438 | 0.022 | 0.24 | 1.86069188563396E-07 | DOWN-regulation |
| TMSB4X | -1.61114751648277 | 0.928 | 0.935 | 3.16919145618004E-25 | DOWN-regulation |
| CD83 | -1.60876874041861 | 0.088 | 0.306 | 5.03139159810421E-07 | DOWN-regulation |
| RNF149 | -1.58870294524165 | 0.166 | 0.4 | 1.02452384158323E-08 | DOWN-regulation |
| S100A10 | -1.58519026019281 | 0.182 | 0.384 | 3.36330983016463E-06 | DOWN-regulation |
| CD44 | -1.58172518098202 | 0.331 | 0.567 | 1.10446100807774E-12 | DOWN-regulation |
| PTPRE | -1.58002496702083 | 0.149 | 0.407 | 7.16925149319945E-10 | DOWN-regulation |
| SIPA1L1 | -1.57833357198833 | 0.271 | 0.46 | 6.88972267429874E-07 | DOWN-regulation |
| CSF3R | -1.56217341822945 | 0.072 | 0.339 | 6.4749566691184E-10 | DOWN-regulation |
| KLF6 | -1.55631827155367 | 0.155 | 0.456 | 4.28760739038244E-13 | DOWN-regulation |
| FYN | -1.55352140714403 | 0.171 | 0.456 | 9.28582852209345E-12 | DOWN-regulation |

**Table S7**. Top 50 up and down-regulated genes in cluster 10 in CD34+Lin-CD45+ cells.

| **gene** | **avg_log2FC** | **pct.1** | **pct.2** | **p_val_adj** | **expression** |
| --- | --- | --- | --- | --- | --- |
| GNLY | 5.86485893847815 | 0.759 | 0.012 | 0 | UP-regulation |
| NKG7 | 4.82658264766682 | 0.892 | 0.047 | 0 | UP-regulation |
| CD247 | 3.64493751760465 | 0.777 | 0.051 | 6.74806265202839E-278 | UP-regulation |
| CCL5 | 3.29132276661731 | 0.614 | 0.036 | 1.09816419497738E-228 | UP-regulation |
| SPON2 | 3.10944311260394 | 0.518 | 0.029 | 9.31474881499246E-197 | UP-regulation |
| KLRB1 | 2.98243512305999 | 0.59 | 0.008 | 0 | UP-regulation |
| GZMA | 2.93407443101017 | 0.554 | 0.015 | 0 | UP-regulation |
| CST7 | 2.80325228747751 | 0.584 | 0.009 | 0 | UP-regulation |
| CTSW | 2.75348020999084 | 0.608 | 0.067 | 1.10159865590077E-146 | UP-regulation |
| LINGO2 | 2.74505818860775 | 0.428 | 0.006 | 0 | UP-regulation |
| GZMB | 2.74008844303188 | 0.434 | 0.006 | 0 | UP-regulation |
| CEMIP2 | 2.72989820725027 | 0.723 | 0.163 | 2.23218870185687E-93 | UP-regulation |
| ARL4C | 2.7211202272925 | 0.741 | 0.154 | 3.97419250647588E-103 | UP-regulation |
| CLIC3 | 2.66061803592379 | 0.494 | 0.005 | 0 | UP-regulation |
| KLRD1 | 2.65085573403231 | 0.536 | 0.002 | 0 | UP-regulation |
| GZMM | 2.61567670247918 | 0.566 | 0.021 | 7.56903654109001E-275 | UP-regulation |
| PRF1 | 2.57773900714655 | 0.434 | 0 | 0 | UP-regulation |
| CCL4 | 2.54272286348753 | 0.325 | 0.008 | 2.4204057653293E-188 | UP-regulation |
| FGFBP2 | 2.25759040156662 | 0.313 | 0.003 | 4.40432879660831E-239 | UP-regulation |
| SAMD3 | 2.24809680568378 | 0.476 | 0.049 | 3.27221013800757E-112 | UP-regulation |
| GZMH | 2.24617474987619 | 0.319 | 0 | 0 | UP-regulation |
| RUNX3 | 2.19530530505963 | 0.554 | 0.132 | 2.96729612955215E-60 | UP-regulation |
| TGFBR3 | 2.16811433514259 | 0.319 | 0.008 | 1.10102006574282E-185 | UP-regulation |
| NCALD | 2.15578926204592 | 0.5 | 0.075 | 6.53453139502572E-84 | UP-regulation |
| CD7 | 2.15232849389253 | 0.44 | 0.05 | 1.20387029430078E-93 | UP-regulation |
| CARD11 | 2.13636826071519 | 0.47 | 0.099 | 9.01393910685754E-54 | UP-regulation |
| DUSP2 | 2.12847997122929 | 0.53 | 0.145 | 4.74982968378503E-47 | UP-regulation |
| XCL2 | 2.1235555510383 | 0.307 | 0 | 0 | UP-regulation |
| TRBC1 | 2.121905096378 | 0.452 | 0.037 | 7.01667696797678E-124 | UP-regulation |
| STAT4 | 2.11470805735812 | 0.44 | 0.038 | 3.54855977852777E-118 | UP-regulation |
| TGFB1 | 2.08891352799219 | 0.542 | 0.195 | 5.90559106254991E-36 | UP-regulation |
| CALM1 | 2.08112799583439 | 0.873 | 0.527 | 1.22705245189516E-56 | UP-regulation |
| PYHIN1 | 2.06040369823328 | 0.386 | 0.013 | 5.51616434773666E-192 | UP-regulation |
| SKAP1 | 2.00304014319821 | 0.693 | 0.237 | 1.05419144520433E-53 | UP-regulation |
| CD96 | 1.99036092702884 | 0.416 | 0.052 | 7.35825085941466E-77 | UP-regulation |
| SYNE2 | 1.97819272175066 | 0.355 | 0.035 | 3.94347945217684E-82 | UP-regulation |
| SYNE1 | 1.96232283557385 | 0.506 | 0.125 | 1.69790574875831E-49 | UP-regulation |
| IL32 | 1.93591542083199 | 0.331 | 0.051 | 7.50058264523648E-48 | UP-regulation |
| SLC38A1 | 1.92154796968341 | 0.608 | 0.205 | 7.02360385831292E-45 | UP-regulation |
| KLRF1 | 1.9088644479739 | 0.295 | 0.001 | 9.81984574096115E-270 | UP-regulation |
| MYBL1 | 1.88038771949885 | 0.319 | 0.004 | 9.61802418732334E-234 | UP-regulation |
| JAK1 | 1.87798891448402 | 0.843 | 0.519 | 7.44498709838026E-50 | UP-regulation |
| RASA3 | 1.87255470088286 | 0.524 | 0.177 | 9.20659804130984E-35 | UP-regulation |
| DDIT4 | 1.87106815275614 | 0.59 | 0.249 | 2.82184400678265E-30 | UP-regulation |
| ABHD17A | 1.85659136724179 | 0.452 | 0.144 | 4.0587110082643E-31 | UP-regulation |
| GNG2 | 1.8463454599233 | 0.596 | 0.176 | 4.96256405724021E-46 | UP-regulation |
| TXNIP | 1.84349027596552 | 0.711 | 0.312 | 5.48340876823227E-40 | UP-regulation |
| HCST | 1.84261770224465 | 0.627 | 0.267 | 3.77408083031413E-34 | UP-regulation |
| MATK | 1.83956220769883 | 0.386 | 0.107 | 1.13775405585295E-30 | UP-regulation |
| NCR3 | 1.80803008205026 | 0.271 | 0.008 | 2.36033924493051E-143 | UP-regulation |
| S100A9 | -3.48050842023271 | 0.096 | 0.442 | 1.94659082169501E-14 | DOWN-regulation |
| S100A8 | -2.96143988585569 | 0.072 | 0.367 | 8.85413468515483E-11 | DOWN-regulation |
| LYZ | -2.84723241622488 | 0.036 | 0.36 | 5.0634225430632E-13 | DOWN-regulation |
| NKAIN2 | -2.72265926974016 | 0.036 | 0.437 | 3.88532362749807E-19 | DOWN-regulation |
| HLA-DRA | -2.64145318611113 | 0.06 | 0.682 | 7.37096585058106E-41 | DOWN-regulation |
| NAMPT | -2.59661142798004 | 0.151 | 0.506 | 5.4546247287444E-15 | DOWN-regulation |
| CST3 | -2.52864703251418 | 0.066 | 0.428 | 2.09679756828717E-15 | DOWN-regulation |
| AIF1 | -2.51968932241672 | 0.072 | 0.707 | 4.82962464386901E-43 | DOWN-regulation |
| VCAN | -2.40724086246153 | 0.042 | 0.302 | 1.29472857756158E-08 | DOWN-regulation |
| G0S2 | -2.3713168234298 | 0.03 | 0.27 | 1.0921973616683E-07 | DOWN-regulation |
| FTH1 | -2.23332262328065 | 0.759 | 0.972 | 8.58876894742379E-43 | DOWN-regulation |
| FCN1 | -2.21403703320519 | 0.03 | 0.312 | 2.68653001993952E-10 | DOWN-regulation |
| AL589693.1 | -2.17938110714448 | 0.03 | 0.324 | 1.35079463761065E-10 | DOWN-regulation |
| GAB2 | -2.0976385241667 | 0.066 | 0.561 | 3.27291801853753E-26 | DOWN-regulation |
| SAT1 | -2.07441991387566 | 0.223 | 0.504 | 6.34231435358899E-09 | DOWN-regulation |
| CXCL8 | -2.05693970915922 | 0.054 | 0.313 | 5.11076424745844E-08 | DOWN-regulation |
| HLA-DRB1 | -2.04452147462601 | 0.078 | 0.572 | 3.85519838024706E-26 | DOWN-regulation |
| PLAUR | -1.98426869118881 | 0.042 | 0.328 | 7.22794300327322E-10 | DOWN-regulation |
| MNDA | -1.97985358854261 | 0.024 | 0.287 | 3.58555995068199E-09 | DOWN-regulation |
| LRMDA | -1.95277606393556 | 0.066 | 0.547 | 7.57454318528632E-25 | DOWN-regulation |
| FGD4 | -1.94002453778856 | 0.048 | 0.325 | 1.21465003219825E-09 | DOWN-regulation |
| SOD2 | -1.93269176832914 | 0.187 | 0.659 | 7.47067584475174E-24 | DOWN-regulation |
| CTSS | -1.88607055632203 | 0.187 | 0.478 | 6.22016757639583E-10 | DOWN-regulation |
| IL1B | -1.84749448005381 | 0.018 | 0.26 | 6.35664734562383E-08 | DOWN-regulation |
| MCTP1 | -1.84089084600955 | 0.03 | 0.443 | 9.07173774813391E-20 | DOWN-regulation |
| RBM47 | -1.83814968559252 | 0.012 | 0.267 | 7.00861977134603E-09 | DOWN-regulation |
| VIM | -1.81100525014941 | 0.319 | 0.83 | 8.16052079081118E-36 | DOWN-regulation |
| CD74 | -1.80595193566138 | 0.217 | 0.701 | 2.43061713122724E-26 | DOWN-regulation |
| SAMSN1 | -1.80517647157999 | 0.09 | 0.429 | 1.56592040608844E-12 | DOWN-regulation |
| IFI30 | -1.77527762040273 | 0.03 | 0.267 | 1.71102527660593E-07 | DOWN-regulation |
| CASC15 | -1.77179899982735 | 0.048 | 0.386 | 2.24480345087274E-13 | DOWN-regulation |
| SLC8A1 | -1.76868768118446 | 0.036 | 0.298 | 1.29836909118713E-08 | DOWN-regulation |
| S100A6 | -1.76252441494448 | 0.217 | 0.494 | 1.02267445438503E-08 | DOWN-regulation |
| PLXDC2 | -1.75443333229131 | 0.078 | 0.427 | 9.85477101247075E-14 | DOWN-regulation |
| SNHG29 | -1.73646729322944 | 0.38 | 0.747 | 6.52669101263323E-24 | DOWN-regulation |
| LST1 | -1.71488736850998 | 0.054 | 0.414 | 1.03691802217883E-14 | DOWN-regulation |
| LGALS2 | -1.71397184504596 | 0.024 | 0.238 | 4.12516378284455E-06 | DOWN-regulation |
| ETV6 | -1.71225959250238 | 0.175 | 0.65 | 2.43172564288245E-24 | DOWN-regulation |
| BASP1 | -1.70926947649064 | 0.024 | 0.341 | 4.05181030135402E-12 | DOWN-regulation |
| INPP4B | -1.70122799930316 | 0.054 | 0.395 | 4.14533779746448E-13 | DOWN-regulation |
| MSI2 | -1.69409019218083 | 0.157 | 0.508 | 2.65452693150976E-14 | DOWN-regulation |
| TKT | -1.68957163490308 | 0.175 | 0.694 | 9.28810686915338E-28 | DOWN-regulation |
| GP1BB | -1.68014603925941 | 0.018 | 0.236 | 2.04645484015081E-06 | DOWN-regulation |
| THBS1 | -1.66898903073382 | 0.018 | 0.203 | 0.0000812589907225679 | DOWN-regulation |
| S100A12 | -1.65874992438414 | 0.036 | 0.228 | 0.000130515404475664 | DOWN-regulation |
| FTL | -1.64059737567855 | 0.831 | 0.966 | 2.50776363192715E-21 | DOWN-regulation |
| ACSL1 | -1.63541922700652 | 0.054 | 0.334 | 3.53181629296282E-09 | DOWN-regulation |
| RNF130 | -1.634973448959 | 0.09 | 0.514 | 9.35625667370336E-19 | DOWN-regulation |
| CALN1 | -1.6296485457969 | 0.024 | 0.311 | 1.1842559273175E-10 | DOWN-regulation |
| NRIP1 | -1.62243693383277 | 0.199 | 0.55 | 2.86529033493709E-14 | DOWN-regulation |

**Table S8.** Top 50 up and down-regulated genes in cluster 11 in CD34+Lin-CD45+ cells.

| **gene** | **avg_log2FC** | **pct.1** | **pct.2** | **p_val_adj** | **expression** |
| --- | --- | --- | --- | --- | --- |
| HBG2 | 9.42536253718355 | 0.99 | 0.162 | 4.641411706792E-141 | UP-regulation |
| HBB | 9.28944547207259 | 1 | 0.108 | 8.56760392657042E-194 | UP-regulation |
| HBA2 | 8.6807886797623 | 1 | 0.132 | 4.08254632519722E-167 | UP-regulation |
| HBG1 | 8.63537520671232 | 1 | 0.072 | 1.687685488016E-257 | UP-regulation |
| HBA1 | 8.47338463893494 | 1 | 0.079 | 2.03185232483019E-243 | UP-regulation |
| HBM | 6.23327765380441 | 0.961 | 0.01 | 0 | UP-regulation |
| AHSP | 5.38702120314803 | 0.99 | 0.005 | 0 | UP-regulation |
| ALAS2 | 4.56086247546968 | 0.961 | 0.005 | 0 | UP-regulation |
| HBD | 4.24738198188092 | 0.282 | 0.015 | 6.37840298023185E-77 | UP-regulation |
| BLVRB | 3.93178686767231 | 1 | 0.203 | 2.20006126766403E-119 | UP-regulation |
| PRDX2 | 3.57131134546504 | 0.971 | 0.22 | 1.65783137452822E-95 | UP-regulation |
| SLC4A1 | 3.23536709613407 | 0.786 | 0.002 | 0 | UP-regulation |
| BPGM | 3.17548825261078 | 0.864 | 0.034 | 0 | UP-regulation |
| SELENBP1 | 3.14764339669262 | 0.893 | 0.001 | 0 | UP-regulation |
| SLC25A37 | 3.07819272635316 | 0.971 | 0.177 | 8.18313533151761E-115 | UP-regulation |
| SLC25A39 | 2.96288723699417 | 0.913 | 0.071 | 7.6107400420477E-211 | UP-regulation |
| SNCA | 2.95620246726207 | 0.942 | 0.154 | 2.97208901125381E-122 | UP-regulation |
| UBB | 2.88252095759718 | 0.981 | 0.664 | 3.92628444574769E-51 | UP-regulation |
| HEMGN | 2.84804483405635 | 0.777 | 0.094 | 7.74747809541869E-116 | UP-regulation |
| CA1 | 2.75049972019606 | 0.291 | 0.006 | 1.12931645859775E-148 | UP-regulation |
| GYPB | 2.68412997231554 | 0.728 | 0.001 | 0 | UP-regulation |
| IFIT1B | 2.63891721403958 | 0.427 | 0.001 | 0 | UP-regulation |
| FAM210B | 2.51244073320605 | 0.806 | 0.029 | 0 | UP-regulation |
| EPB42 | 2.48659410665044 | 0.738 | 0.001 | 0 | UP-regulation |
| MYL4 | 2.4580329419458 | 0.699 | 0.003 | 0 | UP-regulation |
| BSG | 2.38989905034284 | 0.864 | 0.19 | 5.86964036281771E-80 | UP-regulation |
| HMBS | 2.37382233805829 | 0.505 | 0.035 | 6.19701438177921E-113 | UP-regulation |
| GLRX5 | 2.35576151327445 | 0.883 | 0.139 | 3.83719678423224E-110 | UP-regulation |
| HBZ | 2.11789575482336 | 0.175 | 0.002 | 7.58470042473892E-120 | UP-regulation |
| GYPA | 2.09767551631588 | 0.417 | 0 | 0 | UP-regulation |
| HAGH | 2.034827700596 | 0.718 | 0.08 | 1.58868416081201E-108 | UP-regulation |
| RBM38 | 1.99051436375151 | 0.66 | 0.083 | 1.1118784639361E-87 | UP-regulation |
| TRIM58 | 1.91926664411504 | 0.592 | 0.038 | 1.67087504619175E-142 | UP-regulation |
| NCOA4 | 1.90454211635418 | 0.893 | 0.245 | 2.37605013872873E-60 | UP-regulation |
| FECH | 1.87312525502384 | 0.573 | 0.02 | 2.0158802562688E-220 | UP-regulation |
| GABARAPL2 | 1.86576506750307 | 0.845 | 0.264 | 2.00826311792225E-51 | UP-regulation |
| BNIP3L | 1.80754042107486 | 0.903 | 0.317 | 2.99689305406038E-48 | UP-regulation |
| TENT5C | 1.77472149130471 | 0.592 | 0.032 | 1.10263741611675E-163 | UP-regulation |
| GUK1 | 1.75440831815495 | 0.942 | 0.345 | 4.82588600711604E-47 | UP-regulation |
| PITHD1 | 1.75051941348517 | 0.553 | 0.114 | 9.55697706194203E-41 | UP-regulation |
| GYPC | 1.73507254941642 | 0.922 | 0.434 | 2.5958204910264E-33 | UP-regulation |
| HBQ1 | 1.73064822467137 | 0.495 | 0.003 | 0 | UP-regulation |
| GSPT1 | 1.6871708875811 | 0.748 | 0.249 | 6.79914793050959E-35 | UP-regulation |
| GMPR | 1.68121708385214 | 0.583 | 0.039 | 1.02852985504086E-133 | UP-regulation |
| SMOX | 1.64303529933991 | 0.447 | 0.021 | 3.93028179369106E-132 | UP-regulation |
| DCAF12 | 1.64290807163132 | 0.631 | 0.036 | 7.20789839128404E-168 | UP-regulation |
| MPP1 | 1.63848320480826 | 0.777 | 0.156 | 1.83409084513501E-63 | UP-regulation |
| FKBP8 | 1.631414367883 | 0.883 | 0.269 | 1.17388041347792E-48 | UP-regulation |
| MKRN1 | 1.54355206220436 | 0.709 | 0.157 | 2.57768601771184E-49 | UP-regulation |
| KRT1 | 1.53319834983147 | 0.369 | 0.005 | 6.32307288022135E-230 | UP-regulation |
| S100A9 | -4.72231231582457 | 0.282 | 0.433 | 0.0218661473493888 | DOWN-regulation |
| TMSB4X | -4.28677315839958 | 0.223 | 0.951 | 3.02798329204883E-52 | DOWN-regulation |
| S100A8 | -3.89255245008114 | 0.107 | 0.363 | 0.0000936475325986372 | DOWN-regulation |
| EEF1A1 | -3.67265748632574 | 0.466 | 0.962 | 8.70919093463412E-52 | DOWN-regulation |
| TMSB10 | -3.54485192477011 | 0.184 | 0.887 | 1.49225955435321E-44 | DOWN-regulation |
| RPL34 | -3.52819661396608 | 0.32 | 0.942 | 9.47902913472669E-51 | DOWN-regulation |
| NAMPT | -3.52148387560961 | 0.087 | 0.503 | 1.97790524163786E-12 | DOWN-regulation |
| RPL10 | -3.51685689621067 | 0.709 | 0.974 | 1.35705829853217E-52 | DOWN-regulation |
| RPS4X | -3.51677552344565 | 0.563 | 0.951 | 1.26678504157083E-47 | DOWN-regulation |
| VIM | -3.49164459777805 | 0.107 | 0.828 | 3.11491663964841E-39 | DOWN-regulation |
| B2M | -3.47087215542155 | 0.32 | 0.969 | 1.0183391941186E-52 | DOWN-regulation |
| RPS8 | -3.41757992720141 | 0.369 | 0.952 | 9.86553935157254E-50 | DOWN-regulation |
| RPL39 | -3.37939756825259 | 0.388 | 0.946 | 1.32196794451712E-50 | DOWN-regulation |
| RPS23 | -3.36034025299962 | 0.437 | 0.932 | 3.05658565154291E-46 | DOWN-regulation |
| RPS14 | -3.34784122852698 | 0.534 | 0.961 | 1.39372804651418E-51 | DOWN-regulation |
| RPL26 | -3.34223446183202 | 0.311 | 0.948 | 3.08655215401826E-49 | DOWN-regulation |
| RPS18 | -3.31234872834492 | 0.544 | 0.94 | 1.21160191866917E-45 | DOWN-regulation |
| RPS9 | -3.28781391703105 | 0.447 | 0.958 | 1.47099645030158E-52 | DOWN-regulation |
| RPL19 | -3.2545566264072 | 0.427 | 0.948 | 3.37911646120885E-49 | DOWN-regulation |
| RPL13 | -3.25288845384647 | 0.66 | 0.962 | 3.52444992512208E-49 | DOWN-regulation |
| RPS3A | -3.25259589122576 | 0.553 | 0.953 | 3.16348566840526E-48 | DOWN-regulation |
| RPS27 | -3.2433650457911 | 0.398 | 0.946 | 1.19588464933462E-50 | DOWN-regulation |
| RPL9 | -3.23678088323354 | 0.311 | 0.924 | 2.29142076160719E-47 | DOWN-regulation |
| RPL3 | -3.23520981923524 | 0.388 | 0.915 | 1.93302910495265E-42 | DOWN-regulation |
| RPL7A | -3.22174194522587 | 0.379 | 0.932 | 8.70698697165989E-46 | DOWN-regulation |
| AREG | -3.22120804126144 | 0.039 | 0.566 | 6.95812514579407E-19 | DOWN-regulation |
| RACK1 | -3.21128596148507 | 0.369 | 0.914 | 4.60605105947849E-44 | DOWN-regulation |
| RPL29 | -3.20579814276033 | 0.485 | 0.945 | 1.46953980396841E-47 | DOWN-regulation |
| S100A4 | -3.17054724841887 | 0.155 | 0.7 | 2.16961795756132E-23 | DOWN-regulation |
| RPL5 | -3.16242408415077 | 0.34 | 0.912 | 1.72643051621277E-43 | DOWN-regulation |
| RPL12 | -3.16216316556722 | 0.427 | 0.934 | 9.65424554013253E-46 | DOWN-regulation |
| SRGN | -3.1610052076907 | 0.049 | 0.642 | 2.26041392047453E-23 | DOWN-regulation |
| RPS15A | -3.15119819592915 | 0.447 | 0.946 | 2.74856304165165E-48 | DOWN-regulation |
| RPL18 | -3.12522694575889 | 0.33 | 0.947 | 1.44006070135706E-47 | DOWN-regulation |
| FOS | -3.10898760258184 | 0.117 | 0.726 | 1.44293741719472E-27 | DOWN-regulation |
| RPL11 | -3.09772750425935 | 0.573 | 0.96 | 9.49706736169549E-49 | DOWN-regulation |
| RPL32 | -3.09721549224314 | 0.485 | 0.955 | 2.81795575396758E-47 | DOWN-regulation |
| DPYD | -3.09685632427711 | 0.039 | 0.675 | 4.4936912731697E-27 | DOWN-regulation |
| MT-CO2 | -3.09232596390943 | 0.641 | 0.977 | 1.94604283167373E-53 | DOWN-regulation |
| RPL10A | -3.07096326085166 | 0.252 | 0.862 | 7.83578779353645E-38 | DOWN-regulation |
| ACTG1 | -3.07045519895008 | 0.107 | 0.839 | 5.50201364463783E-39 | DOWN-regulation |
| LYZ | -3.06751864829643 | 0.058 | 0.355 | 0.0000033425549428469 | DOWN-regulation |
| MALAT1 | -3.06032916119289 | 0.602 | 0.995 | 1.53252318518752E-50 | DOWN-regulation |
| RPS27A | -3.052958447063 | 0.398 | 0.944 | 2.08618663007318E-47 | DOWN-regulation |
| CXCL8 | -3.02587150625753 | 0.019 | 0.31 | 6.29878996632578E-06 | DOWN-regulation |
| RPL28 | -3.01589520703972 | 0.563 | 0.963 | 1.76414499662228E-50 | DOWN-regulation |
| RPS24 | -3.01196480830899 | 0.553 | 0.961 | 3.67334310343977E-49 | DOWN-regulation |
| HLA-DRA | -3.01028801393958 | 0.049 | 0.674 | 2.95797155402319E-26 | DOWN-regulation |
| RPL15 | -3.00085747811393 | 0.369 | 0.934 | 1.70035021209829E-45 | DOWN-regulation |
| TYROBP | -2.99860016550578 | 0.01 | 0.373 | 3.3475176307145E-09 | DOWN-regulation |

**Table S9.** Top 50 up and down-regulated genes in cluster 13 in CD34+Lin-CD45+ cells.

| **gene** | **avg_log2FC** | **pct.1** | **pct.2** | **p_val_adj** | **expression** |
| --- | --- | --- | --- | --- | --- |
| IGKC | 5.31863834444848 | 0.635 | 0.007 | 0 | UP-regulation |
| IGLC2 | 3.86789579530431 | 0.435 | 0.005 | 9.42203119785562E-277 | UP-regulation |
| BANK1 | 3.85306753412745 | 0.929 | 0.044 | 7.85039317186993E-271 | UP-regulation |
| IGHM | 3.72617297929424 | 0.941 | 0.147 | 1.64608145621026E-110 | UP-regulation |
| IGLC3 | 3.36358327605295 | 0.341 | 0.004 | 6.56315069287777E-207 | UP-regulation |
| RALGPS2 | 3.34572464242695 | 0.882 | 0.121 | 1.44031113003436E-110 | UP-regulation |
| BACH2 | 3.29697537405517 | 0.976 | 0.256 | 8.00142439462668E-70 | UP-regulation |
| IGHD | 3.2615099074351 | 0.835 | 0.041 | 1.64956155672643E-232 | UP-regulation |
| MS4A1 | 3.20288222767965 | 0.812 | 0.004 | 0 | UP-regulation |
| IGLC1 | 3.10096508090918 | 0.353 | 0.002 | 4.68513835611271E-276 | UP-regulation |
| OSBPL10 | 3.07305090657402 | 0.812 | 0.033 | 4.45025968724212E-257 | UP-regulation |
| AFF3 | 3.01809404041382 | 0.941 | 0.368 | 1.59838063457066E-51 | UP-regulation |
| EBF1 | 2.9799826667819 | 0.753 | 0.022 | 2.87869279232264E-285 | UP-regulation |
| TCL1A | 2.95496751757584 | 0.718 | 0 | 0 | UP-regulation |
| PAX5 | 2.93074166503743 | 0.635 | 0.003 | 0 | UP-regulation |
| NIBAN3 | 2.90439227636029 | 0.753 | 0.019 | 0 | UP-regulation |
| CD79B | 2.88785513858014 | 0.871 | 0.063 | 1.42103548659732E-180 | UP-regulation |
| CD79A | 2.76049497151861 | 0.706 | 0.024 | 5.72513252636143E-247 | UP-regulation |
| COL19A1 | 2.50240829286189 | 0.541 | 0.001 | 0 | UP-regulation |
| PLEKHG1 | 2.47942352683047 | 0.647 | 0.033 | 4.54804734132954E-166 | UP-regulation |
| CAMK2D | 2.43424557992861 | 0.765 | 0.125 | 1.48876564304423E-71 | UP-regulation |
| GNG7 | 2.28425820461137 | 0.706 | 0.09 | 6.50276360233931E-85 | UP-regulation |
| LINC00926 | 2.2244415420066 | 0.529 | 0.008 | 1.82391985270352E-284 | UP-regulation |
| HLA-DQA1 | 2.22294088671422 | 0.694 | 0.093 | 2.2474604367575E-76 | UP-regulation |
| FCRL1 | 2.21049398147337 | 0.576 | 0 | 0 | UP-regulation |
| LTB | 2.1701764309293 | 0.941 | 0.239 | 4.66030905796673E-57 | UP-regulation |
| ARHGAP24 | 2.15695626561473 | 0.788 | 0.176 | 9.65539147160549E-48 | UP-regulation |
| HLA-DPB1 | 2.14651558013173 | 0.976 | 0.389 | 6.53623995838915E-48 | UP-regulation |
| SP140 | 2.06897407750155 | 0.553 | 0.018 | 4.22015102305155E-193 | UP-regulation |
| ANGPTL1 | 1.99426403139598 | 0.553 | 0.035 | 7.571577067024E-118 | UP-regulation |
| BLK | 1.95241590809748 | 0.459 | 0.009 | 2.29742016018509E-217 | UP-regulation |
| ZCCHC7 | 1.93401485516844 | 0.706 | 0.248 | 9.44927412550406E-27 | UP-regulation |
| PRKCE | 1.92018386760625 | 0.729 | 0.255 | 2.48815577701759E-27 | UP-regulation |
| SNX29 | 1.89420678784334 | 0.753 | 0.28 | 3.89895039257884E-28 | UP-regulation |
| CDK14 | 1.88211972877118 | 0.506 | 0.07 | 3.04010201540898E-49 | UP-regulation |
| CARD11 | 1.87326494009046 | 0.6 | 0.103 | 1.02376465428273E-46 | UP-regulation |
| MTSS1 | 1.84922317623306 | 0.682 | 0.17 | 6.54421527983729E-36 | UP-regulation |
| FCHSD2 | 1.83844186364107 | 0.859 | 0.386 | 2.96210089079734E-28 | UP-regulation |
| VPREB3 | 1.81296449119234 | 0.447 | 0.002 | 0 | UP-regulation |
| EIF2AK3 | 1.81174690022104 | 0.659 | 0.142 | 7.59445969896498E-40 | UP-regulation |
| STRBP | 1.78455910442253 | 0.682 | 0.228 | 2.3709012516423E-26 | UP-regulation |
| GNB5 | 1.76512809798923 | 0.518 | 0.077 | 2.36814569759069E-47 | UP-regulation |
| SIPA1L3 | 1.76136260784024 | 0.706 | 0.227 | 1.77128745869924E-28 | UP-regulation |
| TPD52 | 1.71845508427542 | 0.482 | 0.046 | 1.47380636787966E-69 | UP-regulation |
| CD37 | 1.71301747526454 | 0.941 | 0.601 | 8.66484930746201E-30 | UP-regulation |
| PRKCB | 1.70439704091873 | 0.859 | 0.471 | 2.43379157700333E-23 | UP-regulation |
| PCDH9 | 1.69918760328879 | 0.341 | 0.021 | 6.21029094174305E-70 | UP-regulation |
| P2RX5 | 1.68862783151372 | 0.388 | 0.012 | 6.19735229431506E-141 | UP-regulation |
| WDFY4 | 1.68653998376543 | 0.506 | 0.095 | 1.53208153447912E-35 | UP-regulation |
| HERPUD1 | 1.68459661925038 | 0.624 | 0.173 | 7.07389684700485E-27 | UP-regulation |
| S100A9 | -4.22137086812577 | 0.176 | 0.434 | 0.00143414254707327 | DOWN-regulation |
| S100A8 | -3.67405697824005 | 0.094 | 0.362 | 0.00126654975690806 | DOWN-regulation |
| AREG | -3.17638092593132 | 0.047 | 0.564 | 1.00279000072635E-14 | DOWN-regulation |
| LYZ | -3.13463320379774 | 0.035 | 0.355 | 0.0000232285137175226 | DOWN-regulation |
| TYROBP | -2.80511280049672 | 0.035 | 0.372 | 4.70330857930066E-06 | DOWN-regulation |
| CST3 | -2.76687529929333 | 0.082 | 0.421 | 3.21080895465914E-06 | DOWN-regulation |
| S100A6 | -2.71274013689209 | 0.165 | 0.49 | 3.44475381791758E-06 | DOWN-regulation |
| VCAN | -2.66086011097668 | 0.024 | 0.298 | 0.00111674225046122 | DOWN-regulation |
| CXCL8 | -2.64949233688257 | 0.047 | 0.308 | 0.00412581572090801 | DOWN-regulation |
| AL589693.1 | -2.55785310548768 | 0.012 | 0.319 | 0.0000638121191474078 | DOWN-regulation |
| NKAIN2 | -2.55455254844798 | 0.071 | 0.43 | 5.63931013841235E-07 | DOWN-regulation |
| SRGN | -2.52403251291084 | 0.118 | 0.638 | 2.46031247382212E-14 | DOWN-regulation |
| S100A4 | -2.50132741856061 | 0.282 | 0.695 | 4.48701716555171E-12 | DOWN-regulation |
| AIF1 | -2.49495213886329 | 0.071 | 0.696 | 1.45554410328343E-20 | DOWN-regulation |
| PLCB1 | -2.39088442245335 | 0.059 | 0.587 | 2.274519417427E-14 | DOWN-regulation |
| FCN1 | -2.29251664497177 | 0.035 | 0.307 | 0.00111509023158676 | DOWN-regulation |
| S100A11 | -2.25906882745289 | 0.059 | 0.387 | 0.0000156759861584789 | DOWN-regulation |
| LRMDA | -2.21740927063814 | 0.047 | 0.54 | 8.01988079962851E-13 | DOWN-regulation |
| PLXDC2 | -2.16723004833189 | 0.035 | 0.422 | 8.28658799457259E-08 | DOWN-regulation |
| FTH1 | -2.14617228903093 | 0.894 | 0.966 | 1.42119456986608E-19 | DOWN-regulation |
| S100A10 | -2.10861643275922 | 0.082 | 0.382 | 0.0000998945412883371 | DOWN-regulation |
| INPP4B | -2.09343746954206 | 0.024 | 0.39 | 5.3849393050092E-07 | DOWN-regulation |
| LGALS1 | -2.09221419881938 | 0.059 | 0.323 | 0.00221157554941492 | DOWN-regulation |
| NEAT1 | -2.06543436085869 | 0.318 | 0.657 | 5.79918009291594E-08 | DOWN-regulation |
| SLC8A1 | -2.0142437271817 | 0.024 | 0.294 | 0.00153072417042613 | DOWN-regulation |
| FGD4 | -2.01064394735047 | 0.035 | 0.32 | 0.000697061148631781 | DOWN-regulation |
| FCER1G | -1.99100485643178 | 0.012 | 0.315 | 0.0000818992497723165 | DOWN-regulation |
| ARHGAP26 | -1.9867607525851 | 0.165 | 0.494 | 0.0000165420621382011 | DOWN-regulation |
| ANXA1 | -1.9668103583955 | 0.035 | 0.384 | 5.24308675459885E-06 | DOWN-regulation |
| MNDA | -1.96471514152255 | 0.035 | 0.282 | 0.00866059158210256 | DOWN-regulation |
| TSPO | -1.96118536568847 | 0.118 | 0.532 | 2.88400969982051E-09 | DOWN-regulation |
| NRIP1 | -1.93835138166123 | 0.212 | 0.544 | 5.78122733254249E-07 | DOWN-regulation |
| ACSL1 | -1.91550005008406 | 0.059 | 0.329 | 0.00191572198397445 | DOWN-regulation |
| CASC15 | -1.89394878426423 | 0.035 | 0.38 | 4.62718365965939E-06 | DOWN-regulation |
| SOD2 | -1.85193614893086 | 0.318 | 0.649 | 1.95405114351717E-07 | DOWN-regulation |
| PRKCH | -1.84011036445183 | 0.059 | 0.412 | 1.18960916877687E-06 | DOWN-regulation |
| SAMSN1 | -1.83519432335546 | 0.082 | 0.423 | 0.0000191076196722972 | DOWN-regulation |
| GAPDH | -1.81599968501713 | 0.565 | 0.843 | 6.25328427174763E-17 | DOWN-regulation |
| IL1B | -1.81551307597752 | 0.012 | 0.256 | 0.0102741925233964 | DOWN-regulation |
| GP1BB | -1.75704278575484 | 0.012 | 0.233 | 0.0419345411624425 | DOWN-regulation |
| PLAUR | -1.74205130765395 | 0.059 | 0.323 | 0.012348808591131 | DOWN-regulation |
| AOAH | -1.7346612931616 | 0.047 | 0.334 | 0.00057058057828987 | DOWN-regulation |
| LST1 | -1.72155705885562 | 0.082 | 0.407 | 0.0000294910796681233 | DOWN-regulation |
| GNAQ | -1.70565193077359 | 0.059 | 0.507 | 4.17059210339292E-10 | DOWN-regulation |
| GSTP1 | -1.69345556725982 | 0.294 | 0.7 | 3.10093065503486E-12 | DOWN-regulation |
| FNDC3B | -1.69310576462951 | 0.059 | 0.369 | 0.000191342739123127 | DOWN-regulation |
| SLCO3A1 | -1.68710867678425 | 0.035 | 0.354 | 0.0000434625710937198 | DOWN-regulation |
| SPINK2 | -1.68175555104387 | 0.024 | 0.353 | 8.85009514772752E-06 | DOWN-regulation |
| RILPL2 | -1.67636795132148 | 0.212 | 0.58 | 1.73370532467471E-08 | DOWN-regulation |
| VIM | -1.67275297080688 | 0.553 | 0.817 | 4.37103531397927E-12 | DOWN-regulation |

**Table S10**. Top 50 up and down-regulated genes in cluster 2 in CD34+Lin-CD45+ cells.

| **gene** | **avg_log2FC** | **pct.1** | **pct.2** | **p_val_adj** | **expression** |
| --- | --- | --- | --- | --- | --- |
| VCAN | 2.69733194328823 | 0.956 | 0.2 | 0 | UP-regulation |
| S100A9 | 2.49049372408887 | 1 | 0.349 | 1.60969688572428E-299 | UP-regulation |
| CXCL8 | 2.31805549960422 | 0.945 | 0.213 | 0 | UP-regulation |
| S100A8 | 2.22139488125112 | 0.997 | 0.266 | 0 | UP-regulation |
| NAMPT | 2.21125342328528 | 1 | 0.422 | 3.07167932587555E-282 | UP-regulation |
| S100A12 | 2.12452849346002 | 0.822 | 0.136 | 0 | UP-regulation |
| G0S2 | 2.1023428746723 | 0.851 | 0.178 | 7.77632232973808E-268 | UP-regulation |
| THBS1 | 2.07845076383033 | 0.705 | 0.125 | 3.77477152736814E-244 | UP-regulation |
| KYNU | 2.06632557783168 | 0.752 | 0.117 | 1.46644303886316E-293 | UP-regulation |
| PLAUR | 2.06365356488896 | 0.915 | 0.234 | 1.06133043128919E-270 | UP-regulation |
| SLC11A1 | 2.05630745443906 | 0.831 | 0.147 | 0 | UP-regulation |
| FGD4 | 2.05244239281772 | 0.911 | 0.231 | 7.32576613311528E-280 | UP-regulation |
| IL1B | 2.03631338055175 | 0.714 | 0.186 | 4.90069814883324E-186 | UP-regulation |
| CTSS | 2.0302882446927 | 0.99 | 0.394 | 2.04713331138254E-257 | UP-regulation |
| ARHGAP26 | 2.01523863505066 | 0.98 | 0.418 | 7.0103023727184E-252 | UP-regulation |
| ACSL1 | 2.01098720614882 | 0.879 | 0.245 | 2.27649772985831E-252 | UP-regulation |
| RAB31 | 1.93386694424298 | 0.819 | 0.17 | 1.39350806781925E-265 | UP-regulation |
| SLC8A1 | 1.92712920931476 | 0.844 | 0.21 | 3.8729160573433E-239 | UP-regulation |
| LYZ | 1.91305095807041 | 0.982 | 0.259 | 2.59404466988896E-274 | UP-regulation |
| MNDA | 1.90637404065495 | 0.894 | 0.19 | 1.06470993822054E-282 | UP-regulation |
| DMXL2 | 1.90321816311423 | 0.777 | 0.164 | 5.85873149755185E-250 | UP-regulation |
| SAMSN1 | 1.88057859031848 | 0.923 | 0.345 | 1.93162676092589E-227 | UP-regulation |
| RBM47 | 1.87280533776247 | 0.812 | 0.179 | 1.90440117190923E-250 | UP-regulation |
| ATP13A3 | 1.86513123699769 | 0.836 | 0.217 | 1.05916587215894E-231 | UP-regulation |
| ATP2B1 | 1.86205493283068 | 0.931 | 0.456 | 1.41711280459854E-192 | UP-regulation |
| AQP9 | 1.85646176335091 | 0.693 | 0.092 | 1.69665570321355E-293 | UP-regulation |
| ATP2B1-AS1 | 1.83473735000466 | 0.749 | 0.173 | 4.12498681485636E-224 | UP-regulation |
| ANXA1 | 1.81700825165509 | 0.881 | 0.307 | 1.07202076353628E-195 | UP-regulation |
| SIPA1L1 | 1.81234634036757 | 0.933 | 0.385 | 2.04307005533987E-211 | UP-regulation |
| PLXDC2 | 1.79947752938834 | 0.918 | 0.344 | 4.34878181964584E-210 | UP-regulation |
| S100A6 | 1.76977773063351 | 0.987 | 0.413 | 4.26336746617715E-225 | UP-regulation |
| TET2 | 1.76854344976039 | 0.871 | 0.337 | 2.67561554478426E-192 | UP-regulation |
| FCN1 | 1.76826861032803 | 0.933 | 0.213 | 2.34123181693778E-262 | UP-regulation |
| C5AR1 | 1.76425156918992 | 0.71 | 0.123 | 6.51664555800933E-245 | UP-regulation |
| FCAR | 1.76355490631729 | 0.631 | 0.1 | 3.35459312110928E-231 | UP-regulation |
| HCK | 1.7462643241443 | 0.657 | 0.113 | 9.33563461273418E-229 | UP-regulation |
| TREM1 | 1.74253189726651 | 0.638 | 0.09 | 8.05475153308243E-253 | UP-regulation |
| BASP1 | 1.73803832877822 | 0.769 | 0.268 | 2.92465355731042E-165 | UP-regulation |
| SAT1 | 1.72987844440182 | 0.978 | 0.426 | 5.88048668641129E-201 | UP-regulation |
| TYROBP | 1.704491588883 | 0.966 | 0.28 | 1.49327596463009E-232 | UP-regulation |
| FNDC3B | 1.69744787361331 | 0.856 | 0.294 | 6.10030838220923E-197 | UP-regulation |
| SRGN | 1.69460914011407 | 0.987 | 0.579 | 1.57761307100672E-209 | UP-regulation |
| NCF1 | 1.67733086328945 | 0.683 | 0.132 | 1.09494769004667E-215 | UP-regulation |
| DOCK4 | 1.64388866608272 | 0.533 | 0.141 | 4.07686379486893E-121 | UP-regulation |
| EREG | 1.63754747106882 | 0.434 | 0.138 | 8.74716863494346E-77 | UP-regulation |
| S100A11 | 1.63226770213678 | 0.931 | 0.303 | 5.95828980799378E-214 | UP-regulation |
| C9orf72 | 1.63146682049853 | 0.692 | 0.177 | 4.5148491574326E-178 | UP-regulation |
| PELI1 | 1.62676153360701 | 0.623 | 0.214 | 7.9120632535597E-115 | UP-regulation |
| NEAT1 | 1.62354418466474 | 0.988 | 0.603 | 2.66534826855242E-205 | UP-regulation |
| UBAP1 | 1.61161926565943 | 0.868 | 0.354 | 3.07648454900532E-179 | UP-regulation |
| NKAIN2 | -2.98774113673261 | 0.028 | 0.479 | 2.15533206871921E-87 | DOWN-regulation |
| AL589693.1 | -2.64213686985035 | 0.027 | 0.355 | 7.56770380928835E-54 | DOWN-regulation |
| SNHG29 | -2.40295323521026 | 0.419 | 0.779 | 1.29548394646508E-130 | DOWN-regulation |
| INPP4B | -2.32478648652473 | 0.01 | 0.436 | 3.04619741918835E-79 | DOWN-regulation |
| LRBA | -2.31278944165803 | 0.141 | 0.587 | 1.02355511838074E-89 | DOWN-regulation |
| HSP90AB1 | -2.16413303316406 | 0.415 | 0.797 | 1.41764764332823E-135 | DOWN-regulation |
| CASC15 | -2.16315591073121 | 0.027 | 0.423 | 1.99959675193106E-71 | DOWN-regulation |
| NPM1 | -2.13466356454218 | 0.332 | 0.744 | 2.26790080721906E-123 | DOWN-regulation |
| AFF3 | -2.11312695269977 | 0.049 | 0.424 | 7.42491029181398E-64 | DOWN-regulation |
| RPS4X | -2.05855684858028 | 0.918 | 0.946 | 2.88556827787958E-177 | DOWN-regulation |
| RPS5 | -2.04611437219079 | 0.662 | 0.882 | 1.09185940223994E-158 | DOWN-regulation |
| RPL3 | -2.02206640733661 | 0.802 | 0.918 | 1.03753428558708E-174 | DOWN-regulation |
| LDHB | -1.99088839369867 | 0.097 | 0.562 | 6.40761077129811E-93 | DOWN-regulation |
| MSI2 | -1.98448352776143 | 0.162 | 0.543 | 9.70875345480151E-70 | DOWN-regulation |
| CDK6 | -1.96360295114147 | 0.035 | 0.455 | 2.7907275587793E-76 | DOWN-regulation |
| BACH2 | -1.91311152997411 | 0.013 | 0.305 | 1.03503444497616E-45 | DOWN-regulation |
| STMN1 | -1.90944591373069 | 0.018 | 0.434 | 8.57451523873223E-76 | DOWN-regulation |
| CD69 | -1.90338015085367 | 0.01 | 0.407 | 5.26486083171052E-71 | DOWN-regulation |
| RPSA | -1.89446396454926 | 0.608 | 0.86 | 1.10471493369142E-145 | DOWN-regulation |
| RPL10A | -1.86469281980669 | 0.668 | 0.874 | 3.61934668297956E-148 | DOWN-regulation |
| SPINK2 | -1.83610311922913 | 0.007 | 0.395 | 2.0843395659266E-69 | DOWN-regulation |
| EEF1B2 | -1.83402739176031 | 0.608 | 0.852 | 1.02264805070781E-138 | DOWN-regulation |
| RPS3 | -1.83297986115872 | 0.911 | 0.94 | 2.81335562647952E-170 | DOWN-regulation |
| CALN1 | -1.81407252191021 | 0.012 | 0.342 | 7.56159616427332E-55 | DOWN-regulation |
| LTB | -1.78608323106045 | 0.06 | 0.279 | 3.57527181913935E-27 | DOWN-regulation |
| RPS18 | -1.73899371070861 | 0.884 | 0.938 | 2.74986680380898E-165 | DOWN-regulation |
| HINT1 | -1.68779742694078 | 0.4 | 0.733 | 3.29685176898993E-96 | DOWN-regulation |
| SPTBN1 | -1.6519044479201 | 0.069 | 0.424 | 8.72540522854381E-58 | DOWN-regulation |
| TOX | -1.62231817179797 | 0.01 | 0.335 | 2.59672709240013E-53 | DOWN-regulation |
| ERG | -1.62024463337364 | 0.007 | 0.33 | 3.05489026228365E-53 | DOWN-regulation |
| LAPTM4B | -1.61008869794575 | 0.023 | 0.388 | 1.24497773088135E-61 | DOWN-regulation |
| TCF4 | -1.58798866708642 | 0.03 | 0.357 | 6.02798758874296E-52 | DOWN-regulation |
| C1QTNF4 | -1.58687829593778 | 0.005 | 0.334 | 1.02706757881434E-54 | DOWN-regulation |
| RPLP0 | -1.57896148570947 | 0.625 | 0.844 | 1.54545377475246E-119 | DOWN-regulation |
| PRSS57 | -1.57719749947066 | 0.008 | 0.356 | 7.32181649349785E-59 | DOWN-regulation |
| CRHBP | -1.57100783760823 | 0.007 | 0.327 | 1.35040032650452E-52 | DOWN-regulation |
| RPL5 | -1.56358806650747 | 0.831 | 0.91 | 2.20889026129587E-146 | DOWN-regulation |
| SKAP1 | -1.55182519094543 | 0.005 | 0.288 | 1.52941434918385E-44 | DOWN-regulation |
| RPS23 | -1.5423341036599 | 0.881 | 0.927 | 1.87579392290477E-152 | DOWN-regulation |
| GAS5 | -1.5406905500146 | 0.281 | 0.627 | 7.37119044430191E-70 | DOWN-regulation |
| CHRM3 | -1.53419434032873 | 0.012 | 0.312 | 1.26332002305049E-47 | DOWN-regulation |
| HNRNPA1 | -1.53244727120455 | 0.404 | 0.728 | 9.7022411510503E-85 | DOWN-regulation |
| RPS12 | -1.52449312629846 | 0.926 | 0.952 | 3.73073201572905E-165 | DOWN-regulation |
| MLLT3 | -1.5088346357884 | 0.02 | 0.349 | 2.88845570978852E-53 | DOWN-regulation |
| MSRB3 | -1.4897404378587 | 0.01 | 0.326 | 2.64948096204209E-51 | DOWN-regulation |
| RPS2 | -1.47933591363713 | 0.908 | 0.947 | 1.17826239504411E-151 | DOWN-regulation |
| NRIP1 | -1.47359439118022 | 0.327 | 0.568 | 2.86710471506617E-39 | DOWN-regulation |
| SSBP2 | -1.47135853266197 | 0.27 | 0.559 | 4.46121924655656E-45 | DOWN-regulation |
| RERE | -1.46238979785576 | 0.248 | 0.556 | 4.00923009488684E-48 | DOWN-regulation |
| FAM30A | -1.45742474607723 | 0.007 | 0.301 | 5.39927606275003E-47 | DOWN-regulation |

**Table S11.** Top 50 up and down-regulated genes in cluster 3 in CD34+Lin-CD45+ cells.

| **gene** | **avg_log2FC** | **pct.1** | **pct.2** | **p_val_adj** | **expression** |
| --- | --- | --- | --- | --- | --- |
| S100A9 | 2.2954029949274 | 0.973 | 0.369 | 3.03707882446216E-203 | UP-regulation |
| S100A8 | 2.20638660449147 | 0.938 | 0.292 | 3.14835648619225E-209 | UP-regulation |
| LYZ | 2.1787643890833 | 0.921 | 0.285 | 7.16011276666996E-205 | UP-regulation |
| S100A6 | 2.02920859284344 | 0.938 | 0.434 | 2.38688920939171E-173 | UP-regulation |
| S100A12 | 1.94971185276196 | 0.593 | 0.18 | 1.23633272737386E-107 | UP-regulation |
| FCN1 | 1.92280497735889 | 0.84 | 0.242 | 8.48536625211953E-178 | UP-regulation |
| CST3 | 1.91882999613993 | 0.88 | 0.363 | 4.34181196099181E-157 | UP-regulation |
| S100A4 | 1.91662783047929 | 0.981 | 0.655 | 1.28031709509761E-178 | UP-regulation |
| AC020656.1 | 1.87143636589036 | 0.475 | 0.156 | 7.06338950707443E-79 | UP-regulation |
| S100A11 | 1.84753038569579 | 0.801 | 0.334 | 1.70695736824797E-127 | UP-regulation |
| TYROBP | 1.84631842926445 | 0.888 | 0.307 | 3.67835011773564E-167 | UP-regulation |
| G0S2 | 1.84447485480537 | 0.66 | 0.218 | 3.7380696654791E-104 | UP-regulation |
| SAT1 | 1.79613923750323 | 0.913 | 0.448 | 8.26757354387031E-132 | UP-regulation |
| CD14 | 1.74407838734033 | 0.444 | 0.131 | 5.8955811832732E-78 | UP-regulation |
| LGALS2 | 1.73687018086459 | 0.579 | 0.191 | 2.03684856085866E-90 | UP-regulation |
| IFI30 | 1.71933007260902 | 0.647 | 0.216 | 4.8055472251025E-108 | UP-regulation |
| MNDA | 1.71602441988297 | 0.722 | 0.228 | 7.01486986979281E-128 | UP-regulation |
| LGALS1 | 1.70696625268306 | 0.71 | 0.275 | 2.33983828625476E-103 | UP-regulation |
| S100A10 | 1.66425846608218 | 0.745 | 0.336 | 6.44723743518408E-101 | UP-regulation |
| FCER1G | 1.65091635669038 | 0.654 | 0.271 | 9.69879850847715E-88 | UP-regulation |
| CXCL8 | 1.62985853585554 | 0.741 | 0.255 | 9.64568351170237E-119 | UP-regulation |
| CTSS | 1.60238537233762 | 0.89 | 0.421 | 5.9194144414815E-126 | UP-regulation |
| VCAN | 1.5936129110219 | 0.797 | 0.237 | 1.50164619910899E-134 | UP-regulation |
| CFD | 1.58940372470027 | 0.512 | 0.182 | 6.98678763047446E-73 | UP-regulation |
| FTL | 1.58675022203711 | 1 | 0.957 | 8.81082185126575E-173 | UP-regulation |
| PLAUR | 1.56052072784795 | 0.755 | 0.269 | 3.14841042342679E-115 | UP-regulation |
| BCL2A1 | 1.53134257002542 | 0.452 | 0.174 | 5.21057147601638E-56 | UP-regulation |
| FTH1 | 1.5196547631135 | 0.998 | 0.961 | 5.73983865257819E-154 | UP-regulation |
| NAMPT | 1.50893723488236 | 0.965 | 0.441 | 1.44181728879464E-139 | UP-regulation |
| CRIP1 | 1.48643702122035 | 0.641 | 0.309 | 5.7865783485121E-64 | UP-regulation |
| TYMP | 1.48501548576337 | 0.492 | 0.186 | 7.83155761503336E-63 | UP-regulation |
| IL1B | 1.4752513400477 | 0.481 | 0.226 | 2.61986890175949E-42 | UP-regulation |
| TSPO | 1.46902054602441 | 0.763 | 0.498 | 7.00347036144331E-78 | UP-regulation |
| NFKBIA | 1.46530379302376 | 0.813 | 0.509 | 1.69666220270029E-84 | UP-regulation |
| COTL1 | 1.45695293409418 | 0.618 | 0.293 | 6.20896396905517E-65 | UP-regulation |
| LST1 | 1.43146707062781 | 0.639 | 0.375 | 4.70662920903646E-55 | UP-regulation |
| SOD2 | 1.42756135899918 | 0.834 | 0.622 | 1.13347584098548E-78 | UP-regulation |
| FGD4 | 1.40479895056871 | 0.645 | 0.278 | 4.37135607405191E-70 | UP-regulation |
| LGALS3 | 1.40049757604067 | 0.369 | 0.131 | 1.03119313480213E-46 | UP-regulation |
| NCF1 | 1.38122417470309 | 0.432 | 0.174 | 6.35764121771366E-46 | UP-regulation |
| ACSL1 | 1.37235161986692 | 0.587 | 0.295 | 4.03503625288332E-50 | UP-regulation |
| CLEC7A | 1.35568036535864 | 0.471 | 0.186 | 2.55119384501079E-53 | UP-regulation |
| SLC11A1 | 1.34925472306158 | 0.517 | 0.2 | 2.67559493324953E-58 | UP-regulation |
| ANXA2 | 1.34706835357647 | 0.45 | 0.168 | 7.79708897188529E-56 | UP-regulation |
| STXBP2 | 1.34164180259835 | 0.432 | 0.238 | 4.29729419668911E-29 | UP-regulation |
| CYBA | 1.31469307000377 | 0.832 | 0.637 | 9.16341565747092E-74 | UP-regulation |
| NCF2 | 1.31308809763296 | 0.425 | 0.189 | 4.01141604827931E-39 | UP-regulation |
| SH3BGRL3 | 1.30797479262656 | 0.89 | 0.733 | 4.31388458097088E-97 | UP-regulation |
| ANXA1 | 1.30090428733354 | 0.668 | 0.346 | 4.76107301364186E-58 | UP-regulation |
| SERPINA1 | 1.30031211475365 | 0.351 | 0.133 | 3.50909027803569E-38 | UP-regulation |
| HBB | -4.96388257617296 | 0.052 | 0.135 | 0.0104967533918296 | DOWN-regulation |
| HBG1 | -4.20064029976695 | 0.027 | 0.1 | 0.00605134328823112 | DOWN-regulation |
| NKAIN2 | -2.86685373879161 | 0.027 | 0.468 | 1.56330636618102E-67 | DOWN-regulation |
| AL589693.1 | -2.57373517826985 | 0.019 | 0.347 | 3.72942427183366E-43 | DOWN-regulation |
| LRBA | -2.24878434574584 | 0.098 | 0.58 | 2.7081184477927E-75 | DOWN-regulation |
| INPP4B | -2.16510313781785 | 0.021 | 0.424 | 3.45909156451534E-58 | DOWN-regulation |
| CASC15 | -2.08836029070833 | 0.017 | 0.414 | 6.85141266933821E-57 | DOWN-regulation |
| AFF3 | -2.01209309056075 | 0.033 | 0.416 | 3.95393145965712E-52 | DOWN-regulation |
| SNHG29 | -1.81996168392469 | 0.382 | 0.773 | 2.70227383622989E-78 | DOWN-regulation |
| STMN1 | -1.79509894114857 | 0.021 | 0.423 | 4.08479030231416E-57 | DOWN-regulation |
| NPM1 | -1.79442973588496 | 0.253 | 0.742 | 6.86297921582252E-87 | DOWN-regulation |
| MSI2 | -1.77116107668518 | 0.122 | 0.537 | 1.66091990952218E-55 | DOWN-regulation |
| CDK6 | -1.77033922762777 | 0.035 | 0.444 | 1.04815837763561E-56 | DOWN-regulation |
| SPINK2 | -1.76136262568142 | 0.01 | 0.385 | 1.47848742023154E-52 | DOWN-regulation |
| CD69 | -1.74643589596844 | 0.023 | 0.395 | 8.26371533539568E-51 | DOWN-regulation |
| CALN1 | -1.6981884872256 | 0.017 | 0.333 | 1.13495323580109E-40 | DOWN-regulation |
| BACH2 | -1.69177093600989 | 0.027 | 0.296 | 4.28589380318145E-31 | DOWN-regulation |
| HSP90AB1 | -1.66716206771724 | 0.361 | 0.793 | 1.81995430083628E-83 | DOWN-regulation |
| NRIP1 | -1.63670137685504 | 0.168 | 0.579 | 3.55202462646959E-54 | DOWN-regulation |
| LDHB | -1.62676656833427 | 0.095 | 0.55 | 5.15928540358663E-64 | DOWN-regulation |
| TOX | -1.6254688097742 | 0.002 | 0.327 | 1.88731319197317E-43 | DOWN-regulation |
| GAS5 | -1.59920710718619 | 0.164 | 0.631 | 4.66181215450893E-69 | DOWN-regulation |
| C1QTNF4 | -1.57338009248229 | 0.004 | 0.326 | 8.1189285807471E-43 | DOWN-regulation |
| PRSS57 | -1.54257077945062 | 0.008 | 0.347 | 1.0625333472663E-45 | DOWN-regulation |
| TCF4 | -1.5261199167497 | 0.019 | 0.35 | 1.54308200858097E-42 | DOWN-regulation |
| ERG | -1.52257076176909 | 0.01 | 0.321 | 6.84640521141748E-40 | DOWN-regulation |
| SPTBN1 | -1.49472663873845 | 0.062 | 0.415 | 4.39293953999984E-44 | DOWN-regulation |
| LTB | -1.46997943480174 | 0.077 | 0.271 | 3.32208496425971E-16 | DOWN-regulation |
| RPS4X | -1.46510567928928 | 0.861 | 0.952 | 1.82155367673139E-89 | DOWN-regulation |
| RNF220 | -1.46307088945885 | 0.044 | 0.372 | 5.77098721359015E-40 | DOWN-regulation |
| CRHBP | -1.45546343020915 | 0.012 | 0.318 | 1.17640584909716E-38 | DOWN-regulation |
| CHRM3 | -1.44858160847617 | 0.015 | 0.304 | 9.87249994692535E-36 | DOWN-regulation |
| LAPTM4B | -1.43694856156433 | 0.027 | 0.378 | 4.0015697316012E-45 | DOWN-regulation |
| RPL3 | -1.43254319464848 | 0.763 | 0.919 | 1.23623118504299E-90 | DOWN-regulation |
| MLLT3 | -1.42323617317102 | 0.019 | 0.34 | 4.04348922844604E-41 | DOWN-regulation |
| MSRB3 | -1.42304977532412 | 0.008 | 0.318 | 9.7360652980636E-40 | DOWN-regulation |
| KCNQ5 | -1.40898826271038 | 0.01 | 0.275 | 4.33751532473816E-32 | DOWN-regulation |
| RERE | -1.39842866643429 | 0.162 | 0.557 | 3.62026671214076E-47 | DOWN-regulation |
| SMYD3 | -1.39014337689771 | 0.077 | 0.441 | 3.5047522666082E-44 | DOWN-regulation |
| FAM30A | -1.38622711458666 | 0.006 | 0.293 | 5.10610015889902E-36 | DOWN-regulation |
| HNRNPA1 | -1.38109385649859 | 0.317 | 0.729 | 1.74925140314723E-61 | DOWN-regulation |
| CD109 | -1.37109095910072 | 0.01 | 0.301 | 4.36382721696275E-36 | DOWN-regulation |
| ZNF521 | -1.36506511921724 | 0.002 | 0.3 | 1.72792058439527E-38 | DOWN-regulation |
| ST8SIA6 | -1.35696715635725 | 0.006 | 0.288 | 1.93719987703052E-35 | DOWN-regulation |
| PPP1R16B | -1.35391650577497 | 0.044 | 0.365 | 1.00479403095045E-37 | DOWN-regulation |
| SKAP1 | -1.3431868457067 | 0.031 | 0.278 | 2.85426290335262E-27 | DOWN-regulation |
| RPSA | -1.33791949495553 | 0.546 | 0.861 | 1.30291284857999E-73 | DOWN-regulation |
| SSBP2 | -1.33654567016611 | 0.17 | 0.562 | 1.88157088570337E-45 | DOWN-regulation |
| HINT1 | -1.31298672323197 | 0.326 | 0.733 | 3.2713475577629E-61 | DOWN-regulation |
| AUTS2 | -1.30648634428569 | 0.079 | 0.435 | 3.87797207238769E-41 | DOWN-regulation |

**Table S12.** Top 50 up and down-regulated genes in cluster 1 in CD133+Lin-CD45+ cells.

| **gene** | **avg_log2FC** | **pct.1** | **pct.2** | **p_val_adj** | **expression** |
| --- | --- | --- | --- | --- | --- |
| AC025419.1 | 6.91002108393799 | 0.015 | 0 | 0.0000017601082779272 | UP-regulation |
| AC011921.1 | 6.72815818459605 | 0.019 | 0 | 4.74037993984517E-09 | UP-regulation |
| AL355432.1 | 6.54509614868911 | 0.015 | 0 | 0.0000017601082779272 | UP-regulation |
| AL354936.1 | 6.3757198671389 | 0.012 | 0 | 0.0000921142629125372 | UP-regulation |
| NAV2 | 6.27702066354103 | 0.011 | 0 | 0.000670586956723738 | UP-regulation |
| AC007422.1 | 6.16315329839908 | 0.011 | 0 | 0.000670586956723738 | UP-regulation |
| HAS2 | 6.15507748662545 | 0.012 | 0 | 0.0000921142629125372 | UP-regulation |
| KRBOX1 | 6.12255513020992 | 0.012 | 0 | 0.0000921142629125372 | UP-regulation |
| TPTE2 | 6.00554079693967 | 0.011 | 0 | 0.000670586956723738 | UP-regulation |
| C6orf141 | 6.00115598431785 | 0.011 | 0 | 0.000670586956723738 | UP-regulation |
| CFAP69 | 6.00058915086892 | 0.011 | 0 | 0.000670586956723738 | UP-regulation |
| AC090192.2 | 5.908184981345 | 0.011 | 0 | 0.000670586956723738 | UP-regulation |
| LINC02839 | 5.75580413730275 | 0.048 | 0.001 | 2.46788824014963E-24 | UP-regulation |
| CCDC196 | 5.33200276388727 | 0.028 | 0 | 8.54118584684667E-14 | UP-regulation |
| AC021504.1 | 5.2904433476463 | 0.02 | 0.001 | 1.20598672622218E-07 | UP-regulation |
| AC099063.4 | 5.28750488015836 | 0.016 | 0 | 3.75754385370489E-06 | UP-regulation |
| AC006008.1 | 5.16599195657384 | 0.017 | 0 | 5.45023144858449E-07 | UP-regulation |
| RNF150 | 5.06980742945294 | 0.149 | 0.003 | 1.31623188152075E-81 | UP-regulation |
| AC009275.1 | 5.05523600209328 | 0.011 | 0 | 0.00962929148169443 | UP-regulation |
| AC092058.1 | 5.00402945702358 | 0.016 | 0 | 3.79520393576277E-06 | UP-regulation |
| PRSS51 | 4.99113747850662 | 0.016 | 0 | 3.85238653735255E-06 | UP-regulation |
| AC245123.1 | 4.97356657321579 | 0.016 | 0 | 3.84279798481787E-06 | UP-regulation |
| HSPA12A | 4.91792471230818 | 0.022 | 0 | 1.53570757012627E-09 | UP-regulation |
| PDE10A | 4.83139217140191 | 0.079 | 0.002 | 1.33643366025513E-39 | UP-regulation |
| CFAP57 | 4.81877782870311 | 0.024 | 0.001 | 3.73561988569916E-10 | UP-regulation |
| AC009121.3 | 4.7992860909545 | 0.012 | 0 | 0.00134841416753435 | UP-regulation |
| FAM163A | 4.76879534966391 | 0.02 | 0.001 | 1.2433397107394E-07 | UP-regulation |
| AL590428.1 | 4.76494137326663 | 0.013 | 0 | 0.000192074752238187 | UP-regulation |
| AC103923.1 | 4.75835746993885 | 0.016 | 0 | 3.86199750624594E-06 | UP-regulation |
| AL358944.1 | 4.75497477566056 | 0.012 | 0 | 0.00135828445805473 | UP-regulation |
| AC012404.1 | 4.71707151379989 | 0.047 | 0.001 | 2.04245876801159E-22 | UP-regulation |
| NKX2-3 | 4.68906060386272 | 0.016 | 0 | 3.86199846658048E-06 | UP-regulation |
| C3orf22 | 4.66949487476936 | 0.013 | 0 | 0.000193020006475632 | UP-regulation |
| AC011139.1 | 4.64710204533592 | 0.022 | 0 | 1.53180860842051E-09 | UP-regulation |
| MMP16 | 4.63021186268769 | 0.07 | 0.003 | 6.06036917248344E-32 | UP-regulation |
| PRR15 | 4.62223579367383 | 0.013 | 0 | 0.000193494328121885 | UP-regulation |
| NFATC4 | 4.61386199111088 | 0.013 | 0 | 0.000193969782944018 | UP-regulation |
| AC015908.2 | 4.5960650597422 | 0.017 | 0.001 | 5.78430608943301E-06 | UP-regulation |
| AF127577.1 | 4.50054358359445 | 0.012 | 0 | 0.00137155380738382 | UP-regulation |
| AL359715.1 | 4.468112629382 | 0.013 | 0 | 0.00019444637356751 | UP-regulation |
| AC007091.1 | 4.45852583845914 | 0.015 | 0.001 | 0.000269232153672064 | UP-regulation |
| AC006511.4 | 4.44962943448527 | 0.015 | 0.001 | 0.000264454688427012 | UP-regulation |
| AC010745.5 | 4.42420636776197 | 0.023 | 0.001 | 2.4242779956667E-08 | UP-regulation |
| WIPF3 | 4.41313045646116 | 0.03 | 0.001 | 1.49700573251238E-11 | UP-regulation |
| AC107021.1 | 4.40964085174209 | 0.034 | 0.001 | 4.99903565522464E-14 | UP-regulation |
| KIAA1211 | 4.35836494105919 | 0.421 | 0.027 | 1.21815385313395E-205 | UP-regulation |
| SPANXA2-OT1 | 4.35744120582366 | 0.043 | 0.002 | 6.64312916371452E-19 | UP-regulation |
| ARHGAP20 | 4.31109265999983 | 0.141 | 0.008 | 6.1218458912954E-64 | UP-regulation |
| ARNTL2-AS1 | 4.30120457381603 | 0.098 | 0.005 | 1.01934494268065E-45 | UP-regulation |
| PREX2 | 4.27931724165436 | 0.456 | 0.025 | 5.78073394330974E-233 | UP-regulation |
| ALDH1A2 | -9.8243815251627 | 0 | 0.125 | 8.8322610033751E-20 | DOWN-regulation |
| AL049651.1 | -9.36405618047712 | 0 | 0.052 | 4.11811929900981E-06 | DOWN-regulation |
| CLEC4E | -9.3389798382179 | 0 | 0.142 | 3.66860590954121E-23 | DOWN-regulation |
| CSTA | -9.25558452706264 | 0.001 | 0.33 | 3.34158719548678E-67 | DOWN-regulation |
| AC005050.3 | -9.16715742766676 | 0.003 | 0.155 | 6.99979731638221E-25 | DOWN-regulation |
| LGALS2 | -9.14903554873962 | 0.004 | 0.378 | 4.4496817240021E-80 | DOWN-regulation |
| TLR4 | -9.03882215453645 | 0 | 0.121 | 6.00005173207754E-19 | DOWN-regulation |
| AHSP | -8.90784109306131 | 0.001 | 0.039 | 0.00300663903992117 | DOWN-regulation |
| PCED1B-AS1 | -8.89705100425945 | 0 | 0.104 | 1.13458746245015E-15 | DOWN-regulation |
| MMP9 | -8.81965876962512 | 0 | 0.042 | 0.000267734147033059 | DOWN-regulation |
| CD96 | -8.71532477486841 | 0 | 0.084 | 5.98529569182666E-12 | DOWN-regulation |
| PID1 | -8.7048782650972 | 0.001 | 0.253 | 1.93090450143948E-47 | DOWN-regulation |
| TNFAIP6 | -8.67047393804712 | 0 | 0.053 | 3.08263314389714E-06 | DOWN-regulation |
| VCAN | -8.51746508147921 | 0.012 | 0.449 | 5.79574236658678E-100 | DOWN-regulation |
| S100A12 | -8.5159039695024 | 0.022 | 0.487 | 5.3941742086243E-110 | DOWN-regulation |
| CSF1R | -8.46218821367487 | 0 | 0.115 | 8.87189176282018E-18 | DOWN-regulation |
| RBP7 | -8.46185517352047 | 0.001 | 0.225 | 9.84967191431608E-41 | DOWN-regulation |
| GZMA | -8.4294700191083 | 0 | 0.049 | 0.0000201694484566384 | DOWN-regulation |
| LIPN | -8.4191300760929 | 0 | 0.089 | 7.183528407597E-13 | DOWN-regulation |
| CEACAM3 | -8.41398897532908 | 0 | 0.031 | 0.0295662407721928 | DOWN-regulation |
| CXCR2 | -8.40042247340475 | 0 | 0.038 | 0.00171642396105975 | DOWN-regulation |
| VNN3 | -8.38721009551525 | 0 | 0.061 | 1.08368420493862E-07 | DOWN-regulation |
| S100P | -8.31855118426277 | 0.001 | 0.095 | 1.6320498787359E-13 | DOWN-regulation |
| IL1R2 | -8.29333075267671 | 0.001 | 0.062 | 2.14741953265027E-07 | DOWN-regulation |
| FCGR2A | -8.29158909138428 | 0.001 | 0.2 | 3.71384121217386E-35 | DOWN-regulation |
| G0S2 | -8.26790785439473 | 0.009 | 0.459 | 9.78951641896512E-104 | DOWN-regulation |
| DYSF | -8.23810301516679 | 0.001 | 0.213 | 5.10137859097408E-38 | DOWN-regulation |
| CD14 | -8.23735557850758 | 0.003 | 0.332 | 1.78627829154411E-67 | DOWN-regulation |
| CCL3 | -8.23337373621829 | 0 | 0.047 | 0.0000478446691604233 | DOWN-regulation |
| GNLY | -8.2317853052097 | 0.004 | 0.048 | 0.000607891373733228 | DOWN-regulation |
| FCGR3B | -8.21121322684661 | 0.001 | 0.075 | 9.05868615895174E-10 | DOWN-regulation |
| HP | -8.19489799744897 | 0 | 0.049 | 0.000020169448008318 | DOWN-regulation |
| LINC01506 | -8.17362280978193 | 0 | 0.043 | 0.000232005263420865 | DOWN-regulation |
| LGALS3 | -8.15509896005196 | 0.003 | 0.308 | 5.42898263569362E-61 | DOWN-regulation |
| S100A8 | -8.14955287858438 | 0.117 | 0.662 | 5.18771032173817E-159 | DOWN-regulation |
| THBS1 | -8.09009176090544 | 0.005 | 0.317 | 1.4494172482077E-62 | DOWN-regulation |
| THEMIS | -8.03937115620157 | 0 | 0.059 | 2.60371135665403E-07 | DOWN-regulation |
| SIRPB1 | -8.01403914967909 | 0 | 0.07 | 2.73920194511441E-09 | DOWN-regulation |
| HCAR3 | -8.01377198490801 | 0 | 0.05 | 0.0000130859869434318 | DOWN-regulation |
| SMIM25 | -7.99367334016681 | 0.001 | 0.17 | 1.19907448870708E-28 | DOWN-regulation |
| HMOX1 | -7.99189342328715 | 0 | 0.078 | 1.03886236523744E-10 | DOWN-regulation |
| IL7R | -7.98453037254158 | 0.003 | 0.115 | 5.82894908053559E-17 | DOWN-regulation |
| GZMM | -7.98292505596379 | 0 | 0.058 | 3.48552974232789E-07 | DOWN-regulation |
| PROK2 | -7.96583973939485 | 0.003 | 0.122 | 2.83885438417532E-18 | DOWN-regulation |
| S100A9 | -7.93638496830907 | 0.172 | 0.675 | 2.469073284199E-154 | DOWN-regulation |
| CLIC3 | -7.92701474706827 | 0 | 0.034 | 0.00948307848858249 | DOWN-regulation |
| KYNU | -7.89529257282779 | 0.004 | 0.319 | 2.35014069684244E-63 | DOWN-regulation |
| CYBB | -7.87890044194364 | 0.005 | 0.387 | 2.54160663137747E-82 | DOWN-regulation |
| FOLR3 | -7.8433983249987 | 0 | 0.051 | 7.34430492727667E-06 | DOWN-regulation |
| MNDA | -7.79985129390266 | 0.011 | 0.555 | 4.89977772835969E-138 | DOWN-regulation |

**Table S13.** Top 50 up and down-regulated genes in cluster 3 in CD133+Lin-CD45+ cells.

| **gene** | **avg_log2FC** | **pct.1** | **pct.2** | **p_val_adj** | **expression** |
| --- | --- | --- | --- | --- | --- |
| NELL2 | 10.3635575234476 | 0.327 | 0.001 | 2.10917293937052E-232 | UP-regulation |
| AC010275.1 | 10.0862908142339 | 0.091 | 0 | 2.12949226896711E-62 | UP-regulation |
| LINC02446 | 10.0161179979116 | 0.187 | 0.001 | 5.92492028267654E-128 | UP-regulation |
| CD8B | 9.7983977347765 | 0.155 | 0 | 1.05346340758349E-106 | UP-regulation |
| THEMIS | 9.77415314033715 | 0.485 | 0.001 | 0 | UP-regulation |
| CCR4 | 9.27495248773252 | 0.053 | 0 | 5.19499698245586E-35 | UP-regulation |
| CAMK4 | 9.22772971252526 | 0.857 | 0.004 | 0 | UP-regulation |
| FCGBP | 9.1284235019444 | 0.053 | 0 | 5.19499698245586E-35 | UP-regulation |
| AXIN2 | 8.74724409473565 | 0.061 | 0 | 6.0860535162574E-39 | UP-regulation |
| AL163932.1 | 8.53397986869204 | 0.073 | 0 | 2.36627200187579E-47 | UP-regulation |
| AC009041.1 | 8.25570310716284 | 0.023 | 0 | 5.39857310167358E-14 | UP-regulation |
| CLDN1 | 8.13079761891108 | 0.026 | 0 | 4.24756626713226E-16 | UP-regulation |
| UBASH3A | 7.99158699955901 | 0.085 | 0 | 9.20033870315664E-56 | UP-regulation |
| SUSD4 | 7.9810137499144 | 0.064 | 0 | 4.82395447522628E-41 | UP-regulation |
| LINC02273 | 7.89671634449022 | 0.091 | 0.001 | 9.88377937830073E-58 | UP-regulation |
| IL7R | 7.86933502102318 | 0.88 | 0.01 | 0 | UP-regulation |
| CA6 | 7.78594865559701 | 0.023 | 0 | 5.39857310167358E-14 | UP-regulation |
| AL109767.1 | 7.76224864711195 | 0.02 | 0 | 6.89475734381987E-12 | UP-regulation |
| LEF1 | 7.75899687761921 | 0.702 | 0.008 | 0 | UP-regulation |
| NLGN4X | 7.75719743058251 | 0.018 | 0 | 8.86827906108308E-10 | UP-regulation |
| CD27 | 7.64512421559995 | 0.474 | 0.004 | 0 | UP-regulation |
| ICOS | 7.47845113160352 | 0.351 | 0.004 | 1.25235941869595E-228 | UP-regulation |
| AC010468.2 | 7.44031580296519 | 0.012 | 0 | 0.0000152299513522765 | UP-regulation |
| RNF157 | 7.43767878359029 | 0.094 | 0.001 | 1.02501848005792E-57 | UP-regulation |
| TRAC | 7.38306315975074 | 0.55 | 0.004 | 0 | UP-regulation |
| CD3D | 7.34922750759883 | 0.83 | 0.006 | 0 | UP-regulation |
| ANKRD30BL | 7.33403792618816 | 0.012 | 0 | 0.0000152299513522765 | UP-regulation |
| AC107072.2 | 7.3240098704241 | 0.012 | 0 | 0.0000152299513522765 | UP-regulation |
| AQP3 | 7.31462662960963 | 0.14 | 0.002 | 2.67195403868089E-87 | UP-regulation |
| ASIC1 | 7.2747744001851 | 0.015 | 0 | 1.15276487112015E-07 | UP-regulation |
| TNIP3 | 7.26271177207722 | 0.035 | 0 | 4.36098198689172E-20 | UP-regulation |
| CD40LG | 7.20563607282242 | 0.064 | 0.001 | 7.44615841609041E-39 | UP-regulation |
| ECRG4 | 7.19863700253665 | 0.012 | 0 | 0.0000152299513522765 | UP-regulation |
| BCL11B | 7.17159082254523 | 0.807 | 0.009 | 0 | UP-regulation |
| LINC02536 | 7.08571080051035 | 0.012 | 0 | 0.0000152299513522765 | UP-regulation |
| AC005481.1 | 7.00209886115958 | 0.012 | 0 | 0.0000152299513522765 | UP-regulation |
| TAFA1 | 6.98402590567919 | 0.094 | 0.001 | 1.23018371937437E-57 | UP-regulation |
| TCEA3 | 6.95719271970213 | 0.053 | 0.001 | 1.6565804088206E-30 | UP-regulation |
| AC005842.1 | 6.86085194415547 | 0.085 | 0.001 | 1.87757483277163E-49 | UP-regulation |
| RAB25 | 6.85069819012705 | 0.026 | 0 | 7.88608832365817E-14 | UP-regulation |
| VSIG1 | 6.84829900908743 | 0.035 | 0 | 4.38468613306018E-20 | UP-regulation |
| LINC01550 | 6.83917519714247 | 0.102 | 0.001 | 5.97301832723117E-64 | UP-regulation |
| CD28 | 6.76296823160877 | 0.307 | 0.003 | 2.06404982361289E-199 | UP-regulation |
| EDAR | 6.74087956813524 | 0.029 | 0 | 6.50745710492364E-16 | UP-regulation |
| GPR171 | 6.66720909977914 | 0.047 | 0.001 | 1.70691889013298E-24 | UP-regulation |
| LRRN3 | 6.5878894830095 | 0.292 | 0.006 | 5.58241823234944E-169 | UP-regulation |
| PASK | 6.49721831833603 | 0.26 | 0.008 | 1.14953048282637E-139 | UP-regulation |
| CD3E | 6.42670223303408 | 0.632 | 0.011 | 0 | UP-regulation |
| GCSAM | 6.31955045050372 | 0.041 | 0.001 | 2.02298393053689E-20 | UP-regulation |
| CD3G | 6.16849760706478 | 0.69 | 0.009 | 0 | UP-regulation |
| CD14 | -9.44504017329886 | 0 | 0.292 | 4.37557546752262E-26 | DOWN-regulation |
| SERPINA1 | -9.31763897439485 | 0 | 0.275 | 5.61168970079121E-24 | DOWN-regulation |
| PID1 | -9.1816025027042 | 0 | 0.222 | 9.66814917710536E-18 | DOWN-regulation |
| EREG | -9.07481027228326 | 0 | 0.224 | 6.00692831025508E-18 | DOWN-regulation |
| PTGS2 | -9.03829182244937 | 0 | 0.181 | 2.7084475014191E-13 | DOWN-regulation |
| RTN1 | -8.59962737462428 | 0 | 0.197 | 5.55278247903935E-15 | DOWN-regulation |
| CD36 | -8.58736036371991 | 0 | 0.205 | 7.06862335212057E-16 | DOWN-regulation |
| CPVL | -8.25855203799629 | 0 | 0.193 | 1.59818722366147E-14 | DOWN-regulation |
| DUSP6 | -8.24480517640892 | 0 | 0.176 | 8.1492584720742E-13 | DOWN-regulation |
| TNFAIP2 | -8.10365522002579 | 0 | 0.176 | 8.14925819405529E-13 | DOWN-regulation |
| LILRB3 | -8.09401378332471 | 0 | 0.148 | 6.23020680436768E-10 | DOWN-regulation |
| S100A12 | -8.09050093728369 | 0.015 | 0.43 | 1.07724472352483E-43 | DOWN-regulation |
| KYNU | -7.91854763938584 | 0.003 | 0.28 | 3.77476537627536E-24 | DOWN-regulation |
| CLEC12A | -7.89902968128794 | 0 | 0.152 | 2.51080564540238E-10 | DOWN-regulation |
| CD86 | -7.82163208124846 | 0 | 0.144 | 1.53655028144538E-09 | DOWN-regulation |
| AC011472.2 | -7.81077771497679 | 0 | 0.102 | 0.0000146146102715113 | DOWN-regulation |
| LYZ | -7.69724184590208 | 0.018 | 0.464 | 4.43852512217E-49 | DOWN-regulation |
| MEF2C | -7.66992553093407 | 0.006 | 0.531 | 6.26362896843815E-63 | DOWN-regulation |
| HLA-DQA1 | -7.66819311439245 | 0 | 0.139 | 4.95764576558646E-09 | DOWN-regulation |
| MS4A7 | -7.61315536981122 | 0 | 0.125 | 9.80982668574855E-08 | DOWN-regulation |
| IL1RN | -7.59176843705773 | 0 | 0.094 | 0.0000776324997435314 | DOWN-regulation |
| PDE2A | -7.57637872678769 | 0 | 0.112 | 1.59453900860129E-06 | DOWN-regulation |
| LGALS2 | -7.54997684336898 | 0.006 | 0.332 | 2.1938403747883E-30 | DOWN-regulation |
| MEFV | -7.54494138542222 | 0 | 0.117 | 5.19426028056251E-07 | DOWN-regulation |
| FGL2 | -7.5332809416457 | 0.003 | 0.258 | 1.72576570429442E-21 | DOWN-regulation |
| TBC1D9 | -7.53230730321478 | 0 | 0.121 | 2.67339817980366E-07 | DOWN-regulation |
| AL163541.1 | -7.52896901966389 | 0 | 0.079 | 0.00160827319326343 | DOWN-regulation |
| AC007032.1 | -7.52271159870401 | 0 | 0.08 | 0.00141929614593215 | DOWN-regulation |
| LILRB2 | -7.50224970056859 | 0 | 0.121 | 2.50114287450022E-07 | DOWN-regulation |
| SMIM24 | -7.48893642801698 | 0 | 0.164 | 1.58344108977759E-11 | DOWN-regulation |
| CSF2RB | -7.46367554550768 | 0 | 0.066 | 0.0245054399236476 | DOWN-regulation |
| IL13RA1 | -7.43520702512433 | 0 | 0.107 | 5.17590465288915E-06 | DOWN-regulation |
| BST1 | -7.4282606100255 | 0 | 0.113 | 1.39810477516997E-06 | DOWN-regulation |
| PTX3 | -7.42126388824669 | 0 | 0.091 | 0.000146772573891442 | DOWN-regulation |
| CXCL2 | -7.40782323689538 | 0 | 0.097 | 0.0000436515037627062 | DOWN-regulation |
| BTK | -7.40704395250974 | 0 | 0.137 | 7.47773565284799E-09 | DOWN-regulation |
| CD34 | -7.37099154269178 | 0 | 0.157 | 8.13523059851042E-11 | DOWN-regulation |
| METRNL | -7.27949186770468 | 0 | 0.124 | 1.19951076709139E-07 | DOWN-regulation |
| SHTN1 | -7.27673445969671 | 0 | 0.122 | 2.04774636227866E-07 | DOWN-regulation |
| TFEC | -7.25516475150374 | 0 | 0.144 | 1.43378093346013E-09 | DOWN-regulation |
| LRP1 | -7.22604475439738 | 0 | 0.106 | 6.29182604414187E-06 | DOWN-regulation |
| SIRPA | -7.22273438871061 | 0 | 0.09 | 0.000177572956755494 | DOWN-regulation |
| SIRPB2 | -7.20860589290719 | 0 | 0.097 | 0.000040940325987653 | DOWN-regulation |
| DSC2 | -7.20642163504194 | 0 | 0.077 | 0.00264874763556118 | DOWN-regulation |
| G0S2 | -7.18837908634491 | 0.012 | 0.403 | 8.48980464422771E-40 | DOWN-regulation |
| IL1B | -7.17179664232989 | 0.006 | 0.364 | 9.64121411176553E-35 | DOWN-regulation |
| RIN2 | -7.16655181301728 | 0 | 0.114 | 1.00590050429525E-06 | DOWN-regulation |
| BMP6 | -7.15127490814672 | 0 | 0.103 | 0.0000105751062100348 | DOWN-regulation |
| CSF1R | -7.15101586943011 | 0 | 0.101 | 0.0000189211479345186 | DOWN-regulation |
| KCTD12 | -7.14332864811892 | 0 | 0.103 | 0.0000112826780514578 | DOWN-regulation |

**Table S14**. Top 50 up and down-regulated genes in cluster 4 in CD133+Lin-CD45+ cells.

| **gene** | **avg_log2FC** | **pct.1** | **pct.2** | **p_val_adj** | **expression** |
| --- | --- | --- | --- | --- | --- |
| CCDC184 | 6.26066256949787 | 0.01 | 0 | 0.000253850084259814 | UP-regulation |
| GLS2 | 5.94401818268671 | 0.01 | 0 | 0.000253850084259814 | UP-regulation |
| AL137802.2 | 5.33617464736857 | 0.017 | 0 | 8.58362893481311E-07 | UP-regulation |
| FMOD | 5.21029438823444 | 0.01 | 0 | 0.0367958429465703 | UP-regulation |
| ADRA2A | 5.15716069001743 | 0.026 | 0.001 | 3.19653256203749E-10 | UP-regulation |
| AC008946.1 | 4.89416506355022 | 0.01 | 0 | 0.0367958429465703 | UP-regulation |
| IRAIN | 4.83075539237279 | 0.01 | 0 | 0.0367958429465703 | UP-regulation |
| AC024361.2 | 4.67632260185644 | 0.01 | 0 | 0.0369801646885623 | UP-regulation |
| MIXL1 | 4.40328235023681 | 0.023 | 0.001 | 5.04274865540369E-08 | UP-regulation |
| AC022144.1 | 4.38155606346115 | 0.01 | 0 | 0.037165373844931 | UP-regulation |
| LINC02550 | 4.2860700066208 | 0.02 | 0.001 | 6.80594420781448E-06 | UP-regulation |
| AL603910.1 | 3.86567739031253 | 0.017 | 0.001 | 0.00777560942491462 | UP-regulation |
| AC026904.1 | 3.79001619462242 | 0.017 | 0.001 | 0.00082012117093939 | UP-regulation |
| PNMT | 3.71293707550315 | 0.036 | 0.004 | 2.67685055874354E-06 | UP-regulation |
| AC104958.2 | 3.68009456151628 | 0.023 | 0.002 | 0.0000105152164613471 | UP-regulation |
| LDOC1 | 3.59457012324175 | 0.271 | 0.025 | 1.10944103486586E-82 | UP-regulation |
| FOXB1 | 3.5365544785804 | 0.03 | 0.003 | 0.0000787855813409149 | UP-regulation |
| C3orf18 | 3.49420022653811 | 0.017 | 0.001 | 0.00805473538054454 | UP-regulation |
| ISYNA1 | 3.48885964831803 | 0.472 | 0.046 | 7.99703099358748E-144 | UP-regulation |
| AC108134.2 | 3.44661101827137 | 0.023 | 0.002 | 0.0000812185338734642 | UP-regulation |
| AC019077.1 | 3.40879483111394 | 0.017 | 0.002 | 0.0488354142471864 | UP-regulation |
| PLTP | 3.39547201296108 | 0.175 | 0.015 | 4.12180881061672E-52 | UP-regulation |
| AL355353.1 | 3.3424661548607 | 0.013 | 0.001 | 0.00632064278847254 | UP-regulation |
| FBLN1 | 3.33620073715398 | 0.023 | 0.002 | 0.0000823212446521998 | UP-regulation |
| AC007993.3 | 3.33137028333548 | 0.033 | 0.004 | 0.0000267829121268318 | UP-regulation |
| UCHL1 | 3.31379463702792 | 0.053 | 0.006 | 3.20156422832898E-11 | UP-regulation |
| DOK4 | 3.25781566478031 | 0.066 | 0.007 | 3.10657149836868E-15 | UP-regulation |
| P3H3 | 3.21637303178659 | 0.053 | 0.006 | 1.09742477253413E-10 | UP-regulation |
| LINC02767 | 3.17979190971129 | 0.046 | 0.005 | 8.22500314086387E-10 | UP-regulation |
| SLC22A17 | 3.17978401183604 | 0.063 | 0.007 | 2.95435273399722E-14 | UP-regulation |
| G6PC3 | 3.16862813290795 | 0.172 | 0.018 | 1.48328821245818E-45 | UP-regulation |
| EHD2 | 3.15731950573655 | 0.139 | 0.015 | 7.35479421138833E-34 | UP-regulation |
| AC017100.1 | 3.155587746225 | 0.05 | 0.006 | 3.66046559915563E-09 | UP-regulation |
| CYP2J2 | 3.12772944017917 | 0.017 | 0.001 | 0.00802639329786738 | UP-regulation |
| AL035701.1 | 3.1004329522529 | 0.03 | 0.004 | 0.000280243738123912 | UP-regulation |
| CENPVL3 | 3.09731483888789 | 0.026 | 0.002 | 0.0000468555618418792 | UP-regulation |
| HOXB5 | 3.06582968916194 | 0.03 | 0.004 | 0.00219212753146666 | UP-regulation |
| SERPING1 | 3.06544360000898 | 0.215 | 0.028 | 1.66618508418894E-47 | UP-regulation |
| SAPCD1 | 3.06306471787903 | 0.036 | 0.003 | 4.64547800178211E-08 | UP-regulation |
| AC004148.1 | 3.05685103255623 | 0.023 | 0.002 | 0.00212121747529515 | UP-regulation |
| ANKRD13B | 3.03361692643833 | 0.033 | 0.005 | 0.000268027725625793 | UP-regulation |
| ETNK2 | 3.02921840298946 | 0.046 | 0.006 | 1.0214062395184E-07 | UP-regulation |
| MFAP4 | 3.0050028923371 | 0.102 | 0.014 | 1.36977085899092E-19 | UP-regulation |
| CLDN10 | 3.00207871282525 | 0.139 | 0.018 | 4.38619676503071E-29 | UP-regulation |
| PDXP | 2.97742882376519 | 0.188 | 0.025 | 9.99498194660345E-40 | UP-regulation |
| PARP3 | 2.9668852703759 | 0.043 | 0.005 | 9.10543109658962E-09 | UP-regulation |
| B4GALT2 | 2.96234308817713 | 0.043 | 0.005 | 1.03665179358025E-07 | UP-regulation |
| GSTM5 | 2.9481103993461 | 0.102 | 0.013 | 2.10143929452337E-21 | UP-regulation |
| HOXA7 | 2.93771561558447 | 0.119 | 0.016 | 7.80211152340962E-24 | UP-regulation |
| NDN | 2.92821282026884 | 0.175 | 0.023 | 3.03671935528032E-38 | UP-regulation |
| CYBB | -9.55631911523793 | 0 | 0.336 | 3.99799271064587E-28 | DOWN-regulation |
| ARHGAP24 | -9.30916871224271 | 0 | 0.281 | 1.09424709648711E-21 | DOWN-regulation |
| VCAN | -9.18522753054262 | 0.007 | 0.391 | 2.25934439538906E-34 | DOWN-regulation |
| CSTA | -9.13990400914017 | 0 | 0.286 | 3.40597676267739E-22 | DOWN-regulation |
| SERPINA1 | -9.12580362953541 | 0 | 0.272 | 1.18498701843062E-20 | DOWN-regulation |
| LRRK2 | -9.09967937669181 | 0 | 0.251 | 2.0667119047382E-18 | DOWN-regulation |
| MS4A6A | -9.04608629260358 | 0 | 0.272 | 1.2787923032301E-20 | DOWN-regulation |
| FPR1 | -8.95290264133677 | 0 | 0.208 | 4.72847052562602E-14 | DOWN-regulation |
| S100A12 | -8.46864705346612 | 0.017 | 0.425 | 8.25124500977058E-38 | DOWN-regulation |
| GPR65 | -8.44798053749425 | 0 | 0.148 | 1.8053797426124E-08 | DOWN-regulation |
| ALOX5AP | -8.40734739432344 | 0 | 0.119 | 5.63369482558641E-06 | DOWN-regulation |
| AC099489.1 | -8.39186893672093 | 0 | 0.132 | 3.72124347007483E-07 | DOWN-regulation |
| FCN1 | -8.36365806555746 | 0.01 | 0.423 | 1.78366593266539E-38 | DOWN-regulation |
| SMIM25 | -8.35068046789068 | 0 | 0.148 | 1.69801952413933E-08 | DOWN-regulation |
| FCGR2A | -8.34327679117892 | 0 | 0.174 | 8.23455753393033E-11 | DOWN-regulation |
| AC020656.1 | -8.23649720593057 | 0.003 | 0.305 | 6.05500331917969E-24 | DOWN-regulation |
| HSD11B1-AS1 | -8.22237005331138 | 0 | 0.09 | 0.00127693426565582 | DOWN-regulation |
| IPCEF1 | -8.16655925259142 | 0 | 0.147 | 2.1695238303084E-08 | DOWN-regulation |
| KCNJ15 | -8.16367843287511 | 0 | 0.082 | 0.00571486764792331 | DOWN-regulation |
| CLEC7A | -8.06083646997022 | 0.003 | 0.35 | 2.22556052326378E-29 | DOWN-regulation |
| APOBEC3A | -8.03510351223763 | 0 | 0.159 | 1.71821751658141E-09 | DOWN-regulation |
| NEDD9 | -7.94190893192187 | 0.003 | 0.314 | 5.28173537641883E-25 | DOWN-regulation |
| THBS1 | -7.91837017147945 | 0.003 | 0.276 | 1.22572340046498E-20 | DOWN-regulation |
| AQP9 | -7.91039977775534 | 0.007 | 0.306 | 1.06535339881123E-23 | DOWN-regulation |
| LGALS2 | -7.85706678268843 | 0.007 | 0.328 | 3.30820849881338E-26 | DOWN-regulation |
| IL32 | -7.83812181956949 | 0 | 0.082 | 0.00484248524806496 | DOWN-regulation |
| CLEC4E | -7.8359721491137 | 0 | 0.123 | 2.48018554587975E-06 | DOWN-regulation |
| IGF2R | -7.80720987628526 | 0.003 | 0.226 | 2.04714267638591E-15 | DOWN-regulation |
| RAB31 | -7.68607137801663 | 0.007 | 0.388 | 7.59558613876455E-34 | DOWN-regulation |
| S100A9 | -7.6659592949533 | 0.175 | 0.608 | 1.95363228974364E-52 | DOWN-regulation |
| CD86 | -7.62979673638903 | 0 | 0.142 | 5.41390026005357E-08 | DOWN-regulation |
| RETN | -7.55483252967958 | 0.007 | 0.153 | 5.01157307521721E-08 | DOWN-regulation |
| SAMHD1 | -7.44060408059895 | 0.007 | 0.353 | 2.26055490016062E-29 | DOWN-regulation |
| FGL2 | -7.42358167827829 | 0.003 | 0.255 | 2.01084104336654E-18 | DOWN-regulation |
| CHST15 | -7.40204697113261 | 0 | 0.1 | 0.000188488976981657 | DOWN-regulation |
| DYSF | -7.37421399816236 | 0.003 | 0.185 | 2.24569076399424E-11 | DOWN-regulation |
| MEFV | -7.35310604056278 | 0 | 0.116 | 8.98046552151658E-06 | DOWN-regulation |
| PLD1 | -7.31945479856045 | 0 | 0.088 | 0.00188645860082241 | DOWN-regulation |
| CD3G | -7.22766956551905 | 0 | 0.08 | 0.00752832379945075 | DOWN-regulation |
| CD300E | -7.21004801914166 | 0 | 0.116 | 8.98046632984095E-06 | DOWN-regulation |
| AL445524.1 | -7.16396314977054 | 0 | 0.087 | 0.00210849717285811 | DOWN-regulation |
| SLC15A3 | -7.15529524693508 | 0 | 0.102 | 0.000141902913777861 | DOWN-regulation |
| RBP7 | -7.14646581880049 | 0.003 | 0.195 | 2.52509884710232E-12 | DOWN-regulation |
| CAMK4 | -7.08573055885134 | 0.003 | 0.092 | 0.00232732003089795 | DOWN-regulation |
| LRP1 | -7.03420940953794 | 0 | 0.105 | 0.0000802655583087771 | DOWN-regulation |
| TREM1 | -6.99144708607111 | 0.007 | 0.305 | 2.18239405098614E-23 | DOWN-regulation |
| ALOX5 | -6.98840755647661 | 0.003 | 0.194 | 3.12781701488556E-12 | DOWN-regulation |
| CSF1R | -6.95918052457067 | 0 | 0.099 | 0.000211116915095042 | DOWN-regulation |
| RASSF4 | -6.95435922118217 | 0 | 0.1 | 0.000178094333212891 | DOWN-regulation |
| KYNU | -6.9526777103586 | 0.007 | 0.276 | 2.78470207145426E-20 | DOWN-regulation |

**Table S15.** Top 50 up and down-regulated genes in cluster 7 in CD133+Lin-CD45+ cells.

| **gene** | **avg_log2FC** | **pct.1** | **pct.2** | **p_val_adj** | **expression** |
| --- | --- | --- | --- | --- | --- |
| KLRC1 | 13.2028395655351 | 0.362 | 0 | 4.49245272649033E-276 | UP-regulation |
| XCL1 | 12.1993723571992 | 0.191 | 0 | 1.99781908493108E-144 | UP-regulation |
| SH2D1B | 12.1423914836627 | 0.245 | 0 | 1.88773088988575E-185 | UP-regulation |
| RNF165 | 10.648900996075 | 0.096 | 0 | 8.18753930802119E-71 | UP-regulation |
| KLRF1 | 10.3383844208012 | 0.319 | 0.001 | 3.93004013120066E-220 | UP-regulation |
| FASLG | 10.0198838750068 | 0.064 | 0 | 2.5507987937129E-46 | UP-regulation |
| SPTSSB | 9.88008856436402 | 0.053 | 0 | 3.73448275007887E-38 | UP-regulation |
| LINC02084 | 9.80743063131762 | 0.043 | 0 | 5.52018748142724E-30 | UP-regulation |
| CD160 | 9.7744031711397 | 0.447 | 0.001 | 0 | UP-regulation |
| KRT86 | 9.70119064047303 | 0.053 | 0 | 3.73448275007887E-38 | UP-regulation |
| GRIK4 | 9.66637222300687 | 0.191 | 0.001 | 3.47426698302301E-129 | UP-regulation |
| PRF1 | 9.46298043975615 | 0.553 | 0.002 | 0 | UP-regulation |
| S1PR5 | 9.42652530451947 | 0.149 | 0 | 9.39667918432836E-104 | UP-regulation |
| XCL2 | 9.41136369731317 | 0.383 | 0.001 | 2.85632758370348E-262 | UP-regulation |
| IL2RB | 9.36990013389148 | 0.245 | 0.001 | 7.22748326685336E-170 | UP-regulation |
| LINGO2 | 9.34409606359252 | 0.532 | 0.004 | 0 | UP-regulation |
| AL023803.1 | 9.30930702201743 | 0.011 | 0 | 0.0000236280787612091 | UP-regulation |
| AC008549.2 | 9.2850286058683 | 0.032 | 0 | 8.33332243811134E-22 | UP-regulation |
| GZMH | 9.26298171154256 | 0.319 | 0.001 | 6.52397625425162E-220 | UP-regulation |
| NMUR1 | 9.22911519967985 | 0.032 | 0 | 8.33332243811134E-22 | UP-regulation |
| GNLY | 9.17264669505685 | 0.947 | 0.015 | 0 | UP-regulation |
| KIF19 | 9.08026756551242 | 0.032 | 0 | 8.33332243811134E-22 | UP-regulation |
| LIM2 | 9.07782688068001 | 0.032 | 0 | 8.33332243811134E-22 | UP-regulation |
| AC078980.1 | 9.06615947708157 | 0.021 | 0 | 1.31782240360989E-13 | UP-regulation |
| LGALS9C | 9.01429127873025 | 0.032 | 0 | 8.33332243811134E-22 | UP-regulation |
| TNR | 8.85593815898475 | 0.032 | 0 | 8.33332243811134E-22 | UP-regulation |
| FABP6 | 8.82638904991975 | 0.021 | 0 | 1.31782240360989E-13 | UP-regulation |
| KRT81 | 8.55808689648152 | 0.021 | 0 | 1.31782240360989E-13 | UP-regulation |
| PPP2R2B | 8.47276258576233 | 0.245 | 0.001 | 1.15081716882789E-150 | UP-regulation |
| AL445526.1 | 8.46396121299584 | 0.053 | 0 | 5.08404200256517E-31 | UP-regulation |
| TBX21 | 8.44148848475571 | 0.191 | 0.001 | 1.29629558309126E-122 | UP-regulation |
| AC024028.1 | 8.3533939204201 | 0.064 | 0 | 5.41216180860441E-39 | UP-regulation |
| F11 | 8.28786985135514 | 0.011 | 0 | 0.0000236280787612091 | UP-regulation |
| AC018410.1 | 8.21705744699894 | 0.011 | 0 | 0.0000236280787612091 | UP-regulation |
| AC079834.2 | 8.13073712928676 | 0.011 | 0 | 0.0000236280787612091 | UP-regulation |
| HOGA1 | 8.13073712928676 | 0.011 | 0 | 0.0000236280787612091 | UP-regulation |
| AC012073.1 | 8.12667364927008 | 0.011 | 0 | 0.0000236280787612091 | UP-regulation |
| AC064801.1 | 8.03714238533228 | 0.011 | 0 | 0.0000236280787612091 | UP-regulation |
| IL23R | 7.99292473995396 | 0.032 | 0 | 2.37053295628219E-15 | UP-regulation |
| ANKRD20A4 | 7.96907811038713 | 0.011 | 0 | 0.0000236280787612091 | UP-regulation |
| AC093503.2 | 7.96837672600544 | 0.011 | 0 | 0.0000236280787612091 | UP-regulation |
| LINC01695 | 7.94069301745075 | 0.011 | 0 | 0.0000236280787612091 | UP-regulation |
| FGFBP2 | 7.91977184808545 | 0.426 | 0.005 | 1.42183074195128E-225 | UP-regulation |
| AL359237.1 | 7.9110519186585 | 0.011 | 0 | 0.0000236280787612091 | UP-regulation |
| LINC01019 | 7.8978487516283 | 0.011 | 0 | 0.0000236280787612091 | UP-regulation |
| LGALS9B | 7.86364223099962 | 0.011 | 0 | 0.0000236280787612091 | UP-regulation |
| AL162584.1 | 7.8225554612135 | 0.011 | 0 | 0.0000236280787612091 | UP-regulation |
| TMEM212 | 7.81102315365007 | 0.011 | 0 | 0.0000236280787612091 | UP-regulation |
| LINC02365 | 7.78833859832506 | 0.011 | 0 | 0.0000236280787612091 | UP-regulation |
| LGR6 | 7.73799312458171 | 0.032 | 0 | 2.37053295628219E-15 | UP-regulation |
| S100A12 | -10.5877296590766 | 0 | 0.401 | 9.43633137405144E-10 | DOWN-regulation |
| LYZ | -10.1186056985683 | 0 | 0.433 | 3.27459925745614E-11 | DOWN-regulation |
| LGALS2 | -8.51651759538553 | 0 | 0.309 | 6.65569395312069E-06 | DOWN-regulation |
| AL589693.1 | -8.14445549599681 | 0 | 0.25 | 0.000878990202761439 | DOWN-regulation |
| CLEC7A | -8.01454049503749 | 0 | 0.33 | 1.03578624508101E-06 | DOWN-regulation |
| THBS1 | -7.99041244020674 | 0 | 0.26 | 0.000406760584188019 | DOWN-regulation |
| CFD | -7.94968240600674 | 0 | 0.318 | 2.89600347389974E-06 | DOWN-regulation |
| DOCK4 | -7.65148329631723 | 0 | 0.239 | 0.00204725283967186 | DOWN-regulation |
| ARHGAP24 | -7.53196361056759 | 0 | 0.265 | 0.000275302172121347 | DOWN-regulation |
| SPINK2 | -7.52539718115192 | 0 | 0.306 | 8.33059080583141E-06 | DOWN-regulation |
| HLA-DRA | -7.49694226587661 | 0.011 | 0.667 | 8.85564780007888E-25 | DOWN-regulation |
| KYNU | -7.45886895185969 | 0 | 0.261 | 0.000379777908581842 | DOWN-regulation |
| EMILIN2 | -7.41848819949181 | 0 | 0.288 | 0.0000389372502193745 | DOWN-regulation |
| CSTA | -7.36269890746506 | 0 | 0.269 | 0.000194529643706136 | DOWN-regulation |
| SERPINA1 | -7.34859852786029 | 0 | 0.256 | 0.000559553576829292 | DOWN-regulation |
| ANXA5 | -7.29838442602697 | 0 | 0.327 | 1.34209682559848E-06 | DOWN-regulation |
| MS4A6A | -7.26888119092847 | 0 | 0.255 | 0.000572396124554778 | DOWN-regulation |
| IGSF6 | -7.22181284961151 | 0 | 0.237 | 0.00244102481015629 | DOWN-regulation |
| PID1 | -7.21256205616965 | 0 | 0.206 | 0.0240069991042949 | DOWN-regulation |
| LGALS3 | -7.18206590158495 | 0 | 0.252 | 0.000767984287200399 | DOWN-regulation |
| C1QTNF4 | -7.11773047574407 | 0 | 0.264 | 0.000281723554222183 | DOWN-regulation |
| EREG | -7.10576982574871 | 0 | 0.208 | 0.0211867264470892 | DOWN-regulation |
| DACH1 | -7.06176089441641 | 0 | 0.229 | 0.00459134939268098 | DOWN-regulation |
| GNA15 | -6.95454228222725 | 0 | 0.33 | 9.83323199257997E-07 | DOWN-regulation |
| ERG | -6.92038851494925 | 0 | 0.249 | 0.000983345945525263 | DOWN-regulation |
| AVP | -6.85275805673619 | 0 | 0.204 | 0.0289365786452781 | DOWN-regulation |
| CRHBP | -6.82367474980337 | 0 | 0.244 | 0.00143692346943564 | DOWN-regulation |
| HBEGF | -6.70163536644841 | 0 | 0.221 | 0.00785706154447776 | DOWN-regulation |
| C4orf48 | -6.67634978581141 | 0 | 0.268 | 0.000208566271864985 | DOWN-regulation |
| MSRB3 | -6.63022068974729 | 0 | 0.238 | 0.00223568539874598 | DOWN-regulation |
| LMO2 | -6.57460899211345 | 0 | 0.283 | 0.0000599377553329256 | DOWN-regulation |
| VCAN | -6.5112519960685 | 0.011 | 0.368 | 8.10501014020428E-08 | DOWN-regulation |
| TNS3 | -6.47787441514788 | 0 | 0.258 | 0.000466465233273122 | DOWN-regulation |
| FCN1 | -6.43007955709332 | 0.011 | 0.399 | 3.55297277087082E-09 | DOWN-regulation |
| GNAI1 | -6.37844073474754 | 0 | 0.22 | 0.0087412407896143 | DOWN-regulation |
| BCL11A | -6.36168011527867 | 0 | 0.237 | 0.00233615220104004 | DOWN-regulation |
| SPINT2 | -6.11733163942567 | 0 | 0.226 | 0.00569666905151878 | DOWN-regulation |
| LGALS9 | -6.0575923984288 | 0 | 0.216 | 0.0120175953257325 | DOWN-regulation |
| BASP1 | -5.9588573695001 | 0.011 | 0.437 | 9.5284890955709E-11 | DOWN-regulation |
| S100A9 | -5.8761288439845 | 0.128 | 0.583 | 4.66968194781325E-14 | DOWN-regulation |
| S100A8 | -5.37698280042272 | 0.17 | 0.559 | 8.16396888789989E-11 | DOWN-regulation |
| NCF2 | -5.37042546499969 | 0.011 | 0.326 | 5.59139411790199E-06 | DOWN-regulation |
| HLA-DPA1 | -5.19186490087574 | 0.011 | 0.387 | 2.00787262368573E-08 | DOWN-regulation |
| YBX3 | -5.14010921005564 | 0.011 | 0.491 | 2.83236896797523E-13 | DOWN-regulation |
| LRMDA | -5.12745734330254 | 0.021 | 0.521 | 1.58818672608755E-14 | DOWN-regulation |
| CYBB | -5.03157601635112 | 0.011 | 0.316 | 0.0000140634153323434 | DOWN-regulation |
| CST3 | -5.02818824614748 | 0.053 | 0.51 | 1.56329751532402E-12 | DOWN-regulation |
| DMXL2 | -4.94356430986558 | 0.011 | 0.337 | 2.36451231213538E-06 | DOWN-regulation |
| NKAIN2 | -4.9171037263821 | 0.032 | 0.317 | 0.0000868841465653964 | DOWN-regulation |
| HLA-DRB5 | -4.88842969613824 | 0.011 | 0.411 | 1.97216758439934E-09 | DOWN-regulation |

**Table S16.** Top 50 up and down-regulated genes in cluster 8 in CD133+Lin-CD45+ cells.

| **gene** | **avg_log2FC** | **pct.1** | **pct.2** | **p_val_adj** | **expression** |
| --- | --- | --- | --- | --- | --- |
| MS4A1 | 13.2167167248882 | 0.867 | 0 | 0 | UP-regulation |
| KLHL14 | 12.1832003014806 | 0.313 | 0 | 5.1620327060253E-239 | UP-regulation |
| IGLC7 | 11.9640702512786 | 0.024 | 0 | 7.71382155464217E-16 | UP-regulation |
| FCRLA | 11.8720799448727 | 0.253 | 0 | 2.41908437969374E-192 | UP-regulation |
| AL139020.1 | 11.7254438573767 | 0.217 | 0 | 2.13650462086565E-164 | UP-regulation |
| TNFRSF13B | 11.1947813025119 | 0.169 | 0 | 3.37865310685194E-127 | UP-regulation |
| VPREB3 | 10.066145298106 | 0.566 | 0.001 | 0 | UP-regulation |
| OSBPL10-AS1 | 9.88633027450685 | 0.072 | 0 | 5.67545393080338E-53 | UP-regulation |
| COL19A1 | 9.83967296246591 | 0.602 | 0.001 | 0 | UP-regulation |
| IGLV2-14 | 9.59104278409263 | 0.048 | 0 | 1.9973085544225E-34 | UP-regulation |
| FCRL1 | 9.46717139631981 | 0.747 | 0.001 | 0 | UP-regulation |
| GALNT9 | 9.38604792333596 | 0.048 | 0 | 1.9973085544225E-34 | UP-regulation |
| ZNF860 | 9.29431266361429 | 0.06 | 0 | 1.06031392229197E-43 | UP-regulation |
| AL079338.1 | 9.27505916722522 | 0.036 | 0 | 3.83729910740172E-25 | UP-regulation |
| TCL1A | 9.11215930946812 | 0.771 | 0.002 | 0 | UP-regulation |
| FAM111B | 9.10027201120354 | 0.133 | 0 | 1.53955446056095E-90 | UP-regulation |
| IGKC | 9.09685737298599 | 0.53 | 0.01 | 9.84024463337653E-227 | UP-regulation |
| PAX5 | 9.02541061892271 | 0.747 | 0.001 | 0 | UP-regulation |
| HLA-DOB | 9.0071764546056 | 0.12 | 0 | 2.56611832892097E-81 | UP-regulation |
| IGLV1-51 | 8.98377193643705 | 0.036 | 0 | 3.83729910740172E-25 | UP-regulation |
| LINC02008 | 8.86390969419447 | 0.036 | 0 | 3.83729910740172E-25 | UP-regulation |
| POU2AF1 | 8.84671397725423 | 0.169 | 0.001 | 2.07375119617515E-110 | UP-regulation |
| DAZL | 8.82119280456345 | 0.036 | 0 | 3.83729910740172E-25 | UP-regulation |
| LINC01781 | 8.80064656446514 | 0.205 | 0.001 | 1.30133187111731E-130 | UP-regulation |
| IGLC1 | 8.74416634766576 | 0.361 | 0.001 | 1.82036153324797E-242 | UP-regulation |
| FCRL5 | 8.70328705375419 | 0.181 | 0.001 | 1.76190269895278E-119 | UP-regulation |
| IGKV4-1 | 8.67467730742821 | 0.036 | 0 | 3.83729910740172E-25 | UP-regulation |
| IGLV3-19 | 8.65462674480549 | 0.024 | 0 | 7.71382155464217E-16 | UP-regulation |
| AL161781.2 | 8.64831119988664 | 0.036 | 0 | 3.83729910740172E-25 | UP-regulation |
| TNFRSF13C | 8.59200768016299 | 0.506 | 0.003 | 0 | UP-regulation |
| IGLC2 | 8.52107670584557 | 0.566 | 0.006 | 5.26333438832332E-294 | UP-regulation |
| AC018695.2 | 8.49134166759653 | 0.024 | 0 | 7.71382155464217E-16 | UP-regulation |
| IGHJ6 | 8.45579644970238 | 0.036 | 0 | 3.83729910740172E-25 | UP-regulation |
| FFAR1 | 8.38870814104832 | 0.024 | 0 | 7.71382155464217E-16 | UP-regulation |
| IGHV3-23 | 8.36335556853366 | 0.024 | 0 | 7.71382155464217E-16 | UP-regulation |
| PLD5 | 8.20671565164618 | 0.12 | 0.001 | 7.36682448536685E-68 | UP-regulation |
| GNG3 | 8.15760877906475 | 0.024 | 0 | 7.71382155464217E-16 | UP-regulation |
| CLLU1OS | 8.156467505775 | 0.024 | 0 | 7.71382155464217E-16 | UP-regulation |
| AL449106.1 | 8.13225529056368 | 0.024 | 0 | 7.71382155464217E-16 | UP-regulation |
| AC092747.1 | 8.11794939774432 | 0.012 | 0 | 1.75716120671352E-06 | UP-regulation |
| HTR3A | 8.09445725955645 | 0.024 | 0 | 7.71382155464217E-16 | UP-regulation |
| IGHV3-15 | 8.06435089908986 | 0.024 | 0 | 7.71382155464217E-16 | UP-regulation |
| UGT8 | 8.00975814167934 | 0.06 | 0 | 1.1895138943569E-35 | UP-regulation |
| LINC02397 | 7.99811636792235 | 0.205 | 0 | 6.87566325426727E-146 | UP-regulation |
| LINC02860 | 7.95310382524815 | 0.012 | 0 | 1.75716120671352E-06 | UP-regulation |
| PCDH9-AS2 | 7.93556351456666 | 0.06 | 0 | 1.1895138943569E-35 | UP-regulation |
| AC023090.1 | 7.91668197007264 | 0.012 | 0 | 1.75716120671352E-06 | UP-regulation |
| HRK | 7.84636597154564 | 0.06 | 0.001 | 1.40891194492775E-25 | UP-regulation |
| FCRL2 | 7.79185266758533 | 0.277 | 0 | 1.24153976653003E-201 | UP-regulation |
| C9orf57 | 7.77417510584371 | 0.012 | 0 | 1.75716120671352E-06 | UP-regulation |
| G0S2 | -9.34786070523391 | 0 | 0.375 | 3.07021066207273E-07 | DOWN-regulation |
| CSF3R | -8.37237595420127 | 0 | 0.45 | 2.46235163049292E-10 | DOWN-regulation |
| IL1B | -8.30547136416357 | 0 | 0.338 | 6.90808665797354E-06 | DOWN-regulation |
| AQP9 | -7.96336618601198 | 0 | 0.288 | 0.000349122741030997 | DOWN-regulation |
| FYB1 | -7.92968229828151 | 0 | 0.381 | 1.81230180837966E-07 | DOWN-regulation |
| THBS1 | -7.8063462535711 | 0 | 0.259 | 0.00289406936712278 | DOWN-regulation |
| LCP2 | -7.77591971301854 | 0 | 0.359 | 1.15977865577352E-06 | DOWN-regulation |
| CFD | -7.76561621937109 | 0 | 0.317 | 0.0000367269518241151 | DOWN-regulation |
| C5AR1 | -7.59799301031142 | 0 | 0.297 | 0.000171915791252802 | DOWN-regulation |
| FCAR | -7.55810510644057 | 0 | 0.267 | 0.00160410102900185 | DOWN-regulation |
| DOCK4 | -7.46741710968158 | 0 | 0.238 | 0.0120689827981741 | DOWN-regulation |
| VCAN | -7.38859214402309 | 0.012 | 0.367 | 1.55899185576718E-06 | DOWN-regulation |
| WDFY3 | -7.34299258845495 | 0 | 0.251 | 0.00497425139501937 | DOWN-regulation |
| SPINK2 | -7.34133099451627 | 0 | 0.305 | 0.000093348750049417 | DOWN-regulation |
| CD14 | -7.29193354012866 | 0 | 0.271 | 0.00125333811306911 | DOWN-regulation |
| ATP8B4 | -7.25403226387795 | 0 | 0.281 | 0.000592360199280323 | DOWN-regulation |
| TIMP1 | -7.21681323324795 | 0 | 0.32 | 0.0000286686544292914 | DOWN-regulation |
| CSTA | -7.17863272082941 | 0 | 0.268 | 0.00150835181632096 | DOWN-regulation |
| SERPINA1 | -7.16453234122465 | 0 | 0.255 | 0.0038359916270131 | DOWN-regulation |
| STX11 | -7.03875966942907 | 0 | 0.253 | 0.00450206815997212 | DOWN-regulation |
| IGSF6 | -7.03774666297587 | 0 | 0.236 | 0.0140991945407285 | DOWN-regulation |
| FCN1 | -6.96255632864171 | 0.012 | 0.398 | 1.0110040698217E-07 | DOWN-regulation |
| FGL2 | -6.88966022901071 | 0 | 0.24 | 0.0111636630433409 | DOWN-regulation |
| DACH1 | -6.87769470778076 | 0 | 0.228 | 0.0246437806707971 | DOWN-regulation |
| ITGAX | -6.86019708518508 | 0 | 0.223 | 0.0334202979926188 | DOWN-regulation |
| ETS2 | -6.84864511924254 | 0 | 0.293 | 0.000237582369832968 | DOWN-regulation |
| CALN1 | -6.84111158934834 | 0 | 0.239 | 0.0113835874027926 | DOWN-regulation |
| FCER1G | -6.70004749984545 | 0.012 | 0.47 | 9.21917715260796E-11 | DOWN-regulation |
| CRHBP | -6.63960856316772 | 0 | 0.243 | 0.00882681882933983 | DOWN-regulation |
| CTBP2 | -6.62739101474063 | 0 | 0.281 | 0.000580040449313008 | DOWN-regulation |
| SLC8A1 | -6.5842664776144 | 0.012 | 0.394 | 1.50989396152374E-07 | DOWN-regulation |
| BTBD11 | -6.54770487348075 | 0 | 0.246 | 0.00696917797297215 | DOWN-regulation |
| C19orf38 | -6.52183375237704 | 0 | 0.241 | 0.0101246655679284 | DOWN-regulation |
| PLCB1 | -6.51173177842252 | 0.012 | 0.553 | 8.43550091241584E-15 | DOWN-regulation |
| EGFL7 | -6.46128667319167 | 0 | 0.248 | 0.00631302982758563 | DOWN-regulation |
| RFLNB | -6.37400300376177 | 0 | 0.221 | 0.0388837121838914 | DOWN-regulation |
| TREM1 | -6.35697362638128 | 0.012 | 0.286 | 0.00107816836306676 | DOWN-regulation |
| HOPX | -6.32335434377296 | 0 | 0.234 | 0.016145635401079 | DOWN-regulation |
| LGALS2 | -6.30196071891096 | 0.012 | 0.307 | 0.000218602229404126 | DOWN-regulation |
| GNAI1 | -6.19437454811189 | 0 | 0.219 | 0.043542631183951 | DOWN-regulation |
| EBPL | -6.18082713290936 | 0 | 0.241 | 0.00992847738014143 | DOWN-regulation |
| CXCL8 | -6.12184578497157 | 0.036 | 0.442 | 1.52525898971132E-08 | DOWN-regulation |
| S100A12 | -6.11797339735176 | 0.024 | 0.4 | 4.03950975795376E-07 | DOWN-regulation |
| SRGN | -6.10592269288817 | 0.048 | 0.742 | 3.14470311355384E-25 | DOWN-regulation |
| ZNRF1 | -6.09908807228918 | 0 | 0.231 | 0.0195787177243969 | DOWN-regulation |
| IRAK3 | -6.04473652782957 | 0.012 | 0.434 | 4.27246030296435E-09 | DOWN-regulation |
| FGD4 | -6.02821040582686 | 0.024 | 0.425 | 2.24045604470836E-08 | DOWN-regulation |
| SLCO3A1 | -6.00965970927931 | 0.012 | 0.435 | 3.85080303616248E-09 | DOWN-regulation |
| MNDA | -5.99843373283239 | 0.024 | 0.452 | 1.62806281372163E-09 | DOWN-regulation |
| DOCK5 | -5.98815150469501 | 0.012 | 0.383 | 4.71787917248163E-07 | DOWN-regulation |

**Table S17.** Top 50 up and down-regulated genes in cluster 9 in CD133+Lin-CD45+ cells.

| **gene** | **avg_log2FC** | **pct.1** | **pct.2** | **p_val_adj** | **expression** |
| --- | --- | --- | --- | --- | --- |
| CRISP2 | 12.5712807139358 | 0.087 | 0 | 1.68323070212086E-64 | UP-regulation |
| HS3ST5 | 12.0456333867613 | 0.101 | 0 | 1.08927263950613E-75 | UP-regulation |
| LINC02009 | 11.8623484332481 | 0.058 | 0 | 4.04009839991754E-42 | UP-regulation |
| CEACAM8 | 10.1028369579898 | 0.246 | 0 | 7.09126515623148E-177 | UP-regulation |
| AC005005.3 | 10.0432751891032 | 0.014 | 0 | 1.96990551970065E-08 | UP-regulation |
| LTF | 10.0205698713551 | 0.884 | 0.006 | 0 | UP-regulation |
| PLUT | 10.0166328482637 | 0.014 | 0 | 1.96990551970065E-08 | UP-regulation |
| LBP | 9.92991367925641 | 0.014 | 0 | 1.96990551970065E-08 | UP-regulation |
| AL121974.1 | 9.86964967909009 | 0.014 | 0 | 1.96990551970065E-08 | UP-regulation |
| DEFA3 | 9.6441909075659 | 0.246 | 0.002 | 7.72647894359177E-131 | UP-regulation |
| CTSG | 9.62976785812945 | 0.029 | 0 | 1.05446600966214E-19 | UP-regulation |
| CAMP | 9.61621360663958 | 0.652 | 0.009 | 2.88499242336452E-297 | UP-regulation |
| LYPD8 | 9.52124649758562 | 0.014 | 0 | 1.96990551970065E-08 | UP-regulation |
| VIT | 9.4498519784234 | 0.014 | 0 | 1.96990551970065E-08 | UP-regulation |
| DEFA4 | 9.31547383804328 | 0.116 | 0 | 1.37691933634445E-76 | UP-regulation |
| OR2A7 | 9.24018968098413 | 0.014 | 0 | 1.96990551970065E-08 | UP-regulation |
| PKHD1 | 9.23165398813198 | 0.014 | 0 | 1.96990551970065E-08 | UP-regulation |
| MMP8 | 9.14493345349021 | 0.406 | 0.001 | 4.00765204242716E-263 | UP-regulation |
| OLFM4 | 9.09906412989366 | 0.246 | 0.001 | 3.9469884185663E-167 | UP-regulation |
| DCST1 | 9.06260001975523 | 0.014 | 0 | 1.96990551970065E-08 | UP-regulation |
| SPEF1 | 8.77745260451435 | 0.014 | 0 | 1.96990551970065E-08 | UP-regulation |
| CRISP3 | 8.77524439435275 | 0.507 | 0.002 | 0 | UP-regulation |
| DNASE1L2 | 8.73113803304421 | 0.014 | 0 | 1.96990551970065E-08 | UP-regulation |
| LCN2 | 8.55727991827217 | 0.696 | 0.009 | 0 | UP-regulation |
| MYBPH | 7.98573493873676 | 0.029 | 0 | 6.3267940087563E-12 | UP-regulation |
| AC079921.2 | 7.97341785691836 | 0.014 | 0 | 0.0162874047973304 | UP-regulation |
| COL17A1 | 7.71050208764468 | 0.101 | 0.001 | 2.08985401639304E-51 | UP-regulation |
| NEUROD2 | 7.69326910635672 | 0.014 | 0 | 0.0162874047973304 | UP-regulation |
| TACSTD2 | 7.67491213311099 | 0.014 | 0 | 0.0162874047973304 | UP-regulation |
| HIST1H3G | 7.49404747613342 | 0.029 | 0.001 | 5.10283731579839E-08 | UP-regulation |
| ANKRD18A | 7.46988268221548 | 0.029 | 0.001 | 5.10283731579839E-08 | UP-regulation |
| STOX2 | 7.45233236856471 | 0.116 | 0.003 | 1.45492512827223E-35 | UP-regulation |
| PTGES | 7.43840517347483 | 0.014 | 0 | 0.0162874047973304 | UP-regulation |
| AC009292.1 | 7.43534141057045 | 0.014 | 0 | 1.96990551970065E-08 | UP-regulation |
| CD24 | 7.42314100419871 | 0.42 | 0.008 | 1.91851366059589E-160 | UP-regulation |
| BICDL2 | 7.41236002555694 | 0.014 | 0 | 0.0162874047973304 | UP-regulation |
| CHIT1 | 7.38341059916597 | 0.101 | 0.001 | 5.30804846310569E-42 | UP-regulation |
| AL590999.1 | 7.31425842430466 | 0.014 | 0 | 0.0162874047973304 | UP-regulation |
| OBI1-AS1 | 7.26625925338145 | 0.014 | 0 | 0.0162874047973304 | UP-regulation |
| PCOLCE2 | 7.19937447917069 | 0.029 | 0.001 | 5.10283731579839E-08 | UP-regulation |
| TCN1 | 7.18786044637665 | 0.261 | 0.003 | 4.28074572058754E-125 | UP-regulation |
| BPI | 7.17873070073587 | 0.42 | 0.011 | 1.66686597685976E-135 | UP-regulation |
| CNTF | 7.04606129659958 | 0.029 | 0.001 | 0.0000115358818205932 | UP-regulation |
| AC008622.2 | 6.9900004860223 | 0.014 | 0 | 0.0162874047973304 | UP-regulation |
| CEACAM6 | 6.92879297614553 | 0.072 | 0 | 1.27550568023944E-43 | UP-regulation |
| RETN | 6.92352067780075 | 1 | 0.124 | 7.73486849363579E-118 | UP-regulation |
| INSC | 6.83371062164067 | 0.029 | 0.001 | 5.10283731579839E-08 | UP-regulation |
| ELANE | 6.65062527839464 | 0.043 | 0.001 | 4.29172522600839E-09 | UP-regulation |
| CEBPE | 6.60877843691621 | 0.116 | 0.004 | 1.10076308619255E-29 | UP-regulation |
| AC079298.3 | 6.55037292597062 | 0.014 | 0 | 0.0162874047973304 | UP-regulation |
| CST3 | -9.39075942579848 | 0 | 0.508 | 2.37301238052166E-10 | DOWN-regulation |
| VCAN | -9.25749435873959 | 0 | 0.366 | 0.0000288496040944845 | DOWN-regulation |
| HLA-DRA | -9.2250300042094 | 0 | 0.662 | 1.19285530364465E-17 | DOWN-regulation |
| AREG | -9.12674541106504 | 0 | 0.513 | 1.35293966041707E-10 | DOWN-regulation |
| CD52 | -8.64677406617807 | 0 | 0.695 | 1.79485622870368E-19 | DOWN-regulation |
| S100A10 | -8.6227557986551 | 0 | 0.468 | 8.83761008666689E-09 | DOWN-regulation |
| ZNF331 | -8.47730259956279 | 0 | 0.533 | 2.10183301744685E-11 | DOWN-regulation |
| PLXDC2 | -8.29167769137797 | 0 | 0.476 | 4.08113489064856E-09 | DOWN-regulation |
| LGALS2 | -8.06020819904015 | 0 | 0.307 | 0.00169841360331953 | DOWN-regulation |
| IL1B | -8.03322815445383 | 0 | 0.337 | 0.000229497558804088 | DOWN-regulation |
| CD83 | -7.89133838202508 | 0 | 0.391 | 4.55967207272232E-06 | DOWN-regulation |
| MSI2 | -7.73181388048617 | 0 | 0.402 | 2.00165081467653E-06 | DOWN-regulation |
| LDHB | -7.6372645998978 | 0 | 0.45 | 3.89884653265533E-08 | DOWN-regulation |
| MAML3 | -7.60094257158348 | 0 | 0.462 | 1.44527802674513E-08 | DOWN-regulation |
| CLEC7A | -7.5582310986921 | 0 | 0.327 | 0.000433039260755015 | DOWN-regulation |
| MEF2C | -7.54456508294564 | 0 | 0.491 | 1.14282383379538E-09 | DOWN-regulation |
| PRKCH | -7.54308775139079 | 0 | 0.393 | 3.83929115942368E-06 | DOWN-regulation |
| SLC11A1 | -7.53642676971659 | 0 | 0.321 | 0.0006572911001544 | DOWN-regulation |
| THBS1 | -7.53410304386136 | 0 | 0.258 | 0.0349691453868338 | DOWN-regulation |
| PHACTR1 | -7.53396428784531 | 0 | 0.431 | 2.02164819732804E-07 | DOWN-regulation |
| AFF3 | -7.52793378451288 | 0 | 0.333 | 0.000295100808534768 | DOWN-regulation |
| HLA-DPB1 | -7.39818055078912 | 0 | 0.396 | 3.02726471109439E-06 | DOWN-regulation |
| HDAC9 | -7.38206582977756 | 0 | 0.393 | 4.00821509579668E-06 | DOWN-regulation |
| SOX4 | -7.38128380564263 | 0 | 0.4 | 2.28200692833739E-06 | DOWN-regulation |
| YBX3 | -7.37260560652332 | 0 | 0.487 | 1.51648076762525E-09 | DOWN-regulation |
| INPP4B | -7.35322339148632 | 0 | 0.323 | 0.000575860431711403 | DOWN-regulation |
| CD69 | -7.27037368571581 | 0 | 0.342 | 0.000158437293855227 | DOWN-regulation |
| MAML2 | -7.25250414218271 | 0 | 0.353 | 0.0000717520380876876 | DOWN-regulation |
| BACH2 | -7.19781451954575 | 0 | 0.254 | 0.0449686442214817 | DOWN-regulation |
| BST2 | -7.11398344244112 | 0 | 0.43 | 2.16648892203748E-07 | DOWN-regulation |
| HLA-DRB5 | -7.07184348619566 | 0 | 0.408 | 1.17998243663109E-06 | DOWN-regulation |
| SPINK2 | -7.06908778480653 | 0 | 0.304 | 0.00200300190588552 | DOWN-regulation |
| MALT1 | -7.02274443496588 | 0 | 0.344 | 0.000138092177625717 | DOWN-regulation |
| SULF2 | -7.01576340570558 | 0 | 0.282 | 0.00810741574257425 | DOWN-regulation |
| KYNU | -7.0025595555143 | 0 | 0.259 | 0.0332461341810834 | DOWN-regulation |
| CASC15 | -6.99448164948197 | 0 | 0.282 | 0.00810741630586666 | DOWN-regulation |
| EMILIN2 | -6.96217880314642 | 0 | 0.286 | 0.00622281338000056 | DOWN-regulation |
| ANXA2 | -6.96007760722499 | 0 | 0.283 | 0.00755665321647339 | DOWN-regulation |
| AUTS2 | -6.95872351213178 | 0 | 0.322 | 0.000632947138830896 | DOWN-regulation |
| HLA-DQB1 | -6.9564919726654 | 0 | 0.364 | 0.0000339755231622843 | DOWN-regulation |
| AHR | -6.80730063652047 | 0 | 0.286 | 0.00633393918296339 | DOWN-regulation |
| TLE5 | -6.78021206811699 | 0 | 0.362 | 0.0000391806263773039 | DOWN-regulation |
| FAM49A | -6.74364508452852 | 0 | 0.262 | 0.0276055476538126 | DOWN-regulation |
| USP36 | -6.70259698925246 | 0 | 0.333 | 0.000289465664564547 | DOWN-regulation |
| ALKBH7 | -6.69766851454135 | 0 | 0.39 | 4.96772651062527E-06 | DOWN-regulation |
| DAPK1 | -6.67486457594093 | 0 | 0.303 | 0.00211586055333381 | DOWN-regulation |
| LAPTM4B | -6.67267710627119 | 0 | 0.294 | 0.00384541480257948 | DOWN-regulation |
| SEMA4D | -6.66592455440091 | 0 | 0.273 | 0.0141605595804167 | DOWN-regulation |
| C1QTNF4 | -6.66142107939868 | 0 | 0.262 | 0.0266850126795752 | DOWN-regulation |
| UBE2E2 | -6.63320041450902 | 0 | 0.299 | 0.00277949890864038 | DOWN-regulation |

**Table S18.** Top 50 up and down-regulated genes in cluster 11 in CD133+Lin-CD45+ cells.

| **gene** | **avg_log2FC** | **pct.1** | **pct.2** | **p_val_adj** | **expression** |
| --- | --- | --- | --- | --- | --- |
| DNTT | 11.7668681963358 | 0.164 | 0 | 3.35439287622917E-124 | UP-regulation |
| IRX1 | 10.3034064200299 | 0.082 | 0 | 8.85629604531716E-61 | UP-regulation |
| FAT3 | 9.43697831329184 | 0.23 | 0.001 | 5.30558842178406E-143 | UP-regulation |
| IGHV1-69D | 9.27861119980066 | 0.049 | 0 | 2.07402413339314E-35 | UP-regulation |
| AC073359.2 | 9.22324768324051 | 0.049 | 0 | 2.07402413339314E-35 | UP-regulation |
| AL161912.1 | 9.19543289338969 | 0.049 | 0 | 2.07402413339314E-35 | UP-regulation |
| AC092691.1 | 9.14678957852105 | 0.443 | 0.001 | 0 | UP-regulation |
| LSAMP | 8.99584839149122 | 0.508 | 0.003 | 2.22328447571414E-280 | UP-regulation |
| AL513487.1 | 8.89655180513435 | 0.016 | 0 | 6.05742567500672E-10 | UP-regulation |
| AL162171.3 | 8.8704603401516 | 0.033 | 0 | 1.05428457855661E-22 | UP-regulation |
| NPY | 8.78945912203115 | 0.033 | 0 | 1.05428457855661E-22 | UP-regulation |
| AF064860.1 | 8.55289563588373 | 0.033 | 0 | 1.05428457855661E-22 | UP-regulation |
| ARL5C | 8.50591548799983 | 0.033 | 0 | 1.05428457855661E-22 | UP-regulation |
| ZNF536 | 8.45681750862612 | 0.033 | 0 | 1.05428457855661E-22 | UP-regulation |
| AL096799.1 | 8.43522849228288 | 0.115 | 0 | 1.09993232439078E-74 | UP-regulation |
| AC011586.2 | 8.43043249057895 | 0.016 | 0 | 6.05742567500672E-10 | UP-regulation |
| AC092813.1 | 8.14957149575179 | 0.016 | 0 | 6.05742567500672E-10 | UP-regulation |
| TRBJ2-1 | 8.04144173233188 | 0.016 | 0 | 6.05742567500672E-10 | UP-regulation |
| DMRTA2 | 8.03351542540821 | 0.016 | 0 | 6.05742567500672E-10 | UP-regulation |
| DBNDD1 | 8.03351542540821 | 0.016 | 0 | 6.05742567500672E-10 | UP-regulation |
| KIAA0087 | 7.93531619905272 | 0.098 | 0.001 | 6.59890788009313E-54 | UP-regulation |
| AL357552.2 | 7.91286830475809 | 0.016 | 0 | 6.05742567500672E-10 | UP-regulation |
| AC005339.1 | 7.91286830475809 | 0.016 | 0 | 6.05742567500672E-10 | UP-regulation |
| RAG2 | 7.89125771381891 | 0.049 | 0 | 1.37832456171343E-25 | UP-regulation |
| SLC35F4 | 7.8615875158089 | 0.016 | 0 | 6.05742567500672E-10 | UP-regulation |
| AC026167.1 | 7.80368852984915 | 0.016 | 0 | 6.05742567500672E-10 | UP-regulation |
| LINC02234 | 7.77674163063606 | 0.016 | 0 | 6.05742567500672E-10 | UP-regulation |
| TRPC5OS | 7.77674163063606 | 0.016 | 0 | 6.05742567500672E-10 | UP-regulation |
| LINC02129 | 7.76823954012659 | 0.016 | 0 | 6.05742567500672E-10 | UP-regulation |
| CCDC148 | 7.7595343352293 | 0.016 | 0 | 6.05742567500672E-10 | UP-regulation |
| AC098484.2 | 7.7526350645072 | 0.016 | 0 | 6.05742567500672E-10 | UP-regulation |
| AC069542.1 | 7.74579261893848 | 0.016 | 0 | 6.05742567500672E-10 | UP-regulation |
| AC104984.5 | 7.70507789302758 | 0.016 | 0 | 6.05742567500672E-10 | UP-regulation |
| AC068196.1 | 7.70318867570715 | 0.016 | 0 | 6.05742567500672E-10 | UP-regulation |
| AC005381.1 | 7.69915506069933 | 0.016 | 0 | 6.05742567500672E-10 | UP-regulation |
| LRRC72 | 7.65963290151138 | 0.016 | 0 | 6.05742567500672E-10 | UP-regulation |
| LINC01797 | 7.65124875271539 | 0.016 | 0 | 6.05742567500672E-10 | UP-regulation |
| AC007823.1 | 7.62027041355204 | 0.016 | 0 | 6.05742567500672E-10 | UP-regulation |
| AL645608.1 | 7.59492485589586 | 0.016 | 0 | 6.05742567500672E-10 | UP-regulation |
| AC093298.2 | 7.56154356856355 | 0.016 | 0 | 6.05742567500672E-10 | UP-regulation |
| SOAT2 | 7.52977500705802 | 0.016 | 0 | 6.05742567500672E-10 | UP-regulation |
| RRS1-AS1 | 7.50554122358999 | 0.016 | 0 | 6.05742567500672E-10 | UP-regulation |
| CYGB | 7.49498059729351 | 0.262 | 0.001 | 7.26971299315594E-159 | UP-regulation |
| CERNA2 | 7.47521431853038 | 0.016 | 0 | 6.05742567500672E-10 | UP-regulation |
| PCAT14 | 7.47521431853038 | 0.016 | 0 | 6.05742567500672E-10 | UP-regulation |
| AC112487.1 | 7.44860416932176 | 0.016 | 0 | 6.05742567500672E-10 | UP-regulation |
| MUC4 | 7.44860416932176 | 0.016 | 0 | 6.05742567500672E-10 | UP-regulation |
| GUCY2F | 7.43044801030141 | 0.016 | 0 | 6.05742567500672E-10 | UP-regulation |
| AL512306.2 | 7.41791414545554 | 0.016 | 0 | 6.05742567500672E-10 | UP-regulation |
| LINC01949 | 7.41791414545554 | 0.016 | 0 | 6.05742567500672E-10 | UP-regulation |
| CXCL8 | -9.74345273393319 | 0 | 0.44 | 0.0000015318603501002 | DOWN-regulation |
| LYZ | -9.48124608850738 | 0 | 0.429 | 3.56486538002942E-06 | DOWN-regulation |
| G0S2 | -8.89456728180862 | 0 | 0.372 | 0.000171066363526791 | DOWN-regulation |
| FCN1 | -8.53963396521537 | 0 | 0.395 | 0.0000367304280662419 | DOWN-regulation |
| VCAN | -7.505109157936 | 0.016 | 0.365 | 0.000717453748074624 | DOWN-regulation |
| TREM1 | -7.50368994325154 | 0 | 0.285 | 0.0329491745278327 | DOWN-regulation |
| DMXL2 | -7.37521173093512 | 0 | 0.334 | 0.00184331913306796 | DOWN-regulation |
| CYBB | -7.14175440350188 | 0 | 0.313 | 0.00639553521248839 | DOWN-regulation |
| TYMP | -7.08130905236281 | 0 | 0.325 | 0.00322905024995405 | DOWN-regulation |
| ANXA2 | -6.77902739350945 | 0 | 0.282 | 0.0373371501937169 | DOWN-regulation |
| S100A10 | -6.59753556020683 | 0.016 | 0.466 | 6.09870040906858E-07 | DOWN-regulation |
| MNDA | -6.3545285592765 | 0.016 | 0.45 | 2.20945028947016E-06 | DOWN-regulation |
| SLC11A1 | -5.78383307197908 | 0.016 | 0.32 | 0.0117813238553949 | DOWN-regulation |
| S100A9 | -5.77572508622929 | 0.23 | 0.577 | 5.09527056367567E-06 | DOWN-regulation |
| LGALS2 | -5.61460203309445 | 0.016 | 0.306 | 0.0295990803854403 | DOWN-regulation |
| S100A6 | -5.57564324987652 | 0.164 | 0.672 | 1.62066907931119E-11 | DOWN-regulation |
| S100A8 | -5.55692351600711 | 0.082 | 0.557 | 3.02101334045895E-08 | DOWN-regulation |
| CLEC7A | -5.49018872379092 | 0.016 | 0.326 | 0.00866982531949314 | DOWN-regulation |
| CFD | -5.4719680415257 | 0.016 | 0.315 | 0.0171053800271676 | DOWN-regulation |
| IL1B | -5.46400006803896 | 0.033 | 0.335 | 0.0140741917819268 | DOWN-regulation |
| S100A12 | -5.45660017196962 | 0.033 | 0.397 | 0.000430358158489069 | DOWN-regulation |
| SAMHD1 | -5.40246980387342 | 0.016 | 0.329 | 0.00678760680419079 | DOWN-regulation |
| IFI30 | -5.33346548964332 | 0.033 | 0.38 | 0.000738294827303403 | DOWN-regulation |
| FGD4 | -4.9459041526347 | 0.049 | 0.422 | 0.000123428242767797 | DOWN-regulation |
| CST3 | -4.82361318905402 | 0.131 | 0.504 | 0.0000326879700705716 | DOWN-regulation |
| PLAUR | -4.79871156806699 | 0.082 | 0.424 | 0.000641251074395944 | DOWN-regulation |
| SIPA1L1 | -4.67349052062425 | 0.098 | 0.548 | 9.39216762579692E-08 | DOWN-regulation |
| FCER1G | -4.59623187638868 | 0.066 | 0.467 | 0.0000137646577028619 | DOWN-regulation |
| PLEK | -4.54291002094153 | 0.049 | 0.39 | 0.0014288038117591 | DOWN-regulation |
| S100A11 | -4.50824083708209 | 0.148 | 0.58 | 1.0025413806169E-07 | DOWN-regulation |
| NAMPT | -4.37236211288639 | 0.295 | 0.63 | 2.01475453839024E-06 | DOWN-regulation |
| TYROBP | -4.22868170851737 | 0.18 | 0.517 | 0.0000480124814829011 | DOWN-regulation |
| RBM47 | -4.20858926191595 | 0.066 | 0.374 | 0.010179906895205 | DOWN-regulation |
| NFIL3 | -4.17944161127825 | 0.049 | 0.336 | 0.038972676762714 | DOWN-regulation |
| COTL1 | -4.14590196965106 | 0.049 | 0.438 | 0.0000731748635255557 | DOWN-regulation |
| SRGN | -4.06493292990056 | 0.23 | 0.735 | 1.60796064637667E-12 | DOWN-regulation |
| CD83 | -4.03814688257645 | 0.066 | 0.389 | 0.00431640145853532 | DOWN-regulation |
| NCF1 | -4.01760722028189 | 0.049 | 0.357 | 0.0144514218520181 | DOWN-regulation |
| CRIP1 | -3.99413271764593 | 0.148 | 0.476 | 0.000613298691325546 | DOWN-regulation |
| KLF2 | -3.85041753947341 | 0.049 | 0.338 | 0.0456636548708603 | DOWN-regulation |
| ANXA5 | -3.78011795461515 | 0.033 | 0.323 | 0.0448017065595458 | DOWN-regulation |
| PSAP | -3.70014426178984 | 0.115 | 0.472 | 0.000225701129760553 | DOWN-regulation |
| LYST | -3.64874391405591 | 0.049 | 0.356 | 0.0170135971590506 | DOWN-regulation |
| ATP2B1-AS1 | -3.63377020057163 | 0.082 | 0.371 | 0.0411525228301509 | DOWN-regulation |
| FCGRT | -3.61146422470634 | 0.066 | 0.38 | 0.00759166846618911 | DOWN-regulation |
| SAT1 | -3.5494793053534 | 0.377 | 0.626 | 0.000474131577172592 | DOWN-regulation |
| CSF3R | -3.4880168433102 | 0.148 | 0.445 | 0.0131642380153515 | DOWN-regulation |
| RAB31 | -3.4543748031535 | 0.066 | 0.361 | 0.0352139428857803 | DOWN-regulation |
| FYB1 | -3.3060480503348 | 0.082 | 0.377 | 0.0358298907172227 | DOWN-regulation |
| FNDC3B | -3.10206311922352 | 0.164 | 0.461 | 0.0121005083860838 | DOWN-regulation |

**Table S19.** Top 50 up-and down-regulated genes in clusters expressing CD34 and PROM1 genes (clusters 0 and 1) compared to clusters expressing only the CD34 gene (clusters 6 and 7) in CD34+Lin-CD45+ cells.

| **gene** | **avg_log2FC** | **pct.1** | **pct.2** | **p_val_adj** | **expression** |
| --- | --- | --- | --- | --- | --- |
| PDE10A | 5.01842406675047 | 0.059 | 0.002 | 0.000489210237400501 | UP-regulation |
| AL713852.1 | 4.96815435835152 | 0.062 | 0.002 | 0.000187547153436052 | UP-regulation |
| AC105114.2 | 4.78652210420508 | 0.053 | 0.002 | 0.00346626184035569 | UP-regulation |
| HFM1 | 4.18214981839155 | 0.058 | 0.004 | 0.00186124371839068 | UP-regulation |
| TC2N | 4.09806755122011 | 0.067 | 0.004 | 0.000130405102936315 | UP-regulation |
| AP002989.1 | 3.64191211255581 | 0.049 | 0.004 | 0.0283766453252669 | UP-regulation |
| LIMCH1 | 3.63823109480509 | 0.153 | 0.016 | 3.05273162573813E-13 | UP-regulation |
| MAGI1 | 3.62838536184163 | 0.073 | 0.007 | 0.000197701443514417 | UP-regulation |
| THRB | 3.46534014171901 | 0.217 | 0.022 | 1.98173559893874E-21 | UP-regulation |
| ARHGAP20 | 3.46270583607359 | 0.102 | 0.009 | 8.00055239181711E-08 | UP-regulation |
| NLGN1 | 3.18206383147117 | 0.224 | 0.036 | 4.31013195009584E-19 | UP-regulation |
| LINC01725 | 3.01917221534328 | 0.077 | 0.007 | 0.000073833981283904 | UP-regulation |
| WWC2 | 3.00217141314508 | 0.066 | 0.007 | 0.00181030853619747 | UP-regulation |
| MICAL2 | 2.94889249442015 | 0.072 | 0.009 | 0.000912782183371969 | UP-regulation |
| PREX2 | 2.94299528291125 | 0.327 | 0.036 | 2.76025027430739E-35 | UP-regulation |
| AC092839.1 | 2.90209969664505 | 0.094 | 0.013 | 8.32522945989341E-06 | UP-regulation |
| AC004160.1 | 2.88451546950692 | 0.07 | 0.007 | 0.000473098671739362 | UP-regulation |
| AF127577.2 | 2.87268346224877 | 0.254 | 0.044 | 5.24809863980442E-22 | UP-regulation |
| TJP2 | 2.86433263803457 | 0.072 | 0.011 | 0.00243612535773604 | UP-regulation |
| TPST1 | 2.86286068536386 | 0.178 | 0.029 | 1.14100784157686E-13 | UP-regulation |
| ACOT11 | 2.81474083727556 | 0.08 | 0.015 | 0.00127475817408165 | UP-regulation |
| ESRRG | 2.78005852128638 | 0.063 | 0.011 | 0.0322644327800343 | UP-regulation |
| SOX5 | 2.77983920541771 | 0.094 | 0.015 | 0.0000232290803778932 | UP-regulation |
| NFIA | 2.76237519493539 | 0.35 | 0.068 | 8.51368707284654E-33 | UP-regulation |
| SORBS1 | 2.75557966210224 | 0.062 | 0.011 | 0.0399747551138939 | UP-regulation |
| CDC14B | 2.7317754656217 | 0.135 | 0.018 | 5.55234909967458E-10 | UP-regulation |
| SLC22A15 | 2.72211839251169 | 0.094 | 0.013 | 7.24972385671931E-06 | UP-regulation |
| MEG8 | 2.71713026231263 | 0.102 | 0.015 | 1.81030219758237E-06 | UP-regulation |
| MECOM | 2.71156061628042 | 0.304 | 0.053 | 9.4925861192612E-28 | UP-regulation |
| MYO1D | 2.70983391809477 | 0.071 | 0.013 | 0.00812280167459996 | UP-regulation |
| SPIRE1 | 2.70671467153248 | 0.198 | 0.033 | 1.24903215629232E-15 | UP-regulation |
| RNLS | 2.69745679225168 | 0.081 | 0.013 | 0.000358272366162518 | UP-regulation |
| LINC00923 | 2.68395566442083 | 0.06 | 0.009 | 0.0263173621881065 | UP-regulation |
| DISC1FP1 | 2.68093824750218 | 0.101 | 0.022 | 0.000092675021692299 | UP-regulation |
| ZNF804A | 2.66359877463448 | 0.133 | 0.026 | 4.29660785866382E-08 | UP-regulation |
| AC002429.2 | 2.65857314619401 | 0.065 | 0.013 | 0.0455952811445867 | UP-regulation |
| ARHGAP21 | 2.64923466891805 | 0.183 | 0.035 | 4.92467962253281E-13 | UP-regulation |
| UGGT2 | 2.63240347979694 | 0.179 | 0.038 | 5.71201802291442E-12 | UP-regulation |
| ARHGEF11 | 2.61270169892174 | 0.067 | 0.009 | 0.00353304438748662 | UP-regulation |
| PDZD2 | 2.58249851910731 | 0.416 | 0.097 | 8.04777393743613E-39 | UP-regulation |
| MEG3 | 2.57155569083378 | 0.224 | 0.042 | 3.3654632639917E-17 | UP-regulation |
| SYT1 | 2.5702796764074 | 0.285 | 0.066 | 4.20071858962752E-22 | UP-regulation |
| PPM1H | 2.55450943036226 | 0.292 | 0.051 | 1.04327551259468E-25 | UP-regulation |
| EPGN | 2.54983095248392 | 0.244 | 0.04 | 9.75790300309642E-21 | UP-regulation |
| AUXG01000058.1 | 2.53462447686073 | 0.09 | 0.015 | 0.0000710767899542504 | UP-regulation |
| LRP6 | 2.52656579918975 | 0.141 | 0.022 | 6.32837156184543E-10 | UP-regulation |
| FGD5 | 2.48816475416311 | 0.126 | 0.026 | 3.02779712900345E-07 | UP-regulation |
| ZFPM2 | 2.48670553909573 | 0.082 | 0.013 | 0.000258754862761064 | UP-regulation |
| CCDC175 | 2.45634414457821 | 0.083 | 0.02 | 0.00661740287348569 | UP-regulation |
| KIAA1211 | 2.41730231307252 | 0.384 | 0.091 | 7.54050139997487E-33 | UP-regulation |
| PTGDS | -10.6285001750297 | 0 | 0.024 | 0.0000201572590207327 | DOWN-regulation |
| DNTT | -9.42317837927541 | 0 | 0.068 | 5.42231862412626E-21 | DOWN-regulation |
| LINC01374 | -9.20195391366508 | 0.001 | 0.193 | 2.57391713227079E-66 | DOWN-regulation |
| ARPP21 | -8.41712388109753 | 0 | 0.031 | 5.17972278787591E-08 | DOWN-regulation |
| IRX1 | -8.21148759125377 | 0 | 0.047 | 7.72058203971205E-14 | DOWN-regulation |
| GZMB | -7.8901066016896 | 0.001 | 0.036 | 4.99194070122893E-08 | DOWN-regulation |
| IGLC1 | -7.78755114062626 | 0 | 0.015 | 0.0361533840076739 | DOWN-regulation |
| AL139340.1 | -7.46547331487456 | 0 | 0.029 | 2.29849472124367E-07 | DOWN-regulation |
| EBF1 | -7.29172902132123 | 0.004 | 0.142 | 1.90911785996976E-42 | DOWN-regulation |
| LSAMP | -7.27696707034238 | 0.004 | 0.113 | 7.62854864399975E-32 | DOWN-regulation |
| PACSIN1 | -7.20420008759426 | 0.001 | 0.062 | 5.20396326483515E-18 | DOWN-regulation |
| NRP1 | -7.19470166957342 | 0 | 0.015 | 0.0361533840076739 | DOWN-regulation |
| IRF8 | -7.16326429223239 | 0.002 | 0.142 | 3.50127647451363E-45 | DOWN-regulation |
| IGHV1-69D | -7.15996598739272 | 0 | 0.022 | 0.0000897533571999465 | DOWN-regulation |
| IGLC2 | -7.08869188464321 | 0.001 | 0.031 | 5.33359500450492E-07 | DOWN-regulation |
| JCHAIN | -7.02480579430787 | 0.004 | 0.181 | 1.06251209920268E-56 | DOWN-regulation |
| VPREB3 | -6.89609286825032 | 0 | 0.015 | 0.0361533840076739 | DOWN-regulation |
| CYGB | -6.88864287592781 | 0.002 | 0.086 | 1.29685475602713E-24 | DOWN-regulation |
| AC092691.1 | -6.8289301551471 | 0.002 | 0.084 | 6.15632252339219E-24 | DOWN-regulation |
| AL096799.1 | -6.79786690808286 | 0 | 0.015 | 0.0361533840076739 | DOWN-regulation |
| IRF4 | -6.4563478831282 | 0.002 | 0.027 | 0.00046499564516432 | DOWN-regulation |
| BLNK | -6.37979370254998 | 0.001 | 0.038 | 1.39375647291834E-09 | DOWN-regulation |
| TRBVB | -6.31065378876794 | 0 | 0.015 | 0.0361533840076739 | DOWN-regulation |
| TRBV28 | -6.0812259594534 | 0 | 0.015 | 0.0361533840076739 | DOWN-regulation |
| AL354949.1 | -6.08003992231669 | 0.001 | 0.027 | 0.0000102644995704578 | DOWN-regulation |
| FAT3 | -5.84676172488312 | 0.002 | 0.049 | 1.24085118349777E-11 | DOWN-regulation |
| TRIB2 | -5.73024496426933 | 0.001 | 0.022 | 0.000895992172352368 | DOWN-regulation |
| PTCRA | -5.49890286401628 | 0.001 | 0.027 | 0.0000788380524077467 | DOWN-regulation |
| BLK | -5.49141341751016 | 0.002 | 0.044 | 1.03002092328141E-09 | DOWN-regulation |
| NIBAN3 | -5.45515995789251 | 0.006 | 0.117 | 1.21080936966464E-30 | DOWN-regulation |
| CLIC3 | -5.45254379458307 | 0.001 | 0.026 | 0.000341306283320046 | DOWN-regulation |
| LINC01226 | -5.43933866941055 | 0.001 | 0.024 | 0.000202915325413968 | DOWN-regulation |
| SPIB | -5.34382480186453 | 0.001 | 0.027 | 0.0000801017404493098 | DOWN-regulation |
| IFNG-AS1 | -5.32194537425408 | 0.008 | 0.093 | 3.48349752682017E-20 | DOWN-regulation |
| NPTX2 | -5.31825744558859 | 0.001 | 0.046 | 3.23524138837355E-11 | DOWN-regulation |
| LY86-AS1 | -5.16378956648899 | 0.004 | 0.055 | 1.70975120651364E-10 | DOWN-regulation |
| MS4A1 | -5.15216042734742 | 0.001 | 0.024 | 0.000204305523778633 | DOWN-regulation |
| AP000345.2 | -4.95350608460578 | 0.001 | 0.02 | 0.0270083165207336 | DOWN-regulation |
| MME | -4.93438257370431 | 0.015 | 0.179 | 4.0773325528457E-42 | DOWN-regulation |
| PDGFRB | -4.91213169501389 | 0.001 | 0.027 | 0.0000785876541694474 | DOWN-regulation |
| CD79A | -4.80666940663925 | 0.011 | 0.133 | 1.38164791150399E-30 | DOWN-regulation |
| IGKC | -4.76523055108526 | 0.003 | 0.04 | 1.22221561645051E-07 | DOWN-regulation |
| CHST15 | -4.75215363497244 | 0.001 | 0.024 | 0.00144099614039546 | DOWN-regulation |
| AC019197.1 | -4.6240788140719 | 0.004 | 0.077 | 6.49481882438686E-18 | DOWN-regulation |
| MME-AS1 | -4.59149193814722 | 0.001 | 0.018 | 0.0175390042693046 | DOWN-regulation |
| LINC00996 | -4.56214209557577 | 0.003 | 0.026 | 0.0363177355382081 | DOWN-regulation |
| FGD2 | -4.47854919549437 | 0.002 | 0.024 | 0.00810245027103519 | DOWN-regulation |
| RBMS3 | -4.45583616879417 | 0.005 | 0.04 | 0.0000401061351861882 | DOWN-regulation |
| SCN3A | -4.39730925766738 | 0.018 | 0.177 | 1.91947655921411E-38 | DOWN-regulation |
| TRPM2 | -4.38492750915717 | 0.003 | 0.057 | 1.66476246212652E-12 | DOWN-regulation |

**Table S20.** Top 50 up-and down-regulated genes in clusters expressing CD34 and PROM1 genes (clusters 0 and 1) in comparison to clusters expressing cell differentiation markers (CD2, CD4, CD14) (clusters 2, 3, and 4) in CD34+Lin-CD45+ cells.

| **gene** | **avg_log2FC** | **pct.1** | **pct.2** | **p_val_adj** | **expression** |
| --- | --- | --- | --- | --- | --- |
| SCN3A | 9.10725946091621 | 0.059 | 0 | 3.70957791493668E-17 | UP-regulation |
| CFH | 9.02261145709126 | 0.106 | 0 | 8.89459828186365E-34 | UP-regulation |
| MYCT1 | 8.99568245837239 | 0.112 | 0 | 7.51886657267633E-36 | UP-regulation |
| MIR100HG | 8.93970558314907 | 0.081 | 0 | 6.06815406043536E-25 | UP-regulation |
| AC107223.1 | 8.897910709133 | 0.096 | 0 | 3.51124724093333E-30 | UP-regulation |
| PXDN | 8.85831152821346 | 0.099 | 0 | 2.24429491173281E-31 | UP-regulation |
| MPDZ | 8.85356405708505 | 0.096 | 0 | 5.19674217866683E-30 | UP-regulation |
| AL355612.1 | 8.73195991191402 | 0.082 | 0 | 4.12477594981792E-25 | UP-regulation |
| VPREB1 | 8.72905493157367 | 0.052 | 0 | 1.51424712573188E-14 | UP-regulation |
| AC016735.1 | 8.58690907901079 | 0.077 | 0 | 2.85068833964611E-23 | UP-regulation |
| IFNG-AS1 | 8.50714134684568 | 0.03 | 0 | 5.63544556829139E-07 | UP-regulation |
| CTSF | 8.47574942600681 | 0.075 | 0 | 9.01312422163239E-23 | UP-regulation |
| DPY19L2 | 8.47272335133954 | 0.072 | 0 | 1.92464534307665E-21 | UP-regulation |
| CD34 | 8.44962694895409 | 0.332 | 0.001 | 2.87886759266469E-127 | UP-regulation |
| CD79A | 8.41253993635555 | 0.042 | 0 | 2.61884176390817E-11 | UP-regulation |
| CCDC102A | 8.41192117497573 | 0.075 | 0 | 9.01312581902621E-23 | UP-regulation |
| AC098617.1 | 8.38464182215806 | 0.061 | 0 | 1.19572279453331E-17 | UP-regulation |
| SOCS2-AS1 | 8.34995455497391 | 0.069 | 0 | 1.29597678671611E-20 | UP-regulation |
| MAGI1 | 8.27311149602991 | 0.056 | 0 | 5.17528139223447E-16 | UP-regulation |
| COL6A2 | 8.22623541965634 | 0.064 | 0 | 1.23635178918059E-18 | UP-regulation |
| KIF7 | 8.16263557144172 | 0.065 | 0 | 2.71377723730616E-19 | UP-regulation |
| AC002429.2 | 8.13630907187911 | 0.051 | 0 | 2.2016593977071E-14 | UP-regulation |
| OBSL1 | 8.07302284370295 | 0.06 | 0 | 2.54392092161124E-17 | UP-regulation |
| DSG2 | 8.07038451263411 | 0.063 | 0 | 2.63603715371201E-18 | UP-regulation |
| PDE1A | 8.05047756137599 | 0.161 | 0.001 | 5.92428561854604E-54 | UP-regulation |
| DPPA4 | 8.03338600295047 | 0.166 | 0.001 | 5.41635089011818E-56 | UP-regulation |
| VWDE | 7.98867022258207 | 0.054 | 0 | 3.38266702636903E-15 | UP-regulation |
| SMIM24 | 7.97274061576009 | 0.365 | 0.002 | 1.19097671041719E-142 | UP-regulation |
| MAPK12 | 7.96869870707629 | 0.056 | 0 | 7.53611820909998E-16 | UP-regulation |
| TEX9 | 7.95443314125409 | 0.053 | 0 | 4.9215616054768E-15 | UP-regulation |
| IGSF10 | 7.94776321102748 | 0.052 | 0 | 1.51424712573188E-14 | UP-regulation |
| PRKG2 | 7.94350458615859 | 0.049 | 0 | 9.82314660561811E-14 | UP-regulation |
| BEND5 | 7.93266328531458 | 0.054 | 0 | 2.32456734726569E-15 | UP-regulation |
| AEBP1 | 7.86910738337623 | 0.052 | 0 | 1.04128902751515E-14 | UP-regulation |
| PLSCR4 | 7.85823346108113 | 0.052 | 0 | 1.51424703841574E-14 | UP-regulation |
| CYGB | 7.78278216623122 | 0.024 | 0 | 0.0000670457637291989 | UP-regulation |
| COL4A5 | 7.76718952321373 | 0.041 | 0 | 5.50109051467273E-11 | UP-regulation |
| SEMA6A | 7.7551050052079 | 0.119 | 0.001 | 7.17539711844629E-38 | UP-regulation |
| INSYN2A | 7.72244885533988 | 0.045 | 0 | 2.81555213767185E-12 | UP-regulation |
| PARD6B | 7.68454062454466 | 0.049 | 0 | 9.82314434490566E-14 | UP-regulation |
| MFAP4 | 7.68450134766556 | 0.044 | 0 | 8.59264786379983E-12 | UP-regulation |
| HOOK1 | 7.62876078940042 | 0.047 | 0 | 6.34674104167122E-13 | UP-regulation |
| LINC02839 | 7.62414626686698 | 0.038 | 0 | 7.35750471588902E-10 | UP-regulation |
| XXYLT1-AS2 | 7.62274879674244 | 0.046 | 0 | 1.94048746076023E-12 | UP-regulation |
| CCDC141 | 7.6140241713829 | 0.042 | 0 | 3.79585932686871E-11 | UP-regulation |
| HOXA6 | 7.60921110096225 | 0.047 | 0 | 9.21308028895989E-13 | UP-regulation |
| DYTN | 7.58008879581895 | 0.11 | 0.001 | 1.40118889676469E-34 | UP-regulation |
| EHD2 | 7.55949361423577 | 0.047 | 0 | 6.34674067729717E-13 | UP-regulation |
| LINC01122 | 7.55741784154257 | 0.141 | 0.001 | 2.51761678993993E-46 | UP-regulation |
| ZNF667 | 7.55571299242805 | 0.115 | 0.001 | 1.14938929457269E-36 | UP-regulation |
| CX3CR1 | -10.7536099343944 | 0 | 0.169 | 2.15531852936632E-81 | DOWN-regulation |
| FCGR3A | -10.6072986095462 | 0 | 0.079 | 2.79281336157487E-35 | DOWN-regulation |
| NOD2 | -10.1715778849301 | 0 | 0.114 | 1.40711565691347E-52 | DOWN-regulation |
| CD300E | -10.0444511428848 | 0 | 0.258 | 6.74484139323884E-130 | DOWN-regulation |
| SERPINB2 | -9.9395889401432 | 0 | 0.07 | 1.30554368387506E-30 | DOWN-regulation |
| TLR8 | -9.75792487484846 | 0 | 0.085 | 5.7915606127947E-38 | DOWN-regulation |
| CLEC4D | -9.50814762987082 | 0 | 0.059 | 1.21240353613704E-25 | DOWN-regulation |
| LAMB3 | -9.44533765864337 | 0 | 0.072 | 1.31180016565291E-31 | DOWN-regulation |
| HK3 | -9.43969472881143 | 0 | 0.066 | 1.28009584872859E-28 | DOWN-regulation |
| MARCO | -9.39520948291431 | 0 | 0.066 | 1.28009569827441E-28 | DOWN-regulation |
| AC037198.1 | -9.36938840388205 | 0 | 0.066 | 5.96674743515316E-29 | DOWN-regulation |
| MEFV | -9.22910209557495 | 0 | 0.171 | 9.15086059544098E-82 | DOWN-regulation |
| HPSE | -9.18958796266486 | 0 | 0.051 | 1.07852249329631E-21 | DOWN-regulation |
| MTMR11 | -9.06250311726388 | 0 | 0.055 | 1.15062782076794E-23 | DOWN-regulation |
| CD300C | -8.9272532976979 | 0 | 0.051 | 1.07852249329631E-21 | DOWN-regulation |
| WLS | -8.73288935833082 | 0 | 0.041 | 8.718333402501E-17 | DOWN-regulation |
| LINC01010 | -8.70106807046677 | 0 | 0.042 | 4.11322565577644E-17 | DOWN-regulation |
| LGALS2 | -8.66072923760298 | 0.006 | 0.699 | 0 | DOWN-regulation |
| C5AR2 | -8.61158441940467 | 0 | 0.041 | 1.84740179780859E-16 | DOWN-regulation |
| CLEC10A | -8.59162641288041 | 0 | 0.032 | 3.13362763094804E-12 | DOWN-regulation |
| CSTA | -8.49956450611334 | 0.003 | 0.454 | 9.89149331171592E-248 | DOWN-regulation |
| CATSPER1 | -8.48853533353138 | 0 | 0.036 | 3.51708948183403E-14 | DOWN-regulation |
| CYP1B1 | -8.46750573922168 | 0 | 0.09 | 6.14043827282844E-40 | DOWN-regulation |
| VCAN | -8.29686766043468 | 0.016 | 0.868 | 0 | DOWN-regulation |
| SIGLEC7 | -8.27917664150654 | 0 | 0.034 | 1.57225832945857E-13 | DOWN-regulation |
| LONRF3 | -8.23126020630859 | 0.002 | 0.258 | 1.93802701045208E-127 | DOWN-regulation |
| FCGR2B | -8.22123903402894 | 0 | 0.03 | 2.94978413529527E-11 | DOWN-regulation |
| PID1 | -8.21408789254298 | 0.003 | 0.486 | 1.58696093315028E-269 | DOWN-regulation |
| SDC2 | -8.21034225487123 | 0 | 0.09 | 6.25267387149912E-40 | DOWN-regulation |
| ASGR2 | -8.17855819379938 | 0 | 0.093 | 1.27897427441374E-41 | DOWN-regulation |
| VCAN-AS1 | -8.04376782451702 | 0 | 0.078 | 3.21218388689781E-34 | DOWN-regulation |
| MAFB | -8.02282343455663 | 0.002 | 0.232 | 2.70978212613509E-112 | DOWN-regulation |
| RBP7 | -8.01713526912777 | 0.002 | 0.29 | 4.22994121374519E-146 | DOWN-regulation |
| CD300LB | -7.9784367868686 | 0 | 0.071 | 1.49807456315384E-30 | DOWN-regulation |
| ARHGEF10L | -7.92678714260025 | 0 | 0.106 | 4.66755913637428E-48 | DOWN-regulation |
| OSCAR | -7.86977367127808 | 0.001 | 0.111 | 8.79739560959261E-50 | DOWN-regulation |
| LIPN | -7.86731281778101 | 0.001 | 0.129 | 4.33729718584725E-59 | DOWN-regulation |
| LGALS3 | -7.86546041907014 | 0.003 | 0.46 | 2.83053421529019E-251 | DOWN-regulation |
| SERPINA1 | -7.85773561646147 | 0.004 | 0.465 | 4.62814670322789E-254 | DOWN-regulation |
| CD14 | -7.84899842398885 | 0.005 | 0.498 | 1.76276569924505E-274 | DOWN-regulation |
| C3AR1 | -7.78162840138514 | 0.001 | 0.092 | 3.02668964521017E-40 | DOWN-regulation |
| AC064805.1 | -7.77441787994936 | 0 | 0.026 | 2.60311300535153E-09 | DOWN-regulation |
| LINC00968 | -7.75571751010427 | 0 | 0.02 | 2.14943018700012E-06 | DOWN-regulation |
| AC092746.1 | -7.6790936930283 | 0 | 0.016 | 0.0000900379809437504 | DOWN-regulation |
| AC087741.1 | -7.66780940952549 | 0 | 0.019 | 4.53470365320289E-06 | DOWN-regulation |
| CDC42EP1 | -7.63822732051355 | 0 | 0.022 | 1.08614965325667E-07 | DOWN-regulation |
| PLA2G7 | -7.63283945886061 | 0 | 0.023 | 5.15030092120259E-08 | DOWN-regulation |
| S100A12 | -7.60664568258003 | 0.01 | 0.646 | 0 | DOWN-regulation |
| SLC24A4 | -7.60122766835846 | 0.001 | 0.12 | 1.4639357009314E-53 | DOWN-regulation |
| HP | -7.59534335646936 | 0 | 0.047 | 1.13593059078056E-18 | DOWN-regulation |

**Table S21.** Top 50 up- and down-regulated genes in clusters expressing CD34 and PROM1 genes (clusters 1, 4, and 11) in comparison to clusters expressing cell differentiation markers (CD2, CD4, CD14) (clusters 0, 2, and 14) in CD133+Lin-CD45+ cells.

| **gene** | **avg_log2FC** | **pct.1** | **pct.2** | **p_val_adj** | **expression** |
| --- | --- | --- | --- | --- | --- |
| MFAP4 | 8.64802497154523 | 0.07 | 0 | 1.54542152536919E-36 | UP-regulation |
| AC026124.1 | 7.96554288961285 | 0.047 | 0 | 2.81298565465495E-23 | UP-regulation |
| MFAP2 | 7.95072347680273 | 0.044 | 0 | 9.3249373742384E-22 | UP-regulation |
| PRDM16-DT | 7.8711756128473 | 0.046 | 0 | 9.04046727839769E-23 | UP-regulation |
| BAALC-AS2 | 7.83396065906968 | 0.043 | 0 | 2.99284712250385E-21 | UP-regulation |
| CRISPLD1 | 7.81402179856879 | 0.044 | 0 | 9.3249373742384E-22 | UP-regulation |
| CNMD | 7.72278390171589 | 0.037 | 0 | 1.03787107933659E-17 | UP-regulation |
| DOK4 | 7.70666248452643 | 0.039 | 0 | 1.0128535305829E-18 | UP-regulation |
| FAT3 | 7.56582754061632 | 0.015 | 0 | 0.0000131979630723627 | UP-regulation |
| AC105114.2 | 7.4615743676313 | 0.058 | 0 | 1.75511068034671E-28 | UP-regulation |
| LRRC63 | 7.27784546489959 | 0.026 | 0 | 1.17221610500757E-11 | UP-regulation |
| TNFRSF4 | 7.24751685089901 | 0.025 | 0 | 3.7402678868139E-11 | UP-regulation |
| C2orf66 | 7.24443350473656 | 0.024 | 0 | 1.19339312263435E-10 | UP-regulation |
| LINC02767 | 7.21392581680146 | 0.027 | 0 | 3.67350839469211E-12 | UP-regulation |
| WIPF3 | 7.20781826487556 | 0.023 | 0 | 3.80775916652949E-10 | UP-regulation |
| CHRDL1 | 7.17243501362245 | 0.057 | 0 | 5.67616466766504E-28 | UP-regulation |
| DNAJC12 | 7.14593621579731 | 0.029 | 0 | 3.60637763058828E-13 | UP-regulation |
| AC105942.1 | 7.11426238863111 | 0.026 | 0 | 1.17221610500757E-11 | UP-regulation |
| AL391840.3 | 7.09085353875645 | 0.025 | 0 | 3.7402678868139E-11 | UP-regulation |
| MIR1915HG | 6.94027049290481 | 0.025 | 0 | 3.7402678868139E-11 | UP-regulation |
| TCEAL5 | 6.89219004154402 | 0.022 | 0 | 3.87744198607967E-09 | UP-regulation |
| TUB | 6.86301941092121 | 0.02 | 0 | 3.9512582820245E-08 | UP-regulation |
| AC021504.1 | 6.83769054969716 | 0.015 | 0 | 0.0000131979630723627 | UP-regulation |
| HOXB5 | 6.79788772521733 | 0.021 | 0 | 1.23761701411074E-08 | UP-regulation |
| AC010745.5 | 6.78561448437839 | 0.018 | 0 | 4.03182308755037E-07 | UP-regulation |
| CCDC196 | 6.78511467852493 | 0.02 | 0 | 3.9512582820245E-08 | UP-regulation |
| CGREF1 | 6.74445711350206 | 0.021 | 0 | 1.23761701411074E-08 | UP-regulation |
| AC108749.1 | 6.71651465967686 | 0.079 | 0 | 3.26571168244976E-40 | UP-regulation |
| TIE1 | 6.68637540287755 | 0.102 | 0.001 | 8.51237028481464E-53 | UP-regulation |
| AC011139.1 | 6.62308258149768 | 0.015 | 0 | 0.0000131979630723627 | UP-regulation |
| RNF150 | 6.57090428881811 | 0.108 | 0 | 2.43756421888086E-57 | UP-regulation |
| TTC29 | 6.52942767005841 | 0.016 | 0 | 4.12272725060268E-06 | UP-regulation |
| LINC01268 | 6.50463918484675 | 0.019 | 0 | 1.26190695470246E-07 | UP-regulation |
| CLCA4-AS1 | 6.46408677741433 | 0.014 | 0 | 0.0000422903814728008 | UP-regulation |
| CREG2 | 6.39501984615006 | 0.015 | 0 | 0.0000131979630723627 | UP-regulation |
| MACC1-AS1 | 6.3760862959743 | 0.013 | 0 | 0.000435817098013757 | UP-regulation |
| ESYT3 | 6.35445790465794 | 0.014 | 0 | 0.0000422903814728008 | UP-regulation |
| AC015908.2 | 6.34939983002768 | 0.014 | 0 | 0.000135666781075668 | UP-regulation |
| AC005599.1 | 6.34213984210795 | 0.011 | 0 | 0.00452150300835591 | UP-regulation |
| RIMS1 | 6.34095783102384 | 0.014 | 0 | 0.0000422903814728008 | UP-regulation |
| UMODL1-AS1 | 6.31942091556264 | 0.015 | 0 | 0.0000131979630723627 | UP-regulation |
| AP000695.2 | 6.31388791984173 | 0.014 | 0 | 0.0000422903814728008 | UP-regulation |
| CENPVL3 | 6.26604696966632 | 0.014 | 0 | 0.0000422903814728008 | UP-regulation |
| AC006008.1 | 6.26134423565951 | 0.013 | 0 | 0.000435817098013757 | UP-regulation |
| GYG2 | 6.23647370354369 | 0.015 | 0 | 0.0000131979630723627 | UP-regulation |
| KCNMB2 | 6.17227852607394 | 0.012 | 0 | 0.00140235173911973 | UP-regulation |
| AC025419.1 | 6.13889327773069 | 0.01 | 0 | 0.0146140471331365 | UP-regulation |
| LRRTM3 | 6.13163700584287 | 0.06 | 0.001 | 4.35317362453819E-29 | UP-regulation |
| RLN2 | 6.1239854099479 | 0.013 | 0 | 0.000435817098013757 | UP-regulation |
| AP000251.1 | 6.08020153308827 | 0.013 | 0 | 0.000435817098013757 | UP-regulation |
| CLEC4E | -10.1101076444252 | 0 | 0.162 | 2.06474163459463E-41 | DOWN-regulation |
| IGLC2 | -9.94529477030865 | 0 | 0.028 | 0.000531444186565692 | DOWN-regulation |
| LIPN | -9.19025788230021 | 0 | 0.102 | 6.36683802259629E-24 | DOWN-regulation |
| VNN3 | -9.15833790172255 | 0 | 0.07 | 3.83245282129734E-15 | DOWN-regulation |
| ALDH1A2 | -9.10690048133061 | 0.001 | 0.143 | 4.92141854792579E-35 | DOWN-regulation |
| CSTA | -9.08980510774473 | 0.002 | 0.377 | 4.39996997743168E-116 | DOWN-regulation |
| AHSP | -9.05930179890679 | 0.002 | 0.045 | 2.51433010858151E-07 | DOWN-regulation |
| GZMB | -9.04616000844924 | 0 | 0.033 | 0.0000240824952843485 | DOWN-regulation |
| CCL3 | -9.0045015424256 | 0 | 0.053 | 1.13855724769883E-10 | DOWN-regulation |
| HP | -8.96602580365627 | 0 | 0.056 | 2.64634070369695E-11 | DOWN-regulation |
| VCAN | -8.94817120267284 | 0.011 | 0.514 | 4.08557392392583E-173 | DOWN-regulation |
| LINC01506 | -8.94475061598923 | 0 | 0.049 | 1.63634971711538E-09 | DOWN-regulation |
| SIRPB1 | -8.78516695588639 | 0 | 0.08 | 7.54970803126524E-18 | DOWN-regulation |
| HCAR3 | -8.78489979111532 | 0 | 0.057 | 1.27395959339996E-11 | DOWN-regulation |
| HMOX1 | -8.76302122949445 | 0 | 0.089 | 2.94348319903577E-20 | DOWN-regulation |
| LGALS2 | -8.7104376546504 | 0.005 | 0.432 | 2.21422569979096E-137 | DOWN-regulation |
| CLIC3 | -8.69814255327557 | 0 | 0.038 | 8.50260062641438E-07 | DOWN-regulation |
| CYBB | -8.65002824815094 | 0.004 | 0.443 | 2.18671908002354E-143 | DOWN-regulation |
| RBP7 | -8.62022602413837 | 0.002 | 0.257 | 5.22742928293297E-71 | DOWN-regulation |
| FOLR3 | -8.614526131206 | 0 | 0.059 | 4.79916393410562E-12 | DOWN-regulation |
| CLEC4D | -8.50500308423476 | 0 | 0.055 | 4.30595230445059E-11 | DOWN-regulation |
| THBS1 | -8.49652946075146 | 0.005 | 0.363 | 4.40943152467236E-109 | DOWN-regulation |
| U62317.4 | -8.47087326006728 | 0 | 0.059 | 4.79916411492591E-12 | DOWN-regulation |
| SIGLEC9 | -8.46866081169545 | 0 | 0.059 | 4.79916429574642E-12 | DOWN-regulation |
| TLR8 | -8.46493899140593 | 0 | 0.073 | 5.26418471318278E-16 | DOWN-regulation |
| PCED1B-AS1 | -8.43317478705832 | 0.001 | 0.119 | 4.72094986226547E-28 | DOWN-regulation |
| SERPINB2 | -8.42992922929492 | 0 | 0.056 | 2.07426013099381E-11 | DOWN-regulation |
| CD86 | -8.40489370576597 | 0.001 | 0.188 | 1.09530740996083E-48 | DOWN-regulation |
| IL32 | -8.40113096929963 | 0.001 | 0.109 | 3.32829933494E-25 | DOWN-regulation |
| S100A12 | -8.38501869321012 | 0.021 | 0.555 | 2.02325659932467E-189 | DOWN-regulation |
| MS4A1 | -8.33101395778026 | 0 | 0.029 | 0.000205288901041627 | DOWN-regulation |
| OLIG1 | -8.31842174392498 | 0 | 0.074 | 4.10511227062609E-16 | DOWN-regulation |
| PID1 | -8.30613199006905 | 0.003 | 0.289 | 8.33975777811766E-82 | DOWN-regulation |
| SDC2 | -8.28469392545289 | 0 | 0.059 | 2.94361197300087E-12 | DOWN-regulation |
| MS4A6A | -8.2790490604053 | 0.003 | 0.358 | 8.04260335462293E-108 | DOWN-regulation |
| S100P | -8.25306167855539 | 0.002 | 0.109 | 1.07676312159059E-24 | DOWN-regulation |
| HSPA6 | -8.23876526103651 | 0 | 0.039 | 5.26717597432064E-07 | DOWN-regulation |
| FCGR2A | -8.20270373832184 | 0.002 | 0.229 | 1.75676688292043E-61 | DOWN-regulation |
| BPI | -8.19641892587365 | 0 | 0.027 | 0.000854889734565891 | DOWN-regulation |
| GNLY | -8.12737669144755 | 0.005 | 0.055 | 1.70779818281581E-08 | DOWN-regulation |
| FCN1 | -8.1175272581161 | 0.013 | 0.556 | 8.78806872300723E-194 | DOWN-regulation |
| CD163 | -8.08084683408969 | 0 | 0.069 | 8.05223037568577E-15 | DOWN-regulation |
| SERPINA1 | -8.07805204689355 | 0.002 | 0.359 | 2.00274274089664E-108 | DOWN-regulation |
| LAMB3 | -8.07364103015453 | 0 | 0.05 | 7.92000908374779E-10 | DOWN-regulation |
| KYNU | -8.04364203230471 | 0.005 | 0.364 | 1.52137739756211E-109 | DOWN-regulation |
| SMIM25 | -8.03375374106674 | 0.002 | 0.195 | 2.01648340195887E-50 | DOWN-regulation |
| SLC15A3 | -8.01599687799318 | 0.001 | 0.134 | 1.90275874453395E-32 | DOWN-regulation |
| MPEG1 | -7.98380893893437 | 0.002 | 0.25 | 1.746769293356E-68 | DOWN-regulation |
| GZMA | -7.96518571684191 | 0.001 | 0.055 | 1.12745377329999E-10 | DOWN-regulation |
| CD1D | -7.95390191862632 | 0 | 0.064 | 1.98515001220701E-13 | DOWN-regulation |

**Table S22.** Top 50 up and down-regulated genes in cluster 0 in CD133+Lin-CD45+ cells.

| **gene** | **avg_log2FC** | **pct.1** | **pct.2** | **p_val_adj** | **expression** |
| --- | --- | --- | --- | --- | --- |
| C1QA | 6.64552789356881 | 0.019 | 0 | 1.92860135413272E-07 | UP-regulation |
| AL121885.1 | 6.59253960691788 | 0.012 | 0 | 0.000192534404065737 | UP-regulation |
| CLEC10A | 6.16880807273817 | 0.053 | 0.001 | 3.17402691420412E-27 | UP-regulation |
| GPRC5A | 5.68894524891714 | 0.014 | 0 | 0.0000918536794263259 | UP-regulation |
| RHBDL3 | 5.39050154591738 | 0.022 | 0 | 1.91984449550882E-09 | UP-regulation |
| TMEM176A | 5.26659581353258 | 0.09 | 0.003 | 5.89683406247508E-44 | UP-regulation |
| C1orf53 | 4.92606895441332 | 0.011 | 0 | 0.00943514746122767 | UP-regulation |
| TMEM176B | 4.61432581800292 | 0.129 | 0.004 | 6.04784812249227E-64 | UP-regulation |
| SIGLEC1 | 4.52907330636655 | 0.012 | 0 | 0.00203110545677697 | UP-regulation |
| CTTNBP2 | 4.50664568589653 | 0.016 | 0.001 | 0.000155650096768618 | UP-regulation |
| SASH1 | 4.38343438711788 | 0.2 | 0.009 | 3.81629354713548E-97 | UP-regulation |
| PID1 | 4.31144919194451 | 0.686 | 0.04 | 0 | UP-regulation |
| TMEM121B | 4.29830023708777 | 0.012 | 0.001 | 0.0144138635178207 | UP-regulation |
| AC073352.2 | 4.24699058994344 | 0.01 | 0 | 0.0446791462831841 | UP-regulation |
| RNF152 | 4.16243375227667 | 0.012 | 0.001 | 0.0144404703162128 | UP-regulation |
| TM4SF19 | 4.11688678392267 | 0.013 | 0.001 | 0.00322788820418099 | UP-regulation |
| SEMA6B | 4.09946881205342 | 0.049 | 0.003 | 4.7873210974792E-20 | UP-regulation |
| SYN1 | 4.02327719775266 | 0.03 | 0.002 | 1.34010027925E-10 | UP-regulation |
| MARCO | 3.91069546668107 | 0.112 | 0.006 | 2.00583841419608E-49 | UP-regulation |
| C15orf48 | 3.90024761389457 | 0.057 | 0.003 | 9.63352812492219E-24 | UP-regulation |
| EPHB3 | 3.84348245664915 | 0.014 | 0.001 | 0.000725655291722671 | UP-regulation |
| NID1 | 3.80053165411143 | 0.042 | 0.002 | 9.47535259636448E-18 | UP-regulation |
| TM4SF20 | 3.76524842979634 | 0.023 | 0.001 | 2.84107279004599E-08 | UP-regulation |
| AC007785.1 | 3.75920920435743 | 0.024 | 0.001 | 3.98923381841344E-08 | UP-regulation |
| AC092723.4 | 3.72935121169798 | 0.013 | 0.001 | 0.0179379130538005 | UP-regulation |
| PDK4 | 3.67078026457264 | 0.013 | 0 | 0.000438540920532773 | UP-regulation |
| CSF1R | 3.52532984839165 | 0.311 | 0.018 | 1.44935713395011E-146 | UP-regulation |
| FPR3 | 3.44342147310947 | 0.013 | 0.001 | 0.00329499625351146 | UP-regulation |
| EPHB2 | 3.42843862480646 | 0.027 | 0.001 | 2.05480117252896E-09 | UP-regulation |
| AL669970.3 | 3.42744670423241 | 0.019 | 0.001 | 0.0000633089266483058 | UP-regulation |
| NRG1 | 3.40458201717781 | 0.197 | 0.014 | 1.65765209601005E-83 | UP-regulation |
| LINC02356 | 3.38270288500142 | 0.028 | 0.002 | 1.45234778783761E-08 | UP-regulation |
| AC105105.1 | 3.37499133529228 | 0.018 | 0.001 | 0.000269526868513514 | UP-regulation |
| TNNT1 | 3.3499729106131 | 0.032 | 0.003 | 1.045104067019E-09 | UP-regulation |
| TPPP3 | 3.33821192672621 | 0.153 | 0.01 | 5.05338789446639E-66 | UP-regulation |
| EMP1 | 3.32825431926268 | 0.119 | 0.016 | 3.051942014063E-37 | UP-regulation |
| HAS1 | 3.31484914584152 | 0.089 | 0.01 | 2.26167085845462E-29 | UP-regulation |
| GAS2L3 | 3.28972360741021 | 0.075 | 0.007 | 8.36807323899411E-27 | UP-regulation |
| CPVL | 3.27681075469214 | 0.537 | 0.054 | 3.37537894935132E-233 | UP-regulation |
| PLA2G7 | 3.24154935712727 | 0.024 | 0.002 | 1.09771683231363E-06 | UP-regulation |
| TRIM36 | 3.19339653003831 | 0.022 | 0.003 | 0.0000712848856025242 | UP-regulation |
| LGALS2 | 3.19076381104405 | 0.889 | 0.105 | 0 | UP-regulation |
| VENTX | 3.18953902892697 | 0.037 | 0.003 | 3.48208481528834E-12 | UP-regulation |
| SLC5A4-AS1 | 3.18497026923713 | 0.038 | 0.003 | 1.62212132658534E-13 | UP-regulation |
| CES1 | 3.17788531663789 | 0.022 | 0.001 | 7.97944457816992E-07 | UP-regulation |
| EPB41L3 | 3.17787039260136 | 0.464 | 0.071 | 8.07587840067635E-167 | UP-regulation |
| RYR1 | 3.17690826903031 | 0.035 | 0.003 | 2.3573859098656E-10 | UP-regulation |
| ARHGEF10L | 3.15633961790198 | 0.139 | 0.009 | 4.49191857395732E-59 | UP-regulation |
| GPC4 | 3.15549645959619 | 0.012 | 0.001 | 0.0149275658035448 | UP-regulation |
| BATF3 | 3.13449915332718 | 0.075 | 0.005 | 2.42193487033954E-30 | UP-regulation |
| IGKC | -10.32240842 | 0 | 0.029 | 0.006253945 | DOWN-regulation |
| CARD11 | -9.175604963 | 0 | 0.135 | 1.31981E-26 | DOWN-regulation |
| TRAC | -8.525898422 | 0 | 0.074 | 1.23102E-12 | DOWN-regulation |
| CD79A | -8.367928371 | 0 | 0.051 | 1.00546E-07 | DOWN-regulation |
| ZNF827 | -8.362355357 | 0 | 0.115 | 5.83528E-22 | DOWN-regulation |
| CLIC3 | -8.278338336 | 0 | 0.036 | 0.000245893 | DOWN-regulation |
| IL7R | -8.199644151 | 0.002 | 0.122 | 1.25274E-22 | DOWN-regulation |
| ADGRG1 | -8.160228917 | 0 | 0.114 | 1.05962E-21 | DOWN-regulation |
| SELENBP1 | -7.980700373 | 0 | 0.034 | 0.000505294 | DOWN-regulation |
| LTF | -7.980104314 | 0.002 | 0.03 | 0.035501384 | DOWN-regulation |
| CCR7 | -7.941885577 | 0 | 0.06 | 1.46629E-09 | DOWN-regulation |
| CD6 | -7.891593156 | 0 | 0.058 | 3.68936E-09 | DOWN-regulation |
| MME | -7.88612192 | 0 | 0.033 | 0.000866841 | DOWN-regulation |
| CRYGD | -7.879383737 | 0 | 0.104 | 2.65331E-19 | DOWN-regulation |
| TCL1A | -7.738472722 | 0 | 0.026 | 0.026270681 | DOWN-regulation |
| LEF1 | -7.735435942 | 0.001 | 0.098 | 1.56907E-17 | DOWN-regulation |
| IKZF3 | -7.657286178 | 0 | 0.048 | 5.20023E-07 | DOWN-regulation |
| PYHIN1 | -7.593007955 | 0 | 0.038 | 8.33503E-05 | DOWN-regulation |
| ZAP70 | -7.486424516 | 0 | 0.05 | 2.50704E-07 | DOWN-regulation |
| IGHM | -7.486337193 | 0.003 | 0.223 | 1.11409E-47 | DOWN-regulation |
| CST7 | -7.451356987 | 0.001 | 0.065 | 3.52311E-10 | DOWN-regulation |
| AKR1C3 | -7.443656544 | 0 | 0.071 | 4.58195E-12 | DOWN-regulation |
| ZNF667 | -7.42740672 | 0 | 0.074 | 1.01992E-12 | DOWN-regulation |
| BSPRY | -7.421693717 | 0 | 0.085 | 3.4582E-15 | DOWN-regulation |
| GYPB | -7.419173716 | 0 | 0.03 | 0.003649731 | DOWN-regulation |
| CD247 | -7.403549967 | 0.002 | 0.112 | 2.85033E-20 | DOWN-regulation |
| PGLYRP1 | -7.375442177 | 0.001 | 0.034 | 0.001354957 | DOWN-regulation |
| CPA3 | -7.363070228 | 0 | 0.033 | 0.000866841 | DOWN-regulation |
| CD28 | -7.356439735 | 0 | 0.043 | 7.94182E-06 | DOWN-regulation |
| LRRN3 | -7.346313098 | 0 | 0.045 | 2.67397E-06 | DOWN-regulation |
| CD34 | -7.344726417 | 0.001 | 0.189 | 1.54349E-39 | DOWN-regulation |
| GZMB | -7.288305226 | 0.001 | 0.03 | 0.011715873 | DOWN-regulation |
| MEST | -7.257114054 | 0 | 0.076 | 3.97628E-13 | DOWN-regulation |
| LINC02232 | -7.236419994 | 0 | 0.07 | 9.69063E-12 | DOWN-regulation |
| KRT18 | -7.235069553 | 0 | 0.06 | 1.76375E-09 | DOWN-regulation |
| AC107223,1 | -7.191495639 | 0 | 0.057 | 5.33328E-09 | DOWN-regulation |
| LRP6 | -7.168462721 | 0 | 0.058 | 3.06815E-09 | DOWN-regulation |
| KCNK17 | -7.151834856 | 0 | 0.057 | 6.41155E-09 | DOWN-regulation |
| COL6A2 | -7.143175527 | 0 | 0.055 | 1.60799E-08 | DOWN-regulation |
| HIST1H2BF | -7.13818399 | 0 | 0.062 | 5.8155E-10 | DOWN-regulation |
| GZMA | -7.135063096 | 0.001 | 0.051 | 3.85453E-07 | DOWN-regulation |
| DYTN | -7.10847026 | 0 | 0.064 | 2.30165E-10 | DOWN-regulation |
| HIP1R | -7.037729564 | 0 | 0.05 | 2.50704E-07 | DOWN-regulation |
| LCK | -7.029979584 | 0.001 | 0.096 | 5.08966E-17 | DOWN-regulation |
| AL355612,1 | -7.024113328 | 0 | 0.047 | 7.48626E-07 | DOWN-regulation |
| CMBL | -6.995947598 | 0 | 0.062 | 5.8155E-10 | DOWN-regulation |
| BLK | -6.984818449 | 0 | 0.026 | 0.026270682 | DOWN-regulation |
| HMGA2 | -6.976991199 | 0.002 | 0.223 | 3.23917E-48 | DOWN-regulation |
| LBH | -6.973002293 | 0.001 | 0.103 | 1.05479E-18 | DOWN-regulation |
| MYEF2 | -6.971738164 | 0 | 0.06 | 1.76375E-09 | DOWN-regulation |
