# Supplementary figures and images for: Optimizing single cell RNA sequencing of stem cells. A streamlined workflow for enhanced sensitivity and reproducibility in hematopoietic studies. The use of human umbilical cord blood-derived hematopoietic stem and progenitor cells
Source: Front Cell Dev Biol. 2025 May 15;13:1590889. doi: 10.3389/fcell.2025.1590889 (PMC12119605; doi:10.3389/fcell.2025.1590889)

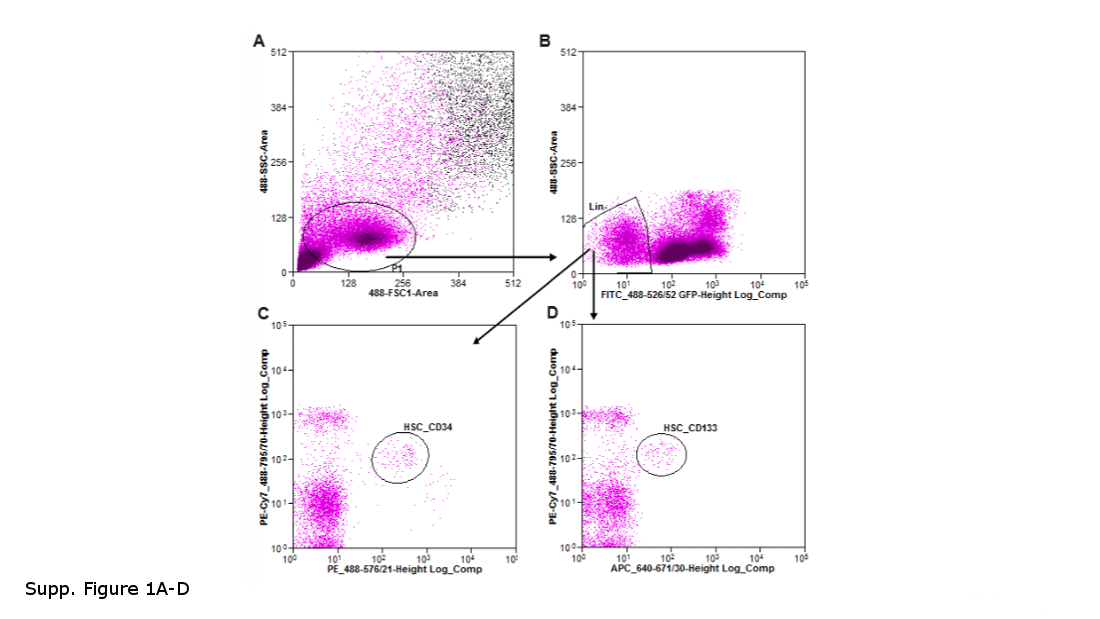

Supplement: Supplementary file 2 [file DataSheet1.zip › Supplementary figure 1.TIFF]

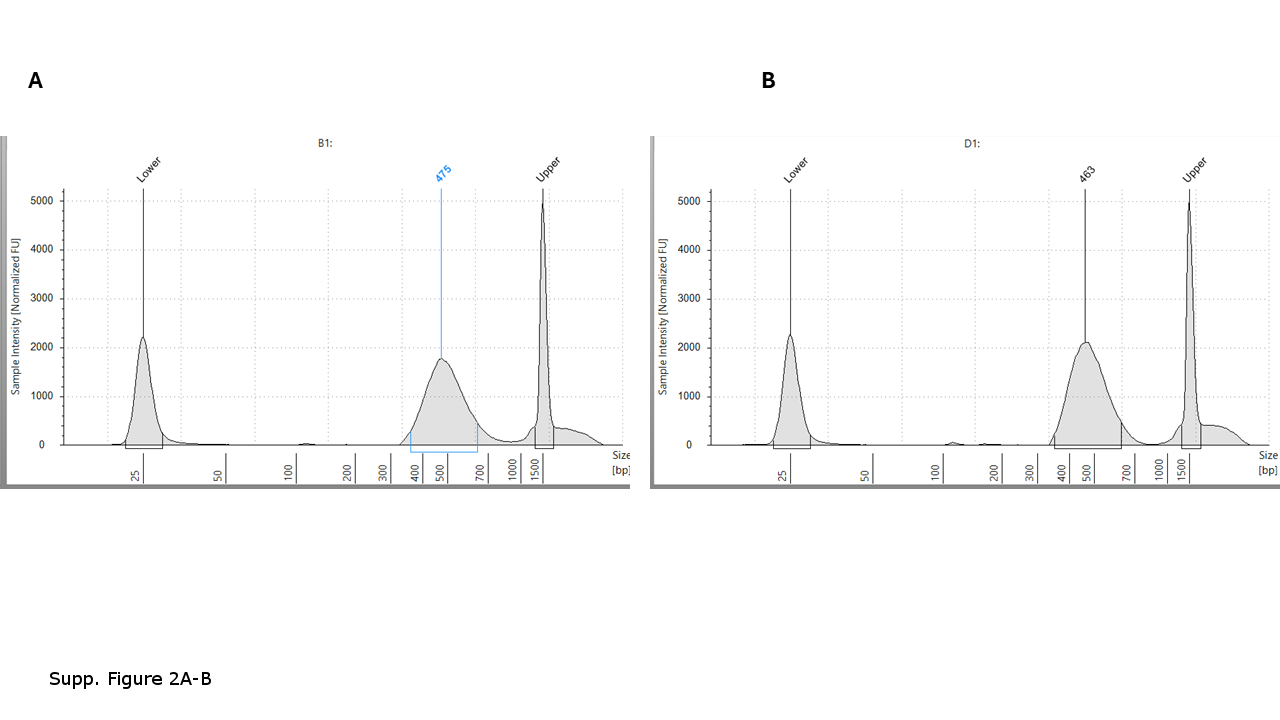

Supplement: Supplementary file 2 [file DataSheet1.zip › Supplementary figure 2A-B.TIF]

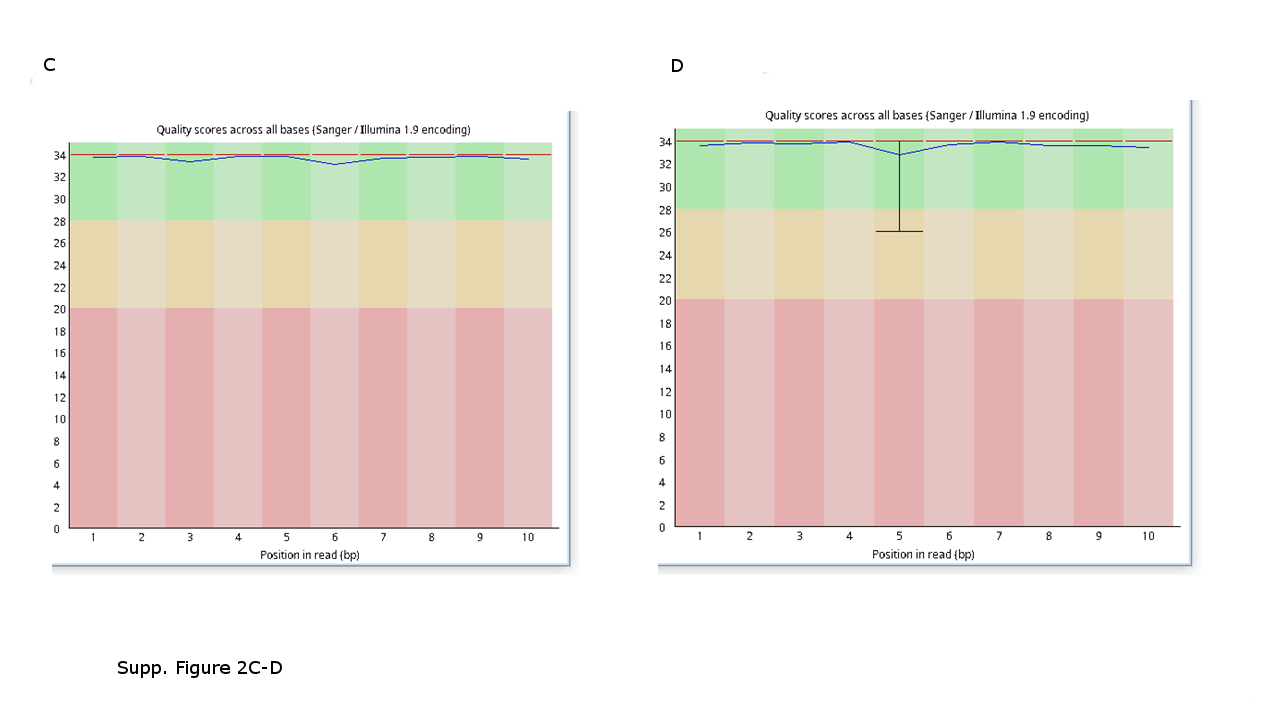

Supplement: Supplementary file 2 [file DataSheet1.zip › Supplementary figure 2C-D.TIF]

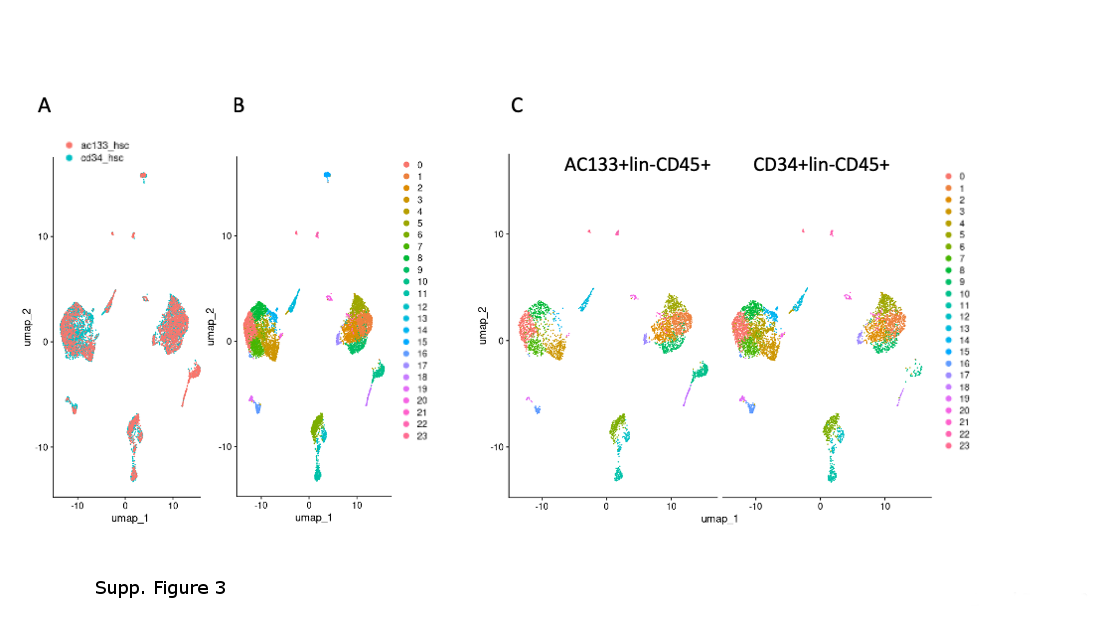

Supplement: Supplementary file 2 [file DataSheet1.zip › Supplementary figure 3.TIFF]
